# Effects of changes on gut microbiota in children with acute Kawasaki disease

**DOI:** 10.7717/peerj.9698

**Published:** 2020-08-06

**Authors:** Jie Shen, Yinghe Ding, Zuocheng Yang, Xueyan Zhang, Mingyi Zhao

**Affiliations:** Department of Pediatrics, The Third Xiangya Hospital, Central South University, Changsha, Hunan Province, China

**Keywords:** Kawasaki disease, Gut microbiota, High-throughput sequencing analysis, Dorea, Hydrogen

## Abstract

**Background:**

Kawasaki disease (KD) is an acute febrile illness of early childhood. The exact etiology of the disease remains unknown. At present, research on KD is mostly limited to susceptibility genes, infections, and immunity. However, research on the correlation between gut microbiota and KD is rare.

**Methods:**

Children with a diagnosis of acute KD and children undergoing physical examination during the same period were included. At the time of admission, the subjects’ peripheral venous blood and feces were collected. Faecal samples were analyzed for bacterial taxonomic content via high-throughput sequencing. The abundance, diversity, composition, and characteristic differences of the gut microbiota in KD and healthy children were compared by alpha diversity, beta diversity, linear discriminant analysis and LDA effect size analysis. Blood samples were used for routine blood examination, biochemical analysis, and immunoglobulin quantitative detection.

**Results:**

Compared with the control group, the community richness and structure of gut microbiota in the KD group was significantly reduced (Chao1 richness estimator, mean 215.85 in KD vs. mean 725.76 in control, *p* < 0.01; Shannon diversity index, mean 3.32 in KD vs. mean 5.69 in control, *p* < 0.05). LEfSe analysis identified two strains of bacteria significantly associated with KD: *Bacteroidetes* and *Dorea*. *Bacteroidetes* were enriched in healthy children (mean 0.16 in KD vs. mean 0.34 in control, *p* < 0.05). *Dorea* was also enriched in healthy children but rarely existed in children with KD (mean 0.002 in KD vs. mean 0.016 in control, *p* < 0.05). Compared with the control, IgA and IgG in the KD group decreased (IgA, median 0.68 g/L in KD vs. median 1.06 g/L in control, *p* < 0.001; IgG, median 6.67 g/L in KD vs. median 9.71 g/L in control, *p* < 0.001), and IgE and IgM levels were not significantly changed.

**Conclusions:**

Dysbiosis of gut microbiota occurs in children with acute KD and may be related to the etiology or pathogenesis of KD. It is worth noting that for the first time, we found that *Dorea*, a hydrogen-producing bacterium, was significantly reduced in children with acute KD. Overall, our results provide a theoretical basis for the prevention or diagnosis of KD based on intestinal microecology.

## Introduction

Kawasaki disease (KD), which is also known as mucocutaneous lymph node syndrome (MCLS), is a self-limited vasculitis of childhood. Clinical manifestations of KD include prolonged fever (1–2 weeks, mean 10–11 days), conjunctival injection, oral lesions, polymorphous skin rashes, extremity changes, and cervical lymphadenopathy. Most children recover naturally, but some more severely affected patients exhibit cardiac complications, particularly coronary artery lesions (CALs), such as aneurysms and ectasias.

At present, the etiology of KD has not been completely elucidated, and research has focused on genetics, infection, and immunity. It is generally accepted that KD is an immune-mediated vasculitis disease caused by one or more specific pathogens infecting susceptible populations (especially Asians) with a genetic predisposition. Although the incidence rate of KD exhibited obvious racial differences and familial aggregation (Nakamura et al., 2012), no pathogenic gene has been found. At present, the superantigen theory is the leading research direction of the KD infection theory. However, to date, no specific pathogen or specific superantigen has been identified that can induce KD alone or jointly (Rhim et al., 2019). However, studies have shown that the superantigen of KD may partly originate from intestinal flora (Nagata et al., 2009), and the initiating factor is likely to invade from the intestinal tract (Esposito, Polinori & Rigante, 2019).

Immune diseases are often accompanied by changes in the gut microbiota as confirmed by many studies, and one example is systemic lupus erythematosus, which is typically characterized by vasculitis, like KD (Collison, 2019). The researchers hypothesized that the pathogen of KD might be a component of the normal flora of the host, and KD may represent a hyperimmune reaction of genetically susceptible children to variants of normal environmental flora (Lee, Han & Lee, 2007).

In this study, high-throughput sequencing technology was used to detect and analyze the gut microbiota of children with acute KD and healthy children. Combined with routine blood examination, biochemical indicators, immunoglobulin level, and other indicators of the two groups, we expect to provide a new direction for subsequent studies of the etiology and pathogenesis of KD.

## Materials and Methods

### Clinical specimens

In total, 48 cases of KD (24 males and 24 females) and 46 healthy children (25 males and 21 females) were included. The following inclusion criteria were used for the KD group: (1) children with KD hospitalized in the Third Xiangya Hospital from June 2017 to December 2019; (2) KD diagnosis complies with the criteria established by KD Research Committee of American Heart Association (McCrindle Brian et al., 2017); (3) KD is in the acute phase, with fever days less than 10 days before admission. The following exclusion criteria were employed: (1) older than 12 years old; (2) history of gastrointestinal disease, genetic disease, immune disease, metabolic disease, and bacteremia; (3) history of drugs, antibiotics, probiotics or prebiotics taken within 3 months; (4) mycoplasma, virus, staphylococcus, or other pathogen infection at admission. The exclusion criteria for the control group were consistent with those for the KD group. This study was reviewed and approved by the local ethic committee of the third Xiangya Hospital of Central South University (No: 2016-S155), and informed consent was signed by parents.

### Sample collection and DNA extraction

At the time of admission, 10 mL of peripheral venous blood and 10 g of feces were collected. Laboratory parameters assessed included immunoglobulins (IgG, IgM, IgA and IgE), C3, C4, white blood cell (WBC), hemoglobin (HGB), platelets (PLT), total protein (TP), alanine aminotransferase (ALT) and aspartate aminotransferase (AST). We randomly selected three fecal samples from each of the two groups. The middle part of the fecal sample was intercepted, repacked in a sterilized frozen storage tube, and stored in −80 °C freezer until analysis. According to the manufacturer’s instructions, total DNA extraction was performed using a QIAamp^®^ DNA Stool Mini Kit (QIAGEN, Tokyo, Japan).

### Sequencing of 16S-rRNA gene amplicon

The selected region for 16S-rRNA gene amplification was v3–v4 variable region. The universal primers used were 338F (5′-ACTCCTACGGGAGGCAGCA-3′) and 806R (5′-GGACTACHVGGGTWTCTAAT-3′). After purification and quantitative detection of the amplified products, a DNA library was prepared using a TruSeq Nano DNA LT Library Prep Kit (Illumina, San Diego, CA, USA). Finally, MiSeq-PE250 was used for sequencing.

### Operational taxonomic unit demarcation and status identification

In the research field of microbial ecology, operational taxonomic unit (OTU) demarcation and status identification not only simplify the data structure but also offer more advantages to compare different sources of microbial community samples at a certain level of classification. We set 97% sequence similarity as the OTU threshold using the “UCLUST” plugin from the “QIIME” software to divide sequences into OTUs. Then, each OTU was compared with the corresponding template sequence in the database for status identification. OTU classification results were prepared as an abundance matrix for subsequent analysis.

### Alpha diversity analysis

For microbial communities, multiple indices are used to reflect their Alpha diversity. Comprehensive indices, including the Chao1 richness estimator and the Shannon diversity index, delineate community richness and diversity. With a rarefied OTU abundance matrix, all indices were calculated using QIIME software (University of Colorado, Boulder, CO, USA) and then visualized by boxplot.

### Beta diversity analysis

Partial least squares discriminant analysis (PLS-DA) based on a partial least-squares regression model was performed as a discriminant analysis of microbial community structure, which can reduce the effect of multicollinearity among variables in sample data. R software was applied for PLS-DA model construction and visualization.

### Taxonomic composition analysis

To characterize the taxonomic composition of the gut microbiome in children with KD and control, “QIIME” software was used to obtain the composition and abundance distribution table of each sample in five classification levels of phylum, class, order, family, and genus. Then, R software was used to create histograms of gut microbiome composition divided by groups in different classification levels. Subsequent comparisons of inter-group differences were performed using Metastats test.

To assess the statistically significant composition difference between control and KD, LEfSe analysis based on linear discriminant analysis (LDA) effect size was conducted. The relative abundance of the genus level matrix was submitted to the Galaxy online platform provided by Harvard University (http://huttenhower.sph.harvard.edu/galaxy/), and results were displayed concomitantly.

### Statistical analysis

Statistical analyses were used (SPSS, version 23.0, SPSS, Inc., Chicago, IL, USA) to analyze data. Values are expressed as the means ± SDs ($\bar{\rm{\chi}}\pm {\rm s}$) or medians (interquartile range). A comparison of gender distribution between the two groups was performed using the chi-square test. Here, *t* test was only applied to continuous variables with normal distribution and equal variance. Otherwise, Mann–Whitney *U* or Wilcoxon rank-sum test was used. A *p*-value of <0.05 was considered statistically significant.

## Results

### Laboratory findings at admission

No difference was noted between the two groups in terms of gender and age (2.5 ± 1.6 years vs. 3.0 ± 1.7 years). The fever duration in the KD group before admission was 5.5 (4.0–6.0) days. Table 1 shows the baseline characteristics of children. Compared with the control group, C3, C4, WBC, ALT, and AST in the KD group increased, whereas HGB and TP decreased. The results were statistically significant. There was no significant change in PLT in the KD group. Among the four immunoglobulin indicators, IgA and IgG decreased in the KD group, which was statistically significant compared with the control group. However, no significant differences in IgE and IgM levels were noted between the two groups (Table 2).

**Table 1:** Baseline characteristics.

	Control	KD	*p*
Gender, *n* (male:female)	46 (25:21)	48 (24:24)	0.673
Age, year	3.0 ± 1.7	2.5 ± 1.6	0.215
0–2	*n* = 18	*n* = 24	
3–5	*n* = 25	*n* = 22	
6–12	*n* = 3	*n* = 2	
Fever duration before admission, day		5.5 (4.0–6.0)	
2–4		*n* = 15	
5–10		*n* = 33	
Bilateral conjunctival injection		*n* = 35	
Changes of the lips and oral cavity		*n* = 37	
Changes in the extremities		*n* = 23	
Rash		*n* = 31	
Cervical lymphadenopathy		*n* = 30	
Heart complications (coronary artery abnormalities)		*n* = 4	
Intravenous immunoglobulin (IVIG)		*n* = 40	
Within 24 h after admission		*n* = 26	
Within 24–36 h after admission		*n* = 11	
Within 36–72 h after admission		*n* = 3	

**Table 2:** Laboratory findings at admission of subjects.

Parameters	Control	KD	*p*
WBC (×10^9^)	7.02 (2.54)	15.18 (5.16)	<0.001*
HGB (g/L)	125.63 ± 12.07	116.56 ± 9.68	<0.001*
PLT (×10^9^)	308.00 (105.25)	298.00 (122)	0.570
ALT (U/L)	16.00 (7.50)	30.00 (89.00)	<0.001*
AST (U/L)	26.00 (11.50)	35.00 (23.00)	<0.001*
TP (g/L)	68.37 ± 6.84	61.48 ± 5.44	<0.001*
C3 (g/L)	0.90 ± 0.18	1.13 ± 0.23	<0.001*
C4 (g/L)	0.17 (0.10)	0.26 (0.11)	<0.001*
IgA (g/L)	1.06 (0.76)	0.68 (0.70)	<0.001*
IgE (IU/ml)	47.00 (171.75)	101.00 (136.00)	0.189
IgG (g/L)	9.71 (3.47)	6.67 (3.17)	<0.001*
IgM (g/L)	1.07 (0.63)	1.15 (0.53)	0.364

**Notes:**

* Means *p* < 0.05.

C3, third component of complement; C4, fourth component of complement; WBC, white blood cell; HGB, hemoglobin; PLT, platelets; ALT, alanine aminotransferase; AST, aspartate aminotransferase; TP, total protein; IgA, immunoglobulin A; IgE, immunoglobulin E; IgG, immunoglobulin G; IgM, immunoglobulin M.

### Alpha diversity analysis

**Figure 1:**
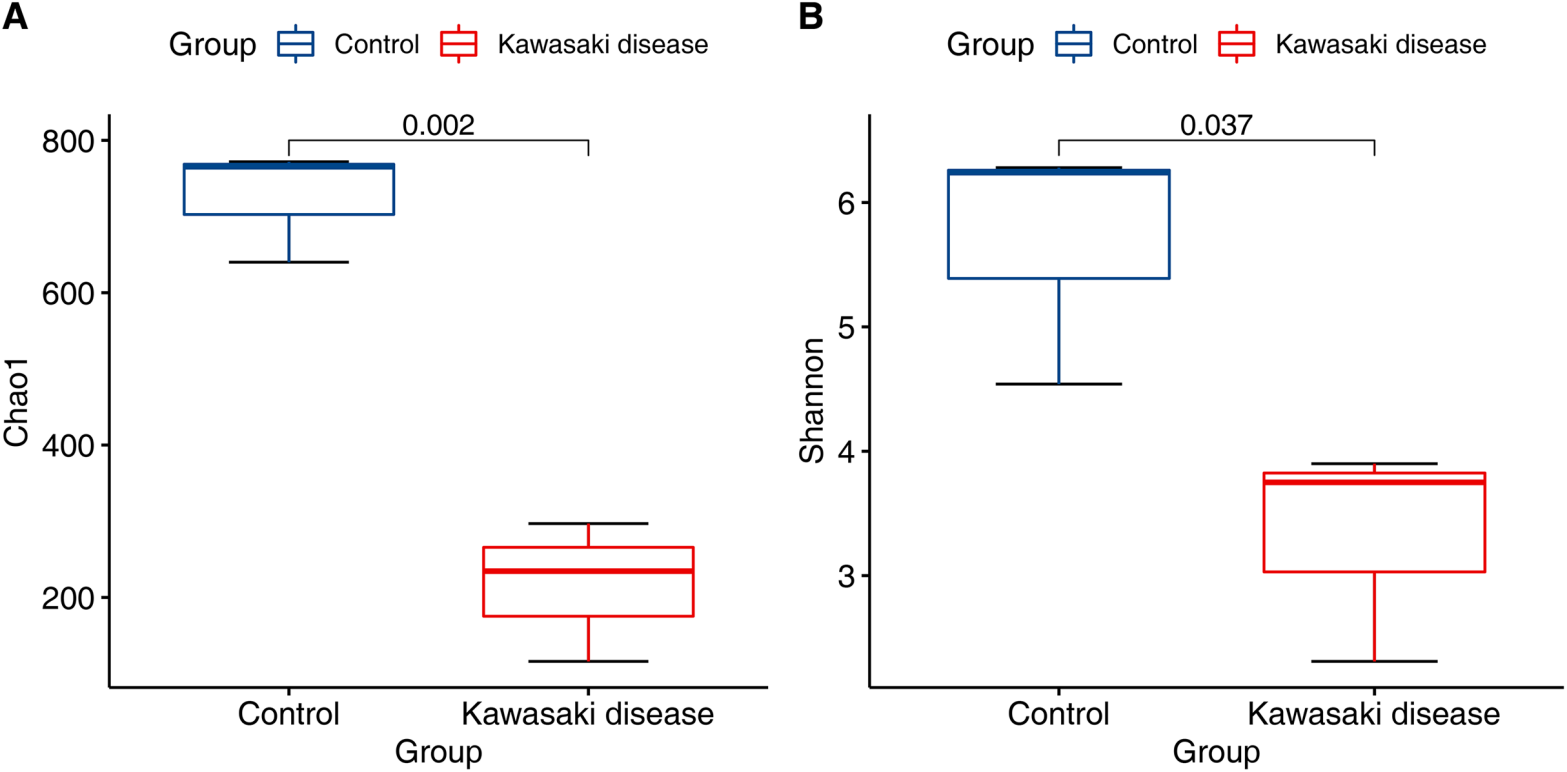
Decreased alpha diversity in children with KD. Alpha diversity evaluated by (A) Chao1 richness estimator and (B) Shannon diversity index at minimum sequencing depth level of 90% for subjects with the KD and control group. *p*-Values were calculated using the Wilcoxon rank sum test.

Compared with control, the community richness of the gut microbiome in KD significantly declined (Chao1 richness estimator, Fig. 1A; mean 215.85 (SD 75.07) in KD vs. mean 725.76 (SD 60.67) in control, *p* < 0.01). In addition to community richness, children with KD also exhibited reduced gut microbiome diversity when richness and evenness were considered together (Shannon diversity index, Fig. 1B; mean 3.32 (SD 0.72) in KD vs. mean 5.69 (SD 0.81) in control, *p* < 0.05).

### Beta diversity analysis

**Figure 2:**
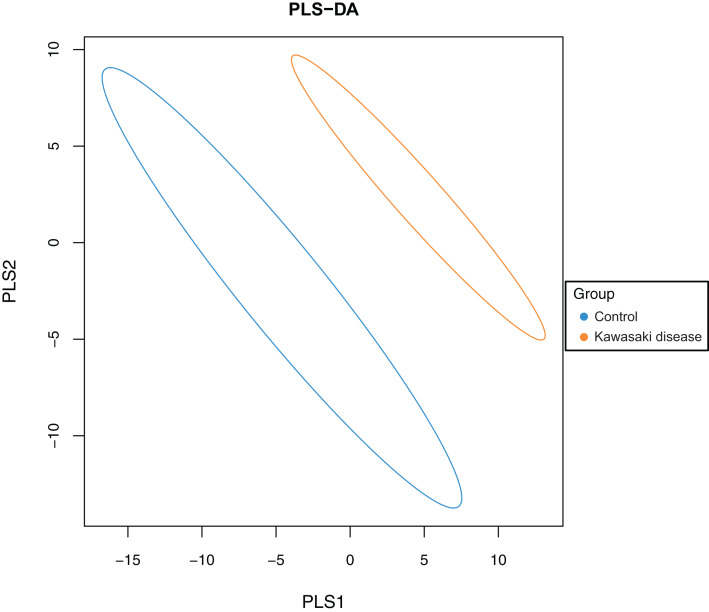
Significant beta diversity difference between the control and KD group. Each point represented a sample. Points of the same color belonged to the same group and were marked with ellipses.

To evaluate the difference in gut microbiome composition between healthy children and children with KD, PLS-DA was conducted on both groups to characterize samples on a two-dimensional surface. The visible and apparent clustering distance revealed the significantly distinct structure of the gut microbiome in children with KD compared with control (Fig. 2).

### Taxonomic composition analysis

**Figure 3:**
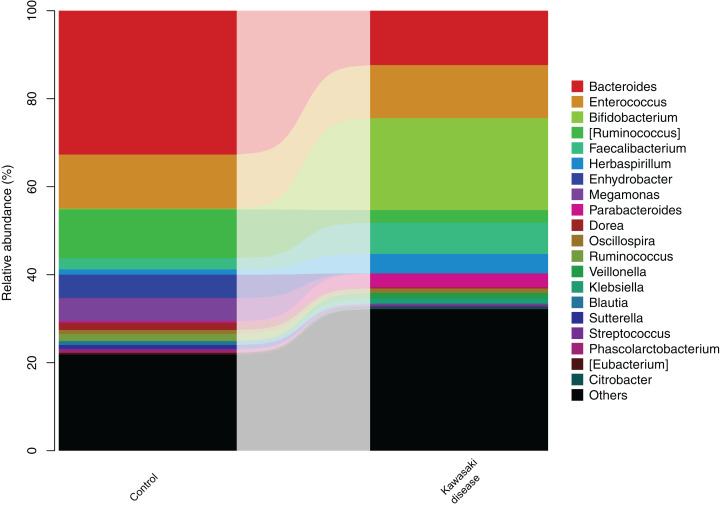
Gut microbiome structure in level of genera in the control and KD group. The height of ****bars in different colors represented the relative abundance of corresponding genus of gut microbiome.

After the significant difference in community structure was illustrated, stratified gut microbiome composition was analyzed and subsequently visualized in the form of a bar diagram. At the genus level, *Bacteroides* and *Enterococcus* were dominant bacteria in both groups, while *Bacteroides* preponderated in the control group. In addition, *Enterococcus* was enriched in KD (Fig. 3). Metastatic tests revealed that the taxa abundance of *Bacteroidetes* and *Dorea* significantly decreased in KD.

**Figure 4:**
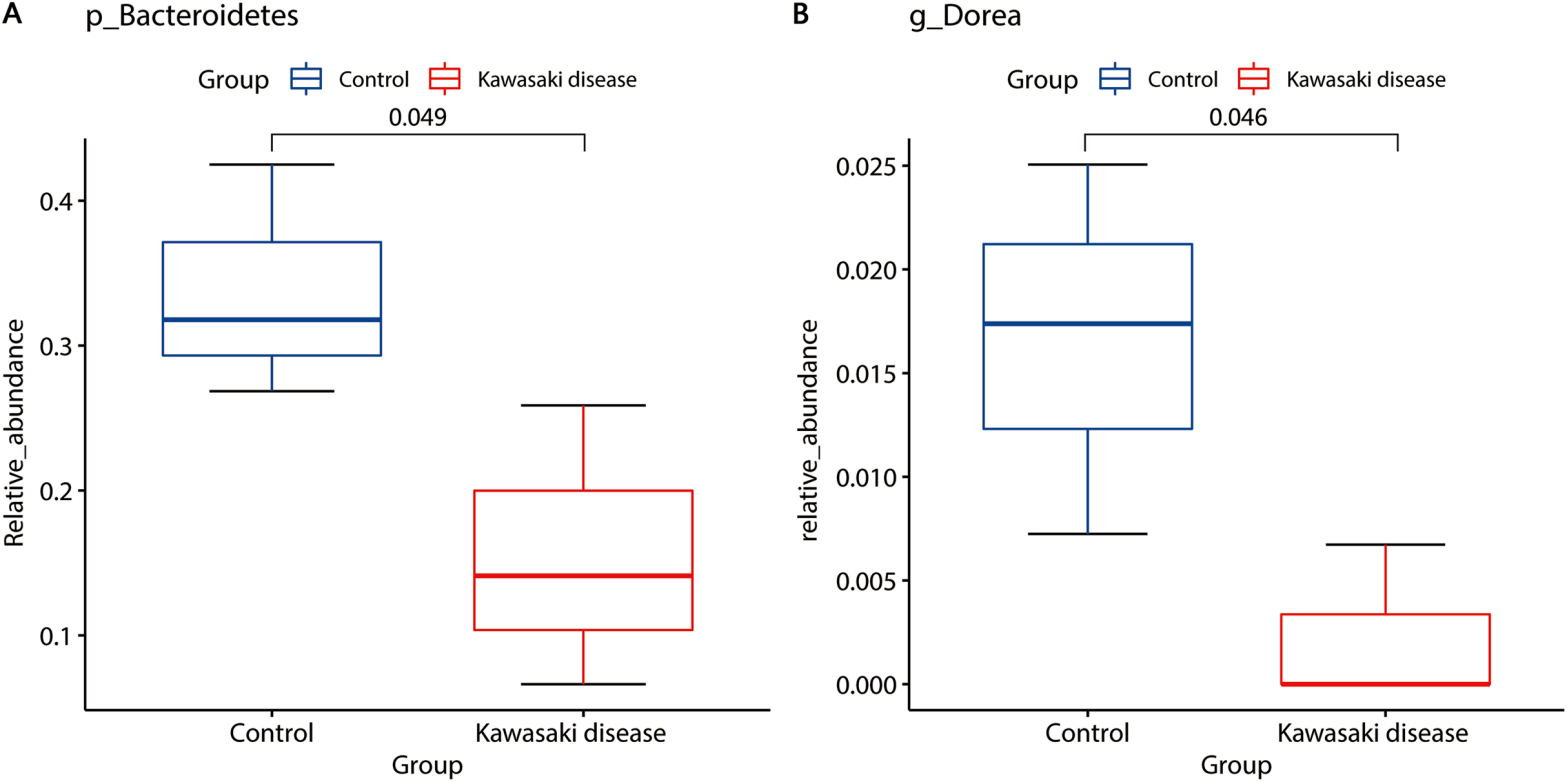
Distinct gut microbiome composition in the KD group. *Bacteroidetes* (A) and *Dorea* (B) were enriched in the control group. Only strains with Linear Discriminant Analysis (LDA) scores > 2 and *p* < 0.05 were presented. p: phylum; g: genus.

LEfSe analysis found two strains of bacteria significantly associated with KD: *Bacteroidetes* and *Dorea*. *Bacteroidetes* in level of phyla, *Bacteroidia* in level of classes, and *Bacteroidales* in level of orders were enriched in healthy children. *Dorea* was also enriched in healthy children but rarely existed in children with KD (Fig. 4).

## Discussion

The establishment of intestinal microbiota is mainly affected by genetic background, birth pattern, nutrient composition of breast milk, early diet and the use of antibiotics, and a relatively stable community distribution is not established until 3–4 years of age (Stewart et al., 2018). According to epidemiological statistics, KD is relatively rare in adults or new-borns. In total, 80% of patients experienced their first onset between 6 months and 5 years old (Holman et al., 2010). This disposition suggests that the maturing immune system in early childhood is involved in the pathogenesis of KD. This time period is when the intestinal microecology of children gradually improves and stabilizes. This feature also suggests that a special relationship occurs between KD and gut microbiota.

The relationship between intestinal microflora and the immune system is very close. Numerous studies have confirmed that intestinal microflora can have an important impact on systemic innate immunity and specific immunity (Honda & Littman, 2016). Gut microbiota can maintain the stability of intestinal mechanical, chemical and biological barriers; regulate the proliferation and differentiation of T cells and B cells in the mucosa; mediate the secretion of cytokines; and promote the production of secretory immunoglobulin A (sIgA) by plasma cells to regulate intestinal mucosal immune response. In this process, activated immune cells can migrate into the blood circulation through lymph nodes and induce a systemic immune response (Xu et al., 2019). Studies have also found that metabolites produced by gut microbiota can also affect the development and function of bone marrow and ultimately affect immune cell function (Thaiss et al., 2016).

In genetically susceptible individuals, environmental factors induce gut microbiota disorder, further affecting the differentiation of immune cell subsets and the integrity of mucosal barrier and thus leading to the breaking of immune tolerance. These effects may eventually lead to the occurrence of autoimmune diseases. Gut microbiota disorder can be detected in a variety of non-gut systemic autoimmune disease, such as rheumatoid arthritis (RA), systemic lupus erythematosus (SLE) and spondyloarthritis (SpA). A data-driven study suggested that gut microbiota and its metabolites contribute to RA at genetic, functional, and phenotypic levels (Wang & Xu, 2019). Animal models found a single commensal microbe can drive RA by promoting Th17 cells (Wu et al., 2010) or Tfh cells (Teng et al., 2016). In the gut, researchers found Ro60 ortholog-expressing commensal bacteria that can lead to SLE by triggering cross-reactivity (Greiling et al., 2018).

As an immune disease, KD is also very likely to exhibit gut microbiota disorder. Currently, studies on the correlation between KD and gut microbiota are rare. Kinumaki et al. (2015) first used metagenomic sequencing technology to analyze the gut microbiota of children with KD in the acute and follow-up periods in 2015. The results showed that the composition of gut microbiota changed significantly in acute and non-acute phases of KD. Therefore, the authors believed that gut microbiota disorder is probably related to the pathogenesis of KD. However, the study is a self-controlled clinical trial without a healthy control group, so it cannot directly show the changes of gut microbiota composition in children with KD. In addition, the above study did not assess the diversity, abundance, and structural changes of gut microbiota. Therefore, in view of the above problems, we applied high-throughput sequencing technology to detect the gut microbiota of KD and healthy children in an attempt to provide a research basis for subsequent studies on the microecology of KD. The results showed that compared with the control group, the richness and diversity of gut microbiota in the KD group were significantly reduced, especially *Bacteroidetes*, and *Dorea* was almost absent in the KD group.

*Bacteroidetes* has a powerful polysaccharide degradation system that can absorb undigested polysaccharides and then metabolize them into short-chain fatty acids, thereby improving intestinal epithelial barrier function and downregulating the level of inflammatory cytokines (Glowacki et al., 2020). Hence, to a certain extent, *Bacteroides* can limit the intestinal invasion of pathogens and toxins related to KD. Meanwhile, it may alleviate the inflammatory reaction during the acute period of KD. *Dorea* is the main acrogenic bacterium in the human intestinal tract, which produces short-chain fatty acids and different amounts of hydrogen and carbon dioxide through carbohydrate fermentation (Rajilić-Stojanović et al., 2015; Taras et al., 2002). Hydrogen is a novel antioxidant that has aroused wide attention in recent years. A great number of studies have demonstrated that hydrogen may protect tissues via multiple mechanisms (Li et al., 2018; Meng et al., 2016; Nishimaki et al., 2018; Niu et al., 2020; Ohta, 2014). Of these mechanisms, the anti-inflammatory effect of hydrogen is noteworthy. Zhang et al. (2015) found that the inhalation of hydrogen can inhibit the systemic inflammatory response of septic mice, thereby reducing the pathological damage caused by inflammatory factors and enhancing the survival rate of septic mice. In 2012, a Japanese study used hydrogen to treat four feverish patients with acute cutaneous lupus erythematosus (Ono et al., 2012). The above research background demonstrates that an appropriate amount of hydrogen can strengthen intestinal endothelial barrier function and play an anti-inflammatory role. These findings suggest that hydrogen may be used to treat KD manifested as vasculitis and cardiac trauma.

To date, no highly specific laboratory index has been identified for KD, but blood test results of KD patients exhibit specific characteristics (Lee, Rhim & Kang, 2012). In the KD group of this study, C3, C4, WBC, ALT, and AST increased, whereas HGB and TP decreased. These findings were consistent with expectations. In most cases, thrombocytopenia occurs in the second week of KD, reaches its peak in the third week, and returns to normal 4–6 weeks after onset (Marchesi et al., 2018). The children with KD in this study were all in the acute phase (mostly in the first week of the disease). Their platelets exhibited no significant change compared with the control group, which is also consistent with the literature report.

Traditional viewpoints propose that the immune system is abnormally activated in the acute stage of KD. Blood vessels are damaged by a large number of cytokines and anti-endothelial cytotoxic antibodies (Andreozzi et al., 2017; Galeotti et al., 2016), and the level of immune globulin generally increases (Menikou, Langford & Levin, 2019). However, in this study, IgA and IgG in the peripheral blood of the KD group decreased, which may be related to the following factors. Although the activation of immune cells and various cytokines has been reported, some studies found that immune cell functions were also inhibited in KD patients (Kimura et al., 2004; Lee et al., 2015; Matsubara et al., 1999). This finding is consistent with our findings. The sum of IgA and IgG accounts for 90% of the total immunoglobulin in serum, which is the “main force” of anti-infection in the body. One study confirmed that microbiota can enrich naive B cell repertoires and immunoglobulin repertoires (Chen et al., 2018). The decrease in immunoglobulin may be related to the abnormal development and function of the immune system caused by gut microbiota disorder. It is hypothesized to be related to gut microbiota disorder in child patients, but the specific mechanism needs to be further investigated. In addition, since the inclusion criteria of the research objects were strictly formulated, all child patients complicated with common pathogen infection were excluded. However, KD patients included in traditional studies were often complicated with infections by mycoplasma, adenovirus, or other pathogens. Infection stimulates the enhancement of humoural immunity and the elevation of immunoglobulin levels (Ahmed, Ford & Sanz, 2019). These effects may explain the differences in immune globulin levels in peripheral blood.

Based on the above background, we propose the following hypothesis: genetic, environmental and other factors cause gut microbiota disorder in KD-susceptible children. Hence, the normal intestinal microecology cannot be established, and autoimmune tolerance is broken. In addition, immune cell subsets exhibit abnormal differentiation, immunoglobulin production is reduced, and the intestinal mucosal barrier function is damaged. Eventually, enterogenous invasion of intestinal microorganisms and their metabolites leads to KD. This hypothesis needs more in-depth mechanistic research.

## Conclusions

In summary, dysbiosis of gut microbiota occurs in children with acute KD. We hypothesize that the development of KD is associated with restricted intestinal microbiota diversity and an impaired gut barrier that leads to a number of different microbiota-associated immune dysregulatory events. In addition, this study is the first to report that the hydrogen-producing bacterium *Dorea* was significantly reduced in children with acute KD. This finding also further suggests that the existing treatment methods combined with probiotic intervention treatment are likely to be helpful for the treatment or prognosis of KD. As a preventive and therapeutic medical gas, molecular hydrogen can also be used innovatively in the adjuvant treatment of Kawasaki disease.

## Supplemental Information

10.7717/peerj.9698/supp-1 Supplemental Information 1 Sequences.

## Additional Information and Declarations

### Data Availability

The following information was supplied regarding data availability: The data is available at NCBI SRA: SRP257980.
