# Supplementary material for: Effects of changes on gut microbiota in children with acute Kawasaki disease
Source: PeerJ. 2020 Aug 6;8:e9698. doi: 10.7717/peerj.9698 (PMC7512135; doi:10.7717/peerj.9698)
Supplement: Supplemental Information 1 [file peerj-08-9698-s001.zip › B07_taxa_summary/taxa_summary_plots/charts/9SfHZD21CTCdwlg208KOL91EpHHqzh_legend.pdf]

- k__Bacteria;p__Firmicutes;c__Clostridia;o__Clostridiales
- k__Bacteria;p__Bacteroidetes;c__Bacteroidia;o__Bacteroidales
- k__Bacteria;p__Firmicutes;c__Bacilli;o__Lactobacillales
- k__Bacteria;p__Proteobacteria;c__Gammaproteobacteria;o__Enterobacteriales
- k__Bacteria;p__Actinobacteria;c__Actinobacteria;o__Bifidobacteriales
- k__Bacteria;p__Firmicutes;c__Erysipelotrichi;o__Erysipelotrichales
- k__Bacteria;p__Proteobacteria;c__Betaproteobacteria;o__Burkholderiales
- k__Bacteria;p__Proteobacteria;c__Gammaproteobacteria;o__Pseudomonadales
- k__Bacteria;p__Actinobacteria;c__Actinobacteria;o__Actinomycetales
- k__Bacteria;p__Actinobacteria;c__Coriobacteriia;o__Coriobacteriales
- k__Bacteria;p__Proteobacteria;c__Deltaproteobacteria;o__Desulfovibrionales
- k__Bacteria;p__Proteobacteria;c__Gammaproteobacteria;o__Xanthomonadales
- k__Bacteria;p__TM7;c__TM7-3;o__Unclassified_TM7-3
- k__Bacteria;p__Proteobacteria;c__Alphaproteobacteria;o__Rhizobiales
- k__Bacteria;p__Cyanobacteria;c__Chloroplast;o__Streptophyta
- k__Bacteria;p__Proteobacteria;c__Alphaproteobacteria;o__Caulobacterales
- k__Bacteria;p__Firmicutes;c__Bacilli;o__Bacillales
- k__Bacteria;p__Proteobacteria;c__Gammaproteobacteria;o__Pasteurellales
- k__Bacteria;p__Proteobacteria;c__Gammaproteobacteria;o__Alteromonadales
- k__Bacteria;p__Proteobacteria;c__Alphaproteobacteria;o__Sphingomonadales
- k__Bacteria;p__[Thermi];c__Deinococci;o__Thermales
- k__Bacteria;p__Proteobacteria;c__Gammaproteobacteria;o__Aeromonadales
- k__Bacteria;p__Proteobacteria;c__Alphaproteobacteria;o__RF32
- k__Bacteria;p__Actinobacteria;c__Acidimicrobiia;o__Acidimicrobiales
- k__Bacteria;p__Bacteroidetes;c__[Saprospirae];o__[Saprospirales]
- k__Bacteria;p__Acidobacteria;c__iii1-8;o__SJA-36
- k__Bacteria;p__Proteobacteria;c__Epsilonproteobacteria;o__Campylobacterales
- k__Bacteria;p__Acidobacteria;c__Acidobacteria-6;o__iii1-15
- k__Bacteria;p__Proteobacteria;c__Betaproteobacteria;o__Rhodocyclales
- k__Bacteria;p__Firmicutes;c__Bacilli;o__Gemellales
- k__Bacteria;p__Bacteroidetes;c__Sphingobacteriia;o__Sphingobacteriales
- k__Bacteria;p__Fusobacteria;c__Fusobacteriia;o__Fusobacteriales
- k__Bacteria;p__Proteobacteria;c__Alphaproteobacteria;o__Rhodobacterales
- k__Bacteria;p__Verrucomicrobia;c__Verrucomicrobiae;o__Verrucomicrobiales
- k__Bacteria;p__Gemmatimonadetes;c__Gemm-1;o__Unclassified_Gemm-1
- k__Bacteria;p__Proteobacteria;c__Deltaproteobacteria;o__Syntrophobacterales
- k__Bacteria;p__Cyanobacteria;c__Chloroplast;o__Stramenopiles
- k__Bacteria;p__Cyanobacteria;c__4C0d-2;o__MLE1-12
