# Supplementary material for: Effects of changes on gut microbiota in children with acute Kawasaki disease
Source: PeerJ. 2020 Aug 6;8:e9698. doi: 10.7717/peerj.9698 (PMC7512135; doi:10.7717/peerj.9698)
Supplement: Supplemental Information 1 [file peerj-08-9698-s001.zip › B07_taxa_summary_group/taxa_summary_plots/charts/7LKXY0N7DEuZA01fYeYcDPs1Jd1PEj_legend.pdf]

k__Bacteria;p__Firmicutes;c__Clostridia
k__Bacteria;p__Bacteroidetes;c__Bacteroidia
k__Bacteria;p__Proteobacteria;c__Gammaproteobacteria
k__Bacteria;p__Firmicutes;c__Bacilli
k__Bacteria;p__Actinobacteria;c__Actinobacteria
k__Bacteria;p__Firmicutes;c__Erysipelotrichi
k__Bacteria;p__Proteobacteria;c__Betaproteobacteria
k__Bacteria;p__Actinobacteria;c__Coriobacteriia
k__Bacteria;p__Proteobacteria;c__Deltaproteobacteria
k__Bacteria;p__Proteobacteria;c__Alphaproteobacteria
k__Bacteria;p__Cyanobacteria;c__Chloroplast
k__Bacteria;p__TM7;c__TM7-3
k__Bacteria;p__[Thermi];c__Deinococci
k__Bacteria;p__Actinobacteria;c__Acidimicrobiia
k__Bacteria;p__Bacteroidetes;c__[Saprospirae]
k__Bacteria;p__Acidobacteria;c__iii1-8
k__Bacteria;p__Acidobacteria;c__Acidobacteria-6
k__Bacteria;p__Proteobacteria;c__Epsilonproteobacteria
k__Bacteria;p__Bacteroidetes;c__Sphingobacteriia
k__Bacteria;p__Verrucomicrobia;c__Verrucomicrobiae
k__Bacteria;p__Fusobacteria;c__Fusobacteriia
k__Bacteria;p__Gemmatimonadetes;c__Gemm-1
k__Bacteria;p__Cyanobacteria;c__4C0d-2
