# Supplementary material for: Effects of changes on gut microbiota in children with acute Kawasaki disease
Source: PeerJ. 2020 Aug 6;8:e9698. doi: 10.7717/peerj.9698 (PMC7512135; doi:10.7717/peerj.9698)
Supplement: Supplemental Information 1 [file peerj-08-9698-s001.zip › B07_taxa_summary_group/taxa_summary_plots/charts/i7SqO1gXp6rkH4OAADz75SjZya2E60_legend.pdf]

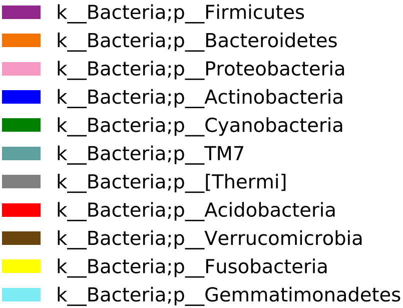
k__Bacteria;p__Firmicutes
k__Bacteria;p__Bacteroidetes
k__Bacteria;p__Proteobacteria
k__Bacteria;p__Actinobacteria
k__Bacteria;p__Cyanobacteria
k__Bacteria;p__TM7
k__Bacteria;p__[Thermi]
k__Bacteria;p__Acidobacteria
k__Bacteria;p__Verrucomicrobia
k__Bacteria;p__Fusobacteria
k__Bacteria;p__Gemmatimonadetes
