# Supplementary figures and images for: Effects of changes on gut microbiota in children with acute Kawasaki disease
Source: PeerJ. 2020 Aug 6;8:e9698. doi: 10.7717/peerj.9698 (PMC7512135; doi:10.7717/peerj.9698)

Length Distribution

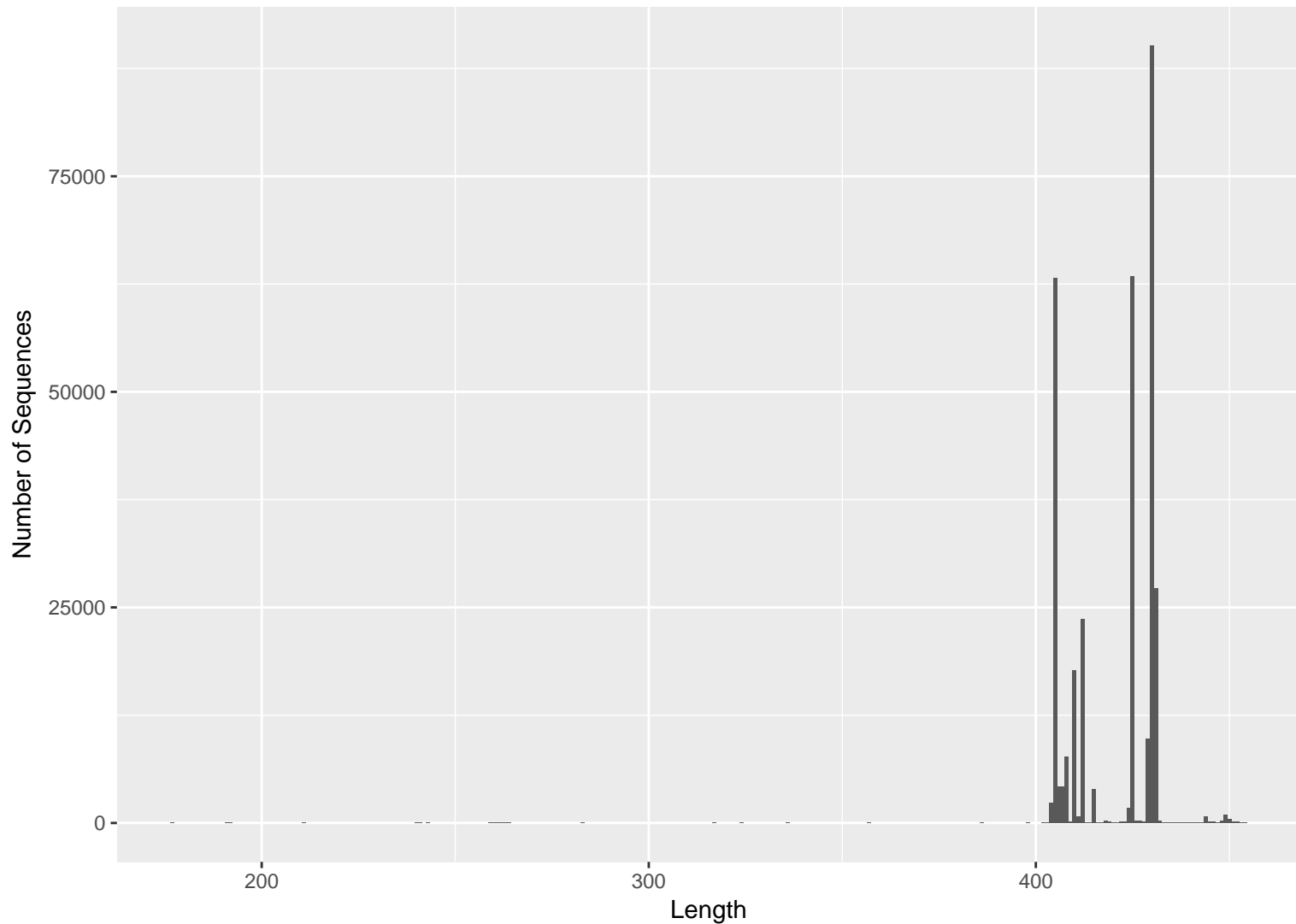

Supplement: Supplemental Information 1 [file peerj-08-9698-s001.zip › A_sequences/Length_Distribution.pdf]

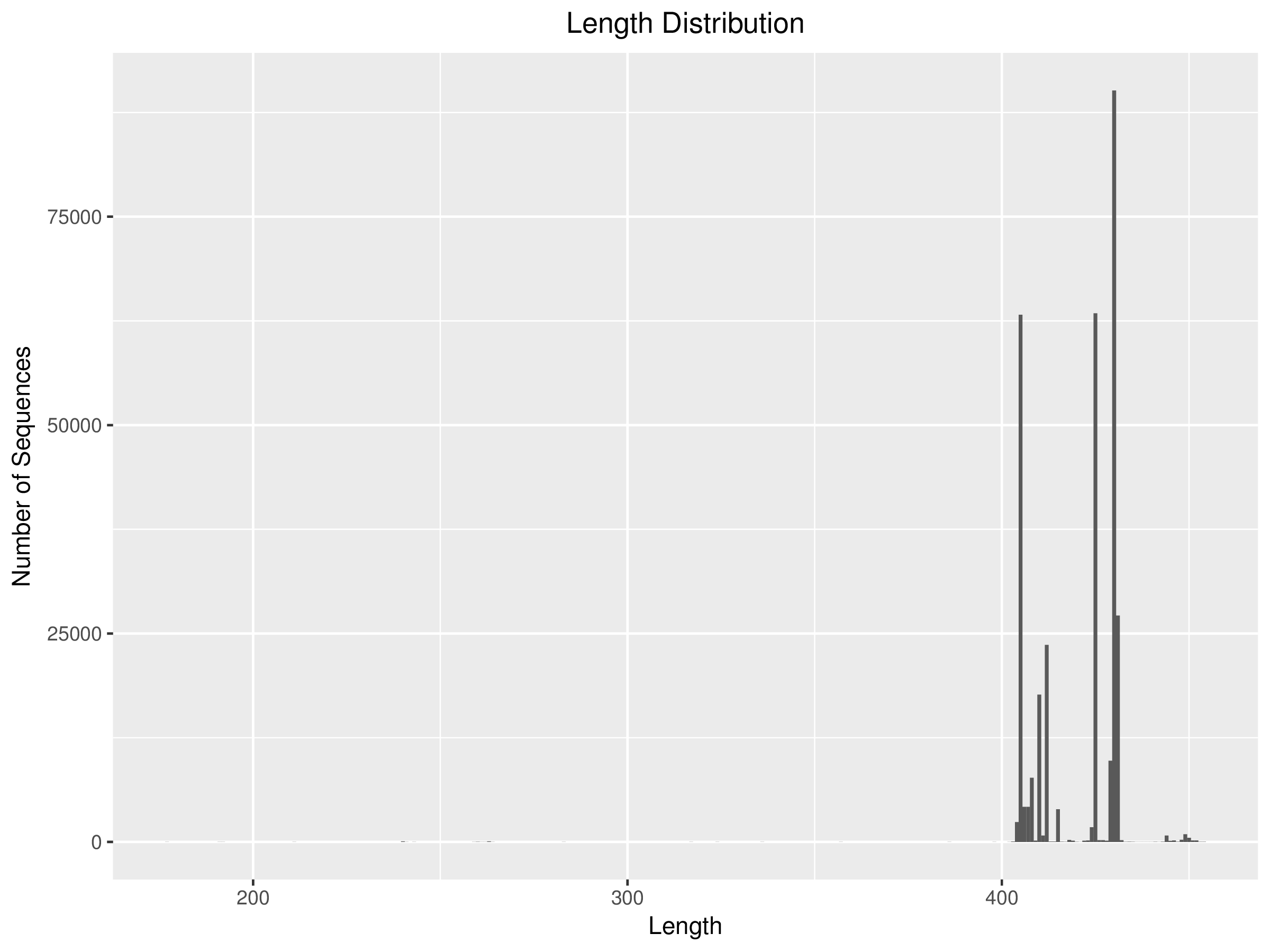

Supplement: Supplemental Information 1 [file peerj-08-9698-s001.zip › A_sequences/length_distribution.png]

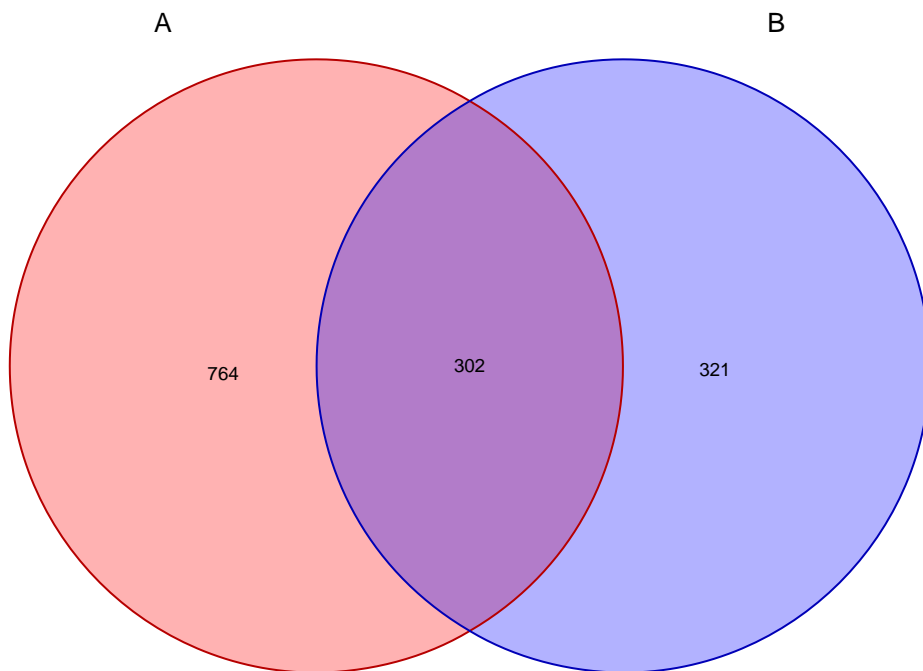

Supplement: Supplemental Information 1 [file peerj-08-9698-s001.zip › B01_OTU/venn/venn.A-B.pdf]

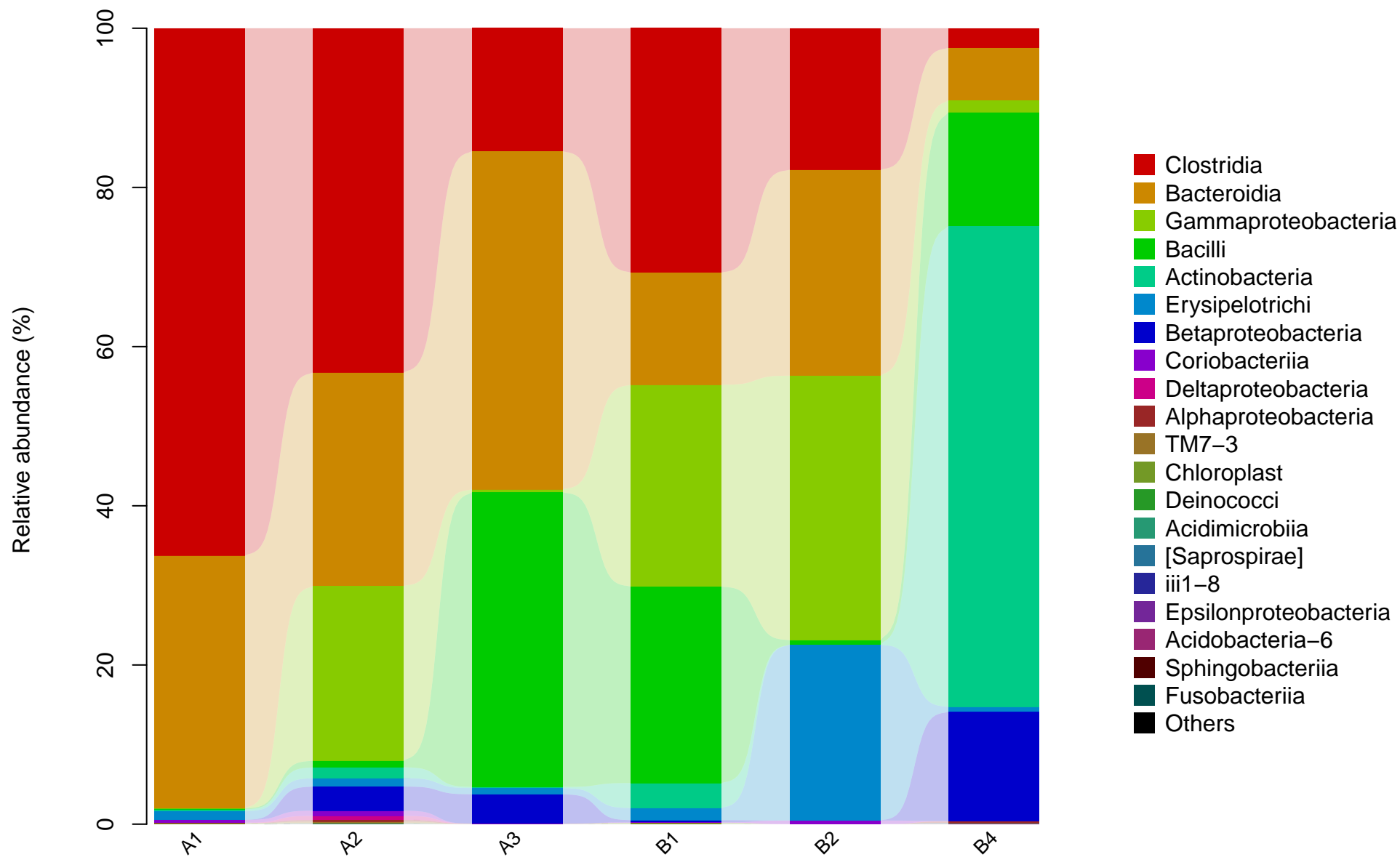

Supplement: Supplemental Information 1 [file peerj-08-9698-s001.zip › B07_taxa_summary/bar_class.pdf]

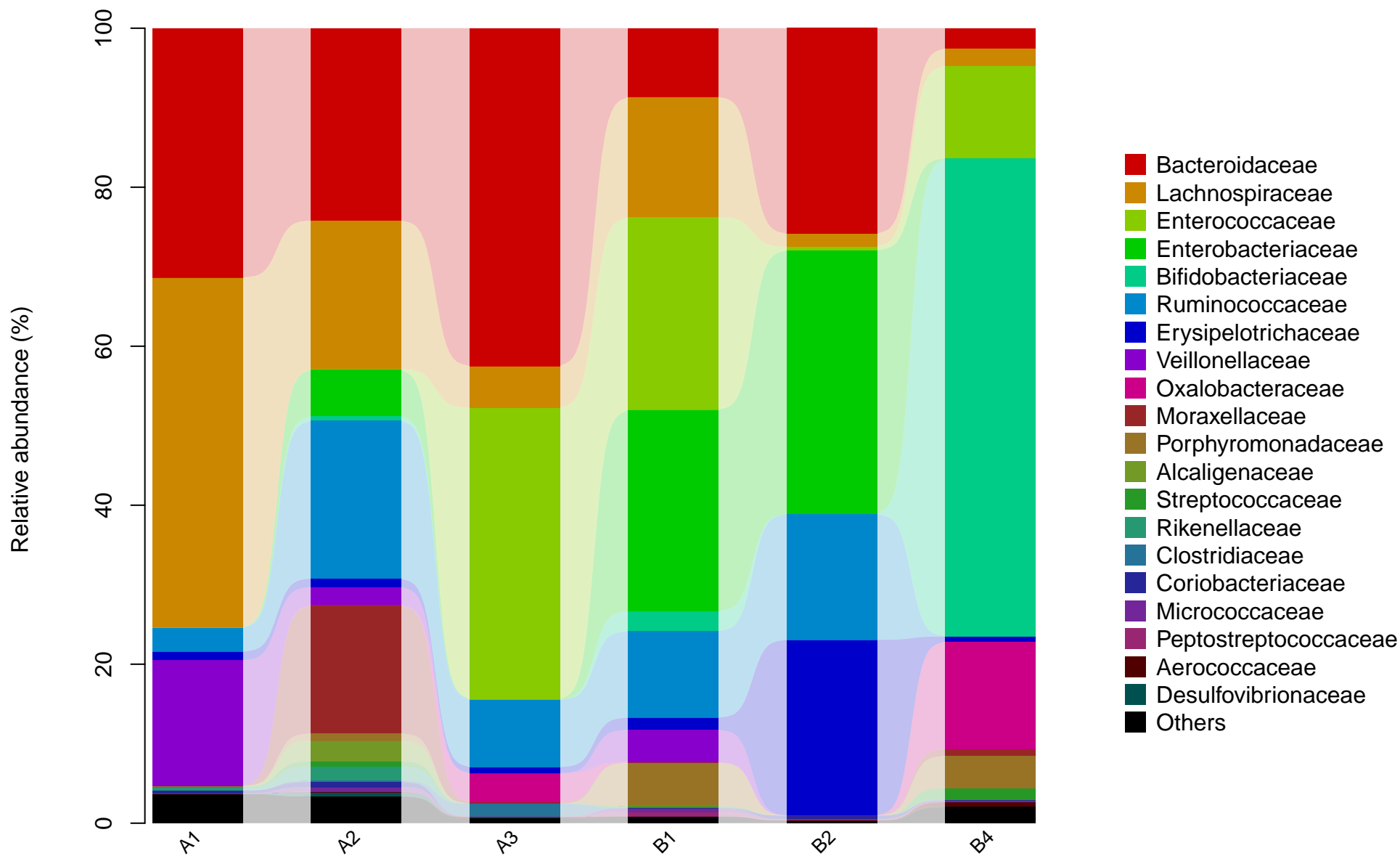

Supplement: Supplemental Information 1 [file peerj-08-9698-s001.zip › B07_taxa_summary/bar_family.pdf]

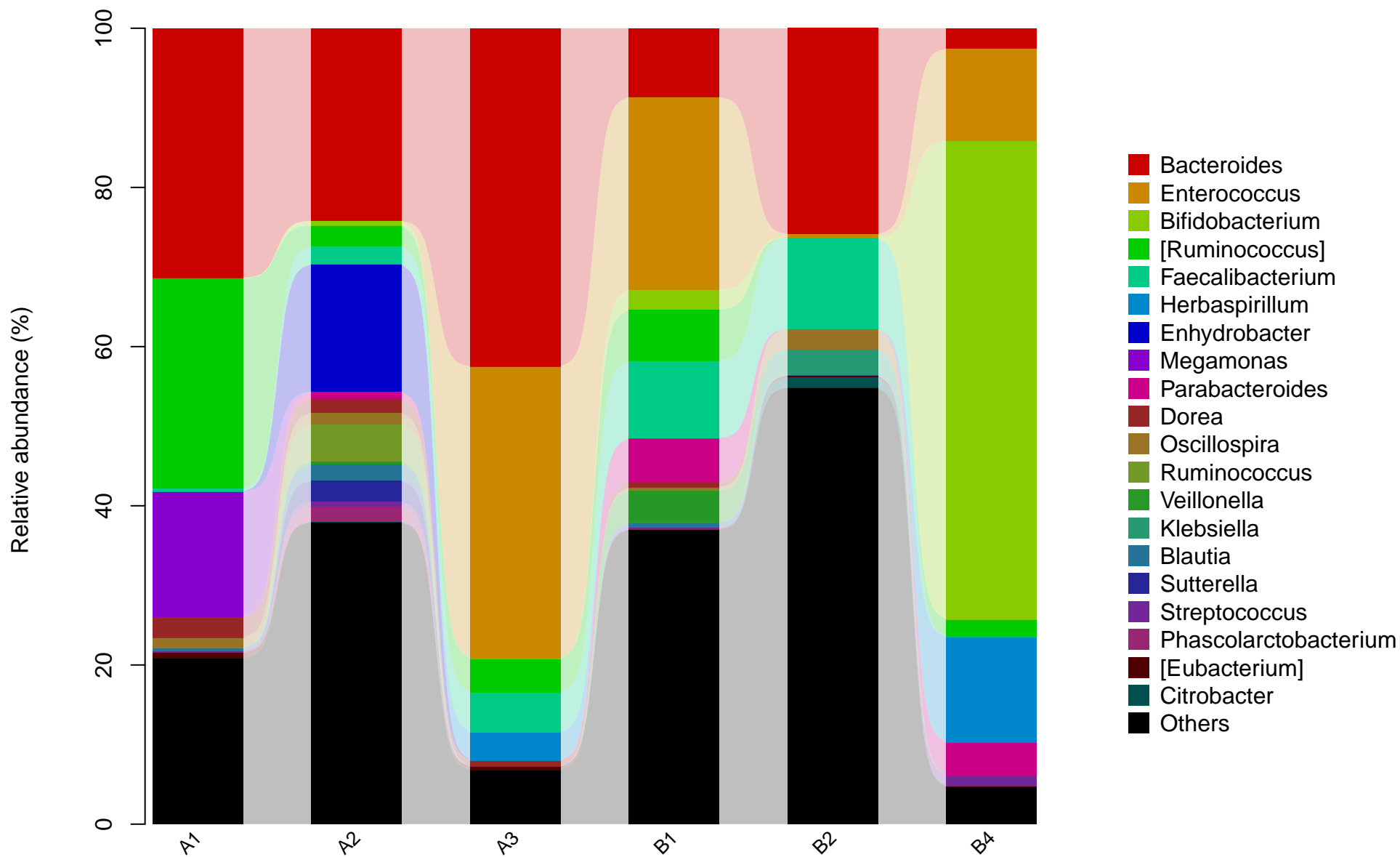

Supplement: Supplemental Information 1 [file peerj-08-9698-s001.zip › B07_taxa_summary/bar_genus.pdf]

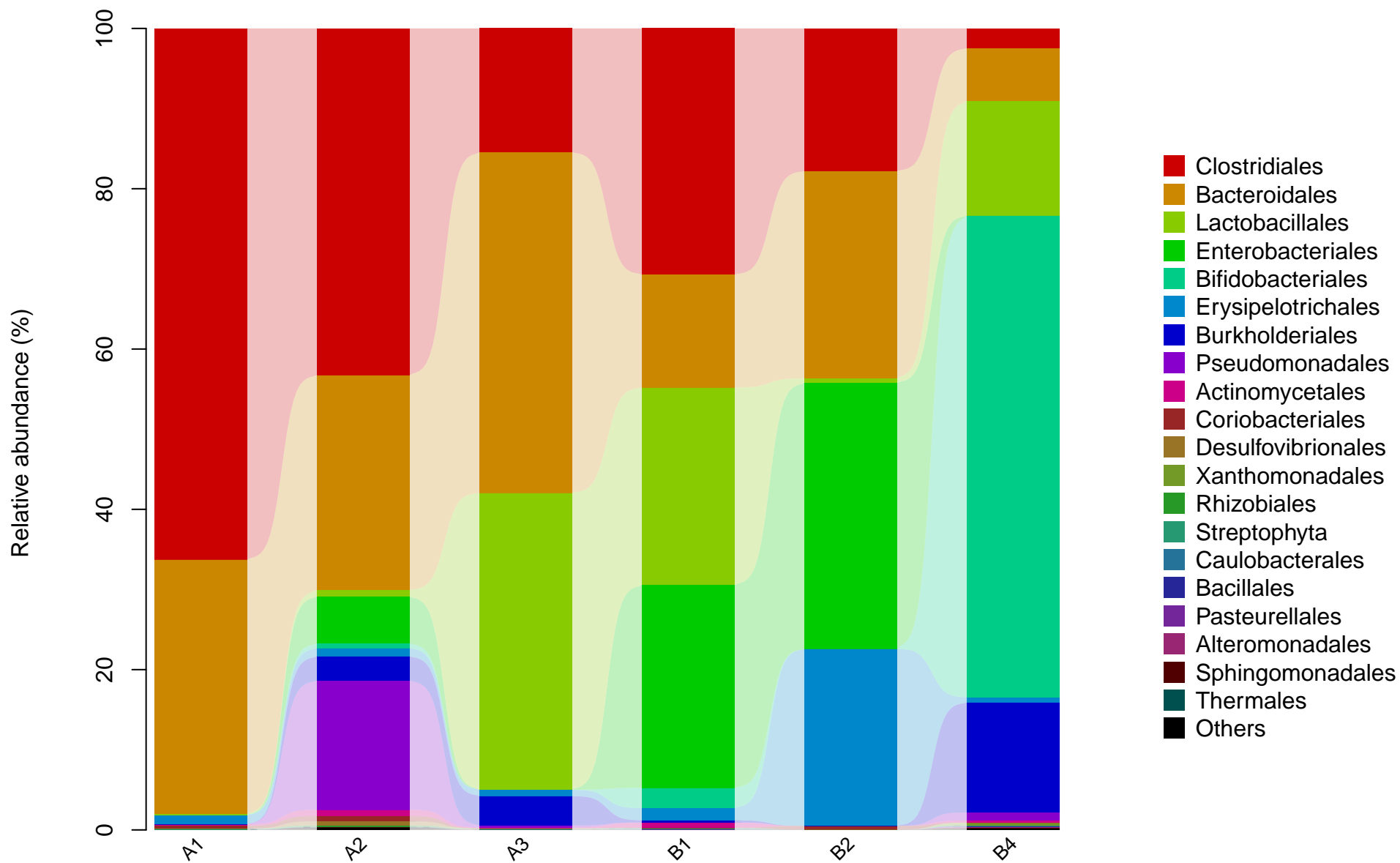

Supplement: Supplemental Information 1 [file peerj-08-9698-s001.zip › B07_taxa_summary/bar_order.pdf]

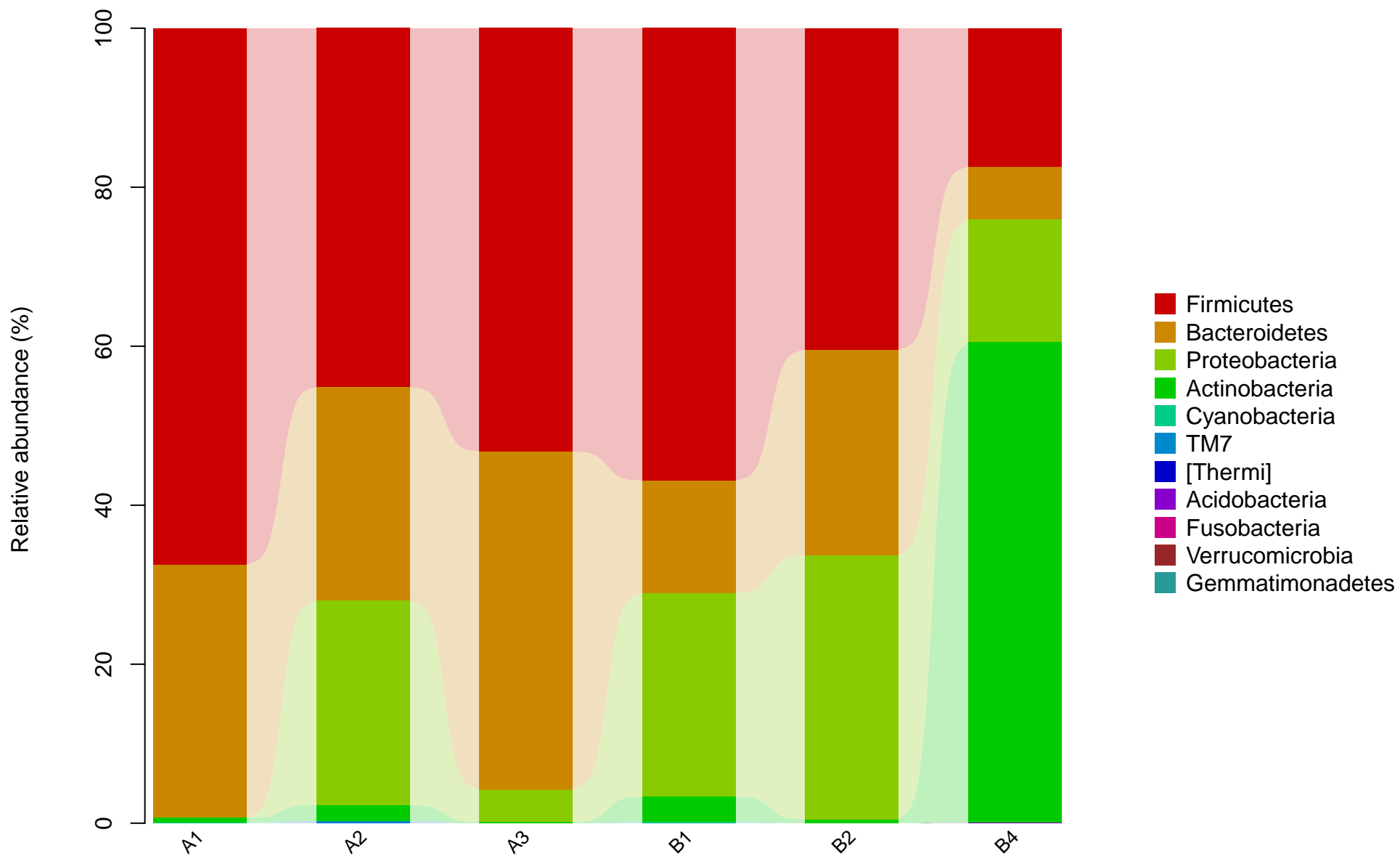

Supplement: Supplemental Information 1 [file peerj-08-9698-s001.zip › B07_taxa_summary/bar_phylum.pdf]

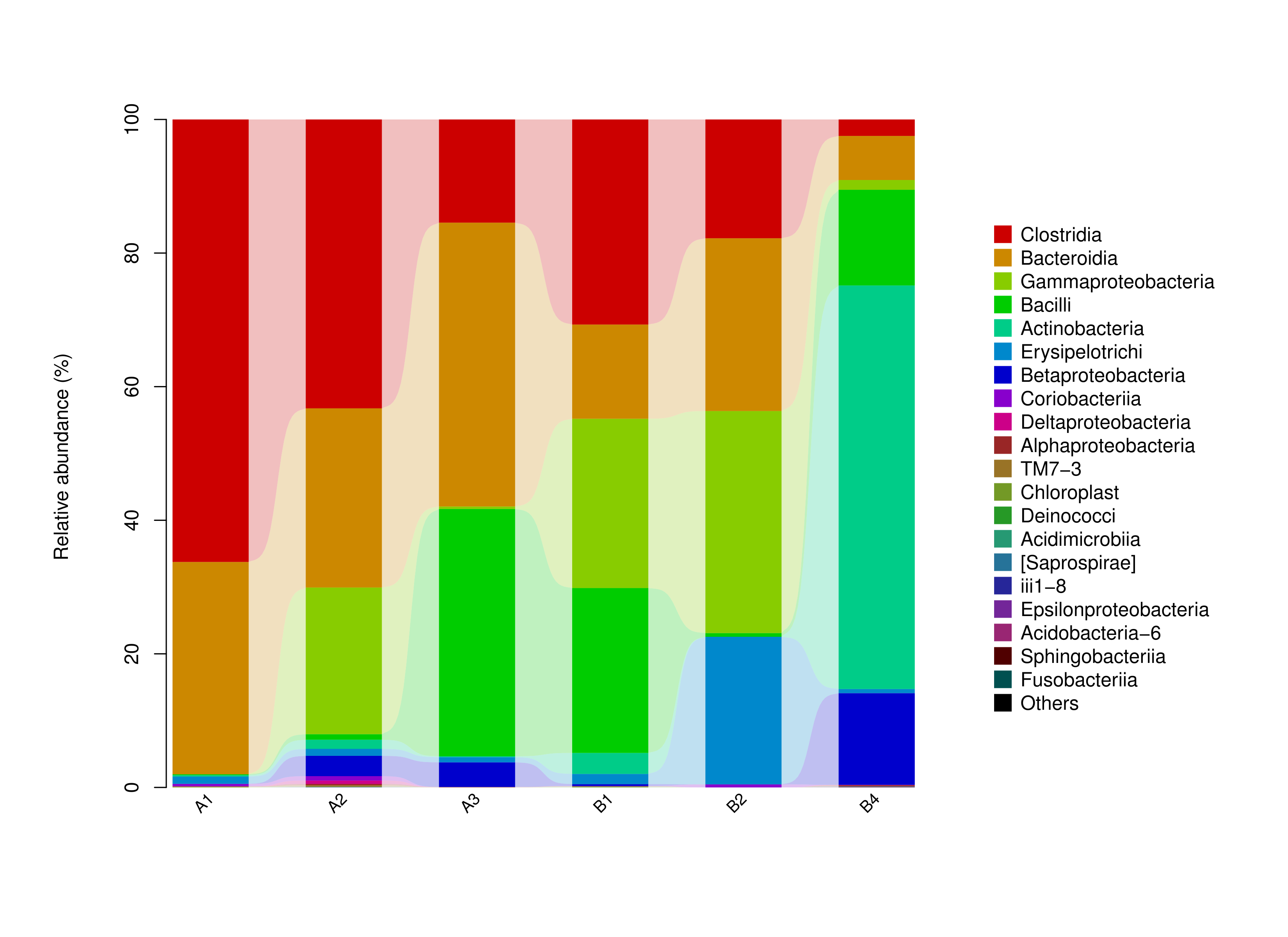

Supplement: Supplemental Information 1 [file peerj-08-9698-s001.zip › B07_taxa_summary/class.png]

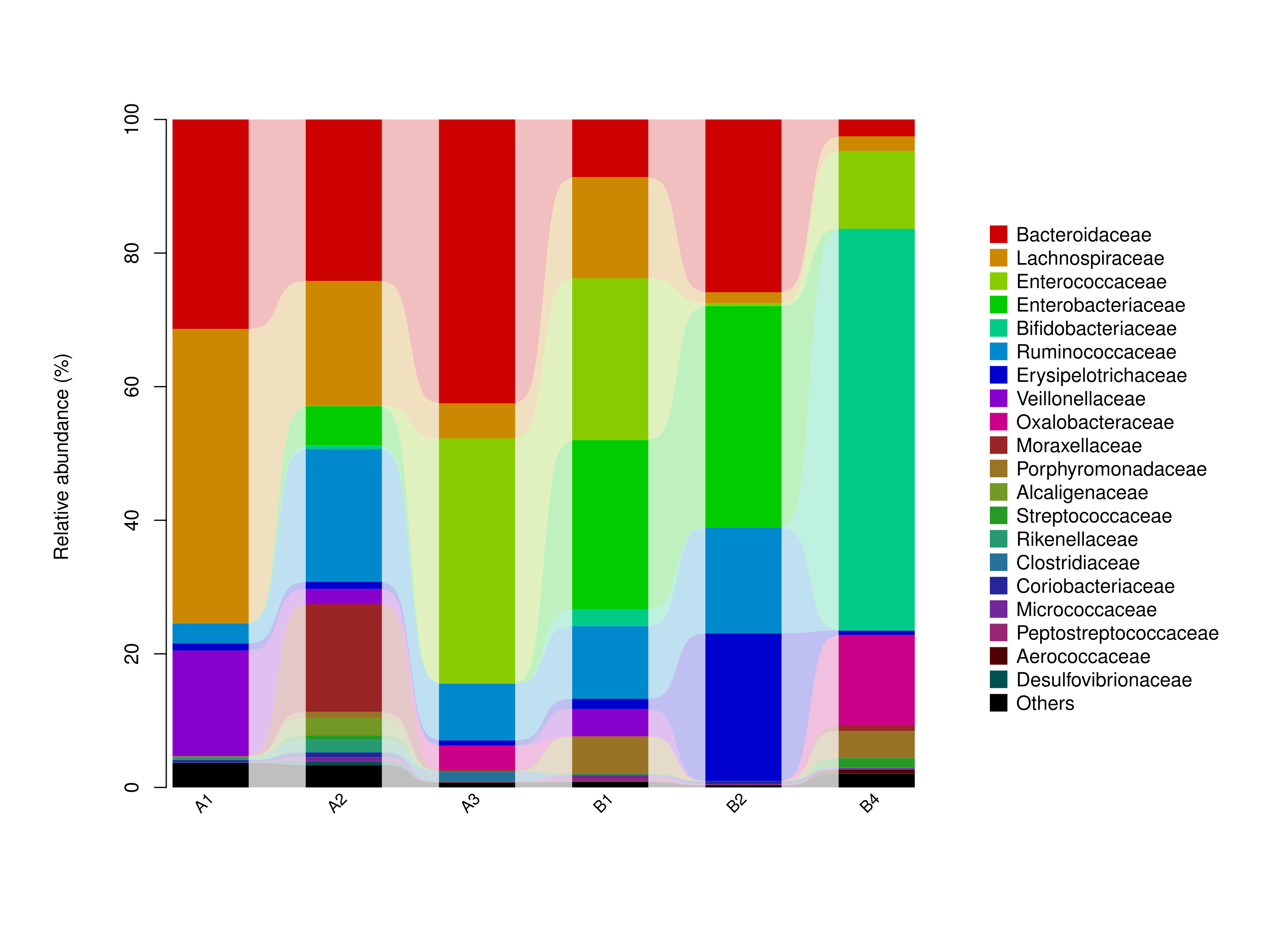

Supplement: Supplemental Information 1 [file peerj-08-9698-s001.zip › B07_taxa_summary/family.png]

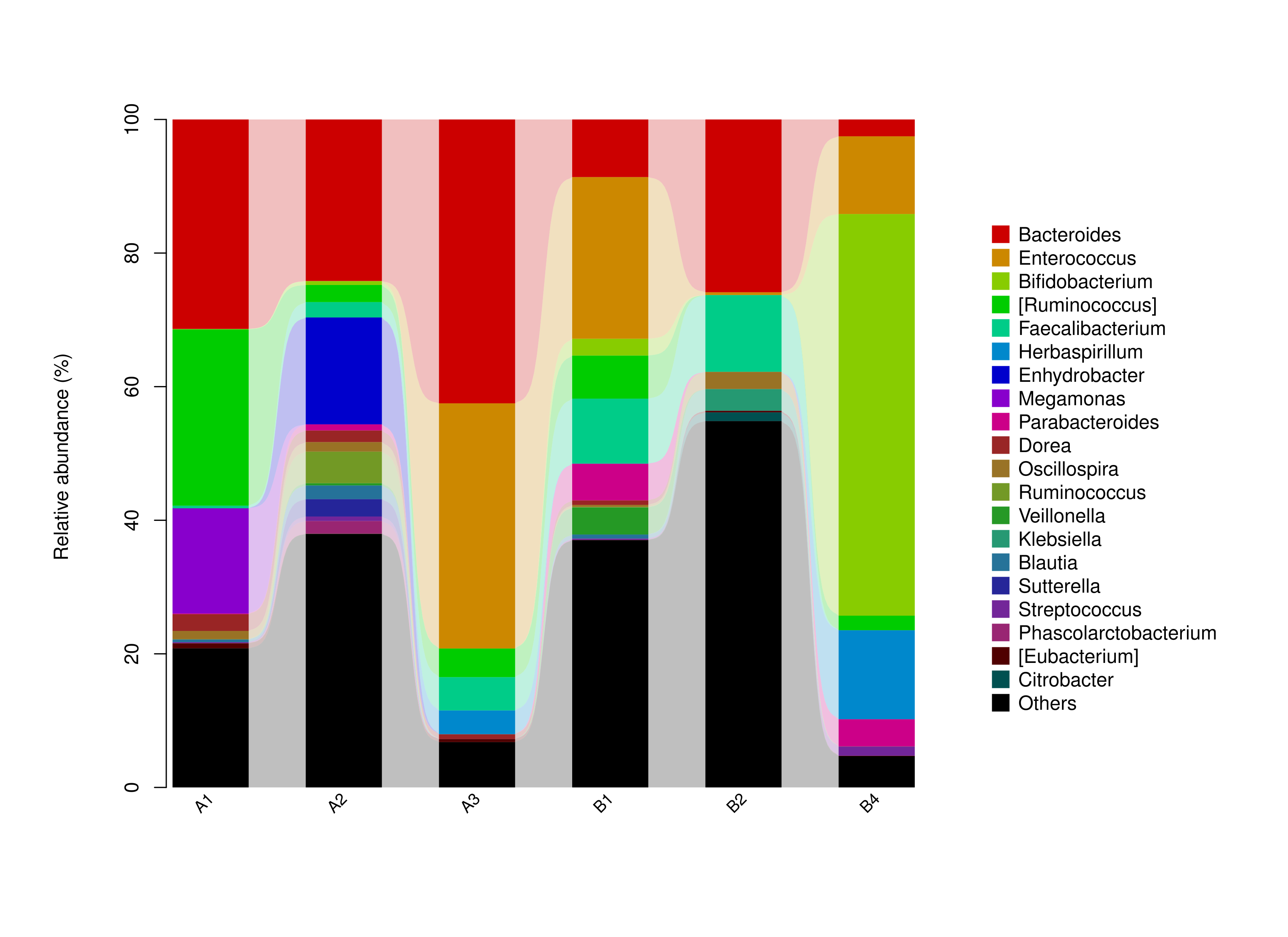

Supplement: Supplemental Information 1 [file peerj-08-9698-s001.zip › B07_taxa_summary/genus.png]

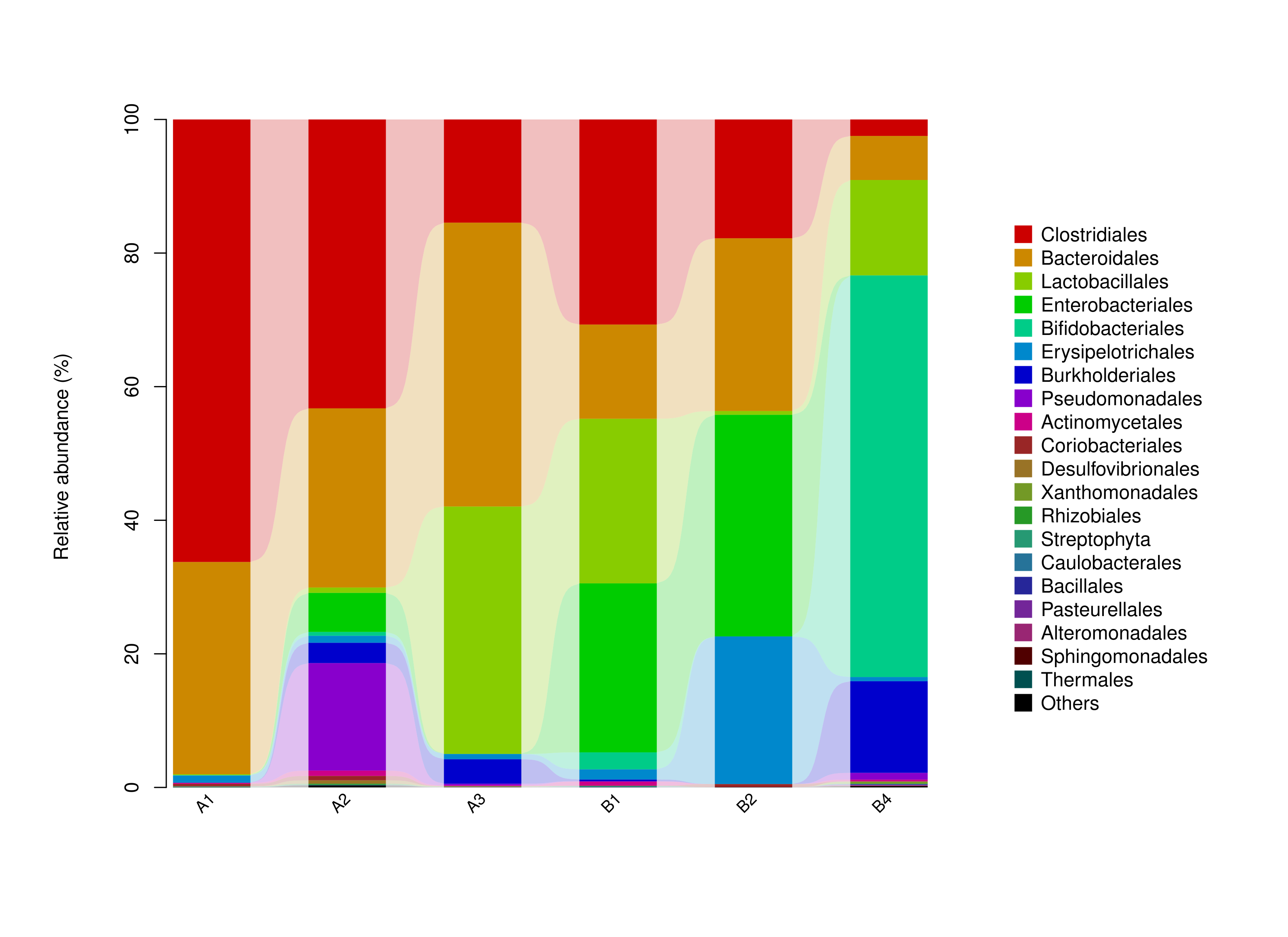

Supplement: Supplemental Information 1 [file peerj-08-9698-s001.zip › B07_taxa_summary/order.png]

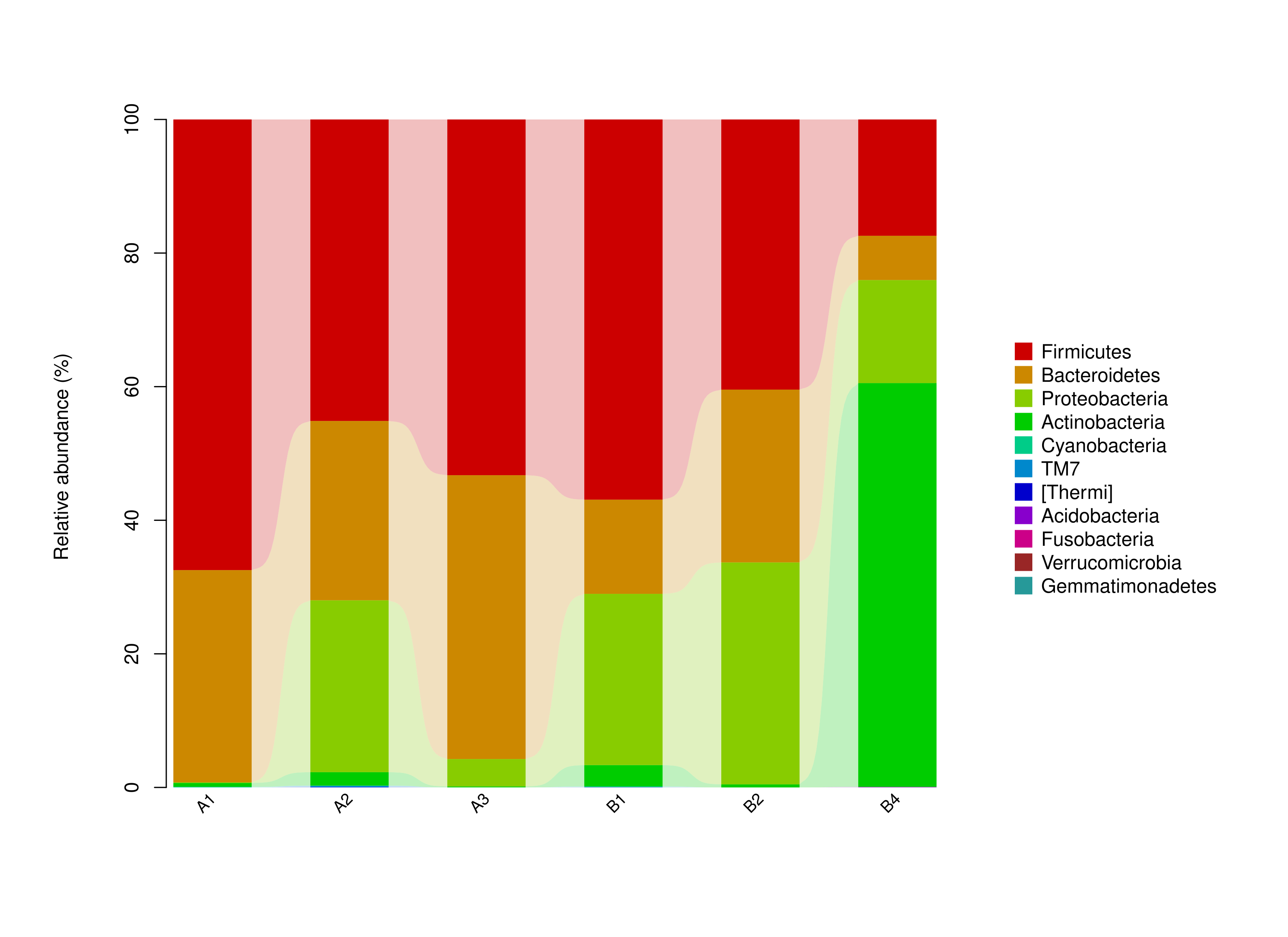

Supplement: Supplemental Information 1 [file peerj-08-9698-s001.zip › B07_taxa_summary/phylum.png]

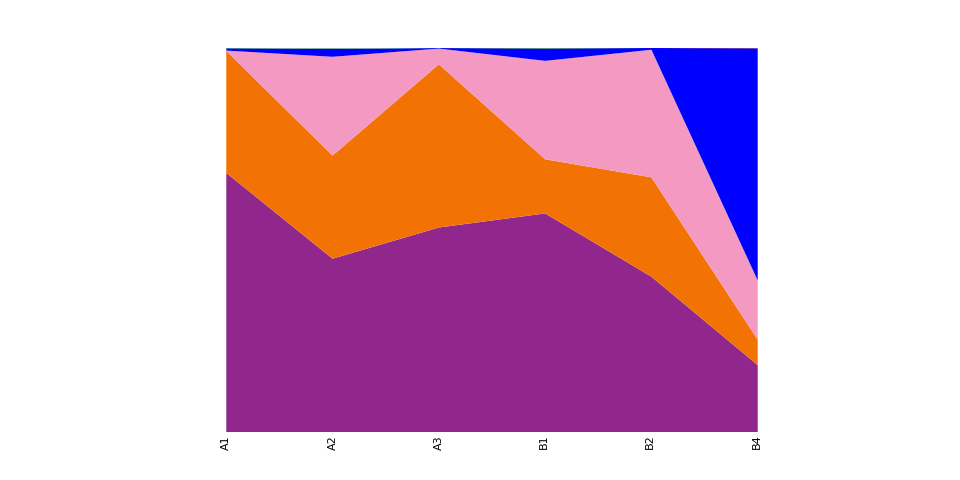

Supplement: Supplemental Information 1 [file peerj-08-9698-s001.zip › B07_taxa_summary/taxa_summary_plots/charts/2Ld3ZTXKOeAbOZpKEfjX325yq64Eeg.png]

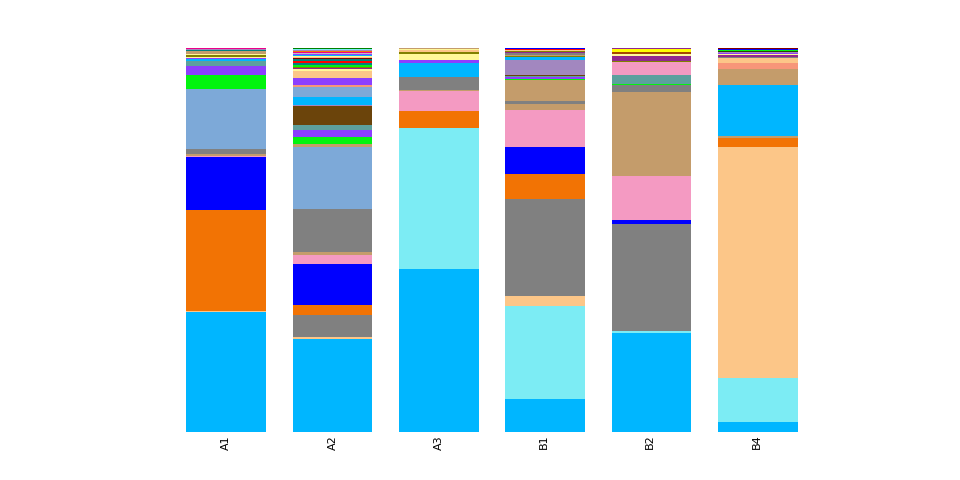

Supplement: Supplemental Information 1 [file peerj-08-9698-s001.zip › B07_taxa_summary/taxa_summary_plots/charts/2xIs2bZlZq3n5GRdrxD9Ang19b2C8z.png]

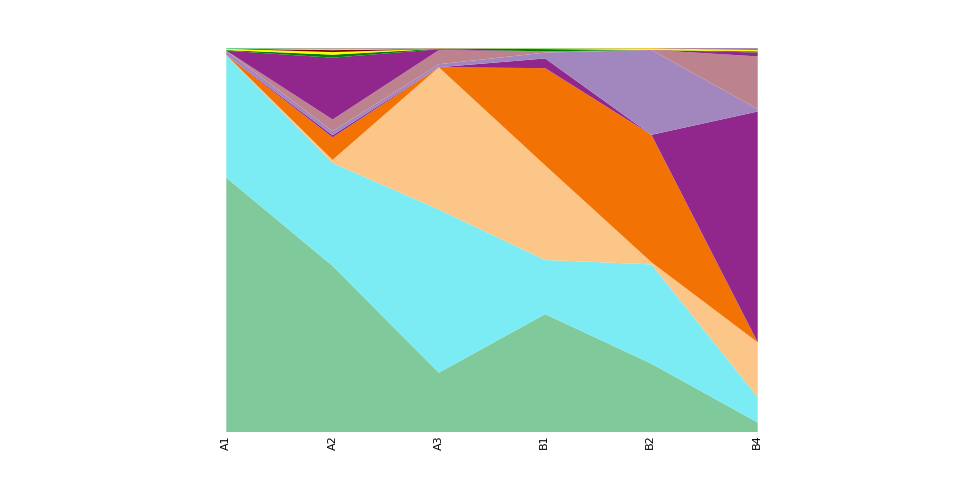

Supplement: Supplemental Information 1 [file peerj-08-9698-s001.zip › B07_taxa_summary/taxa_summary_plots/charts/9SfHZD21CTCdwlg208KOL91EpHHqzh.png]

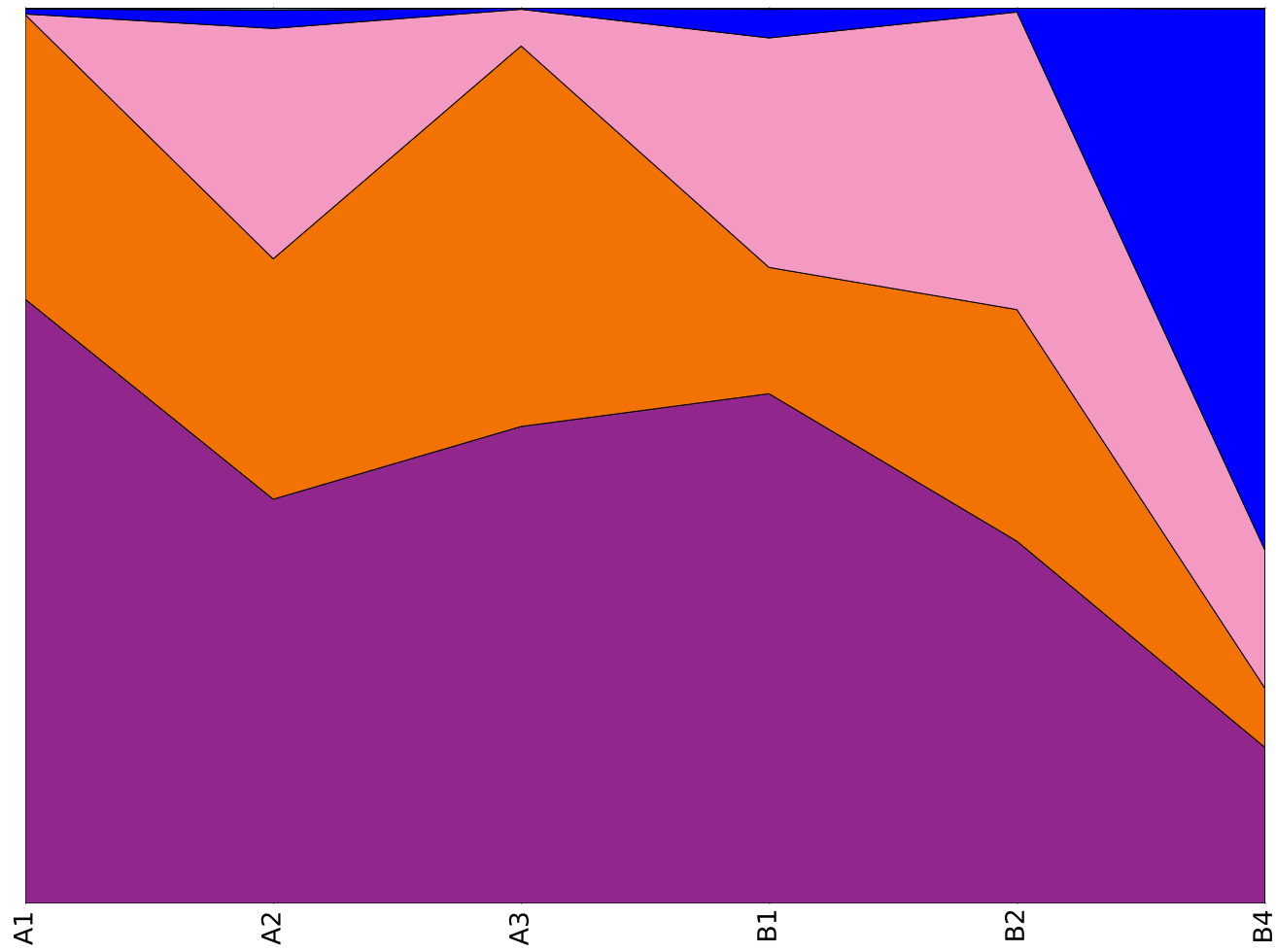

Supplement: Supplemental Information 1 [file peerj-08-9698-s001.zip › B07_taxa_summary/taxa_summary_plots/charts/Az3s0A1NsjUQ6XIqbxtqbGzp2S37PX.pdf]

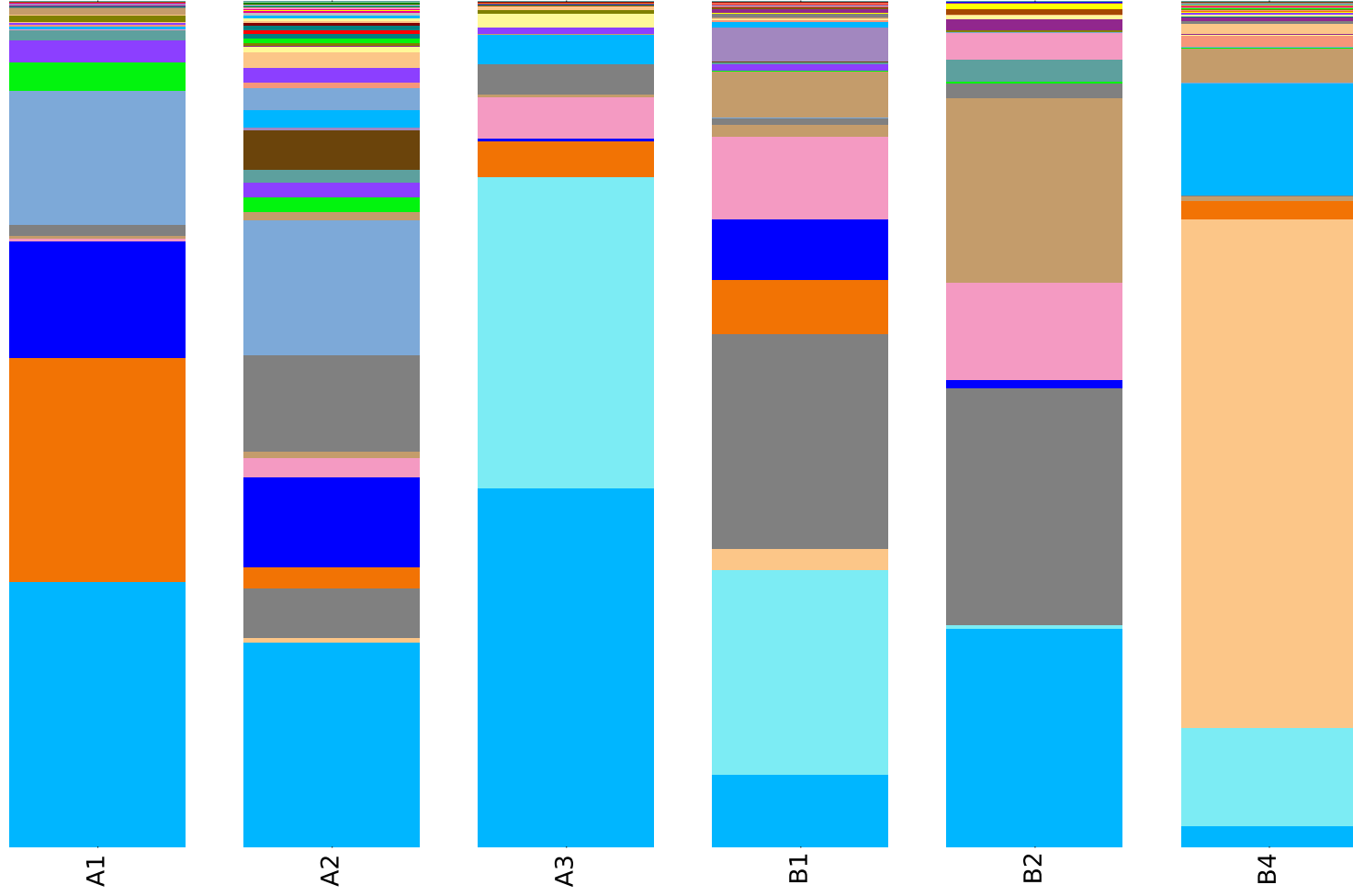

Supplement: Supplemental Information 1 [file peerj-08-9698-s001.zip › B07_taxa_summary/taxa_summary_plots/charts/BYJRQbGjkZhqMrjwYaPPxnbpdTYgUS.pdf]

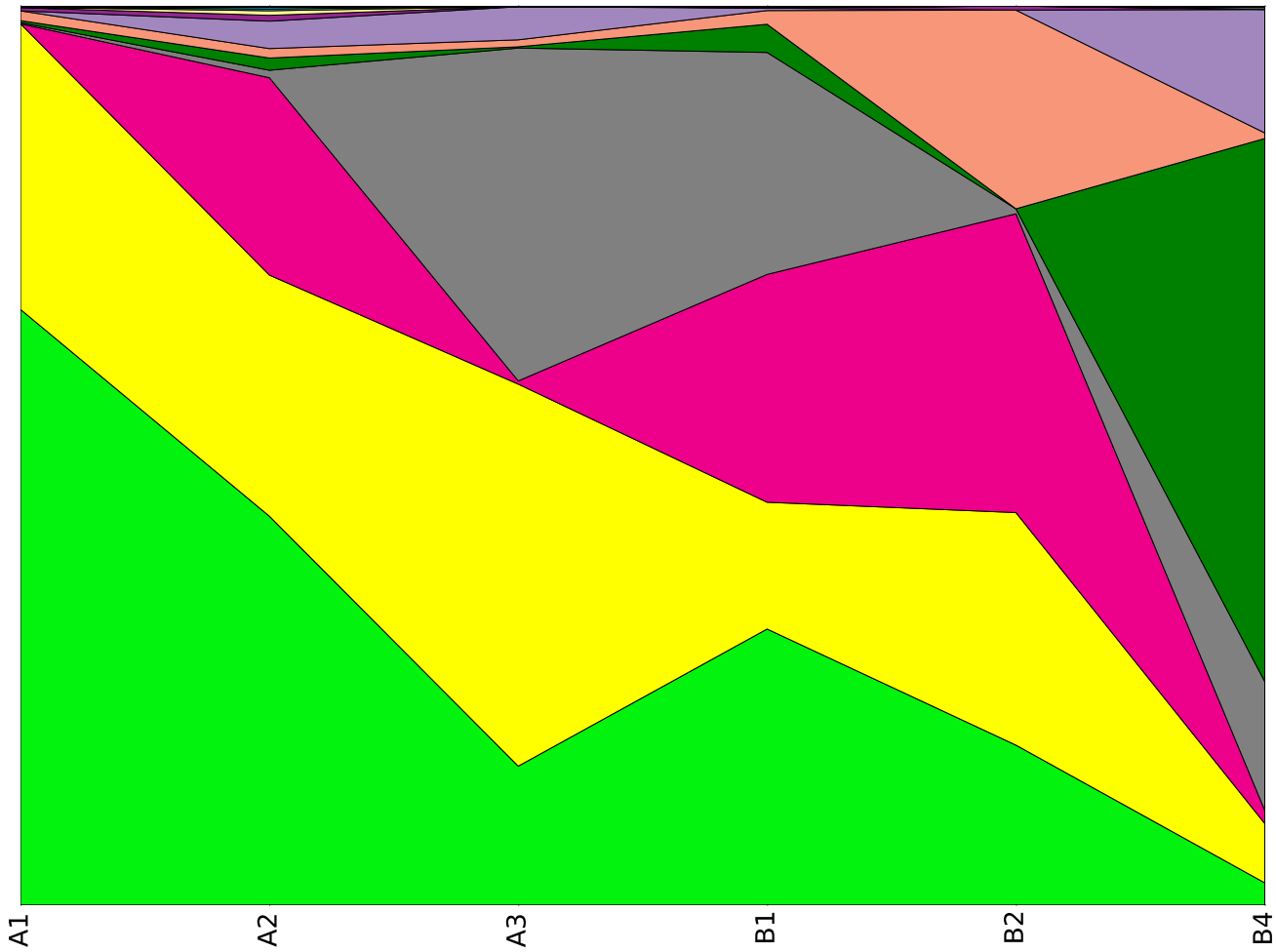

Supplement: Supplemental Information 1 [file peerj-08-9698-s001.zip › B07_taxa_summary/taxa_summary_plots/charts/cilImOHzxkSpYRixZ70pB3fcJwonNw.pdf]

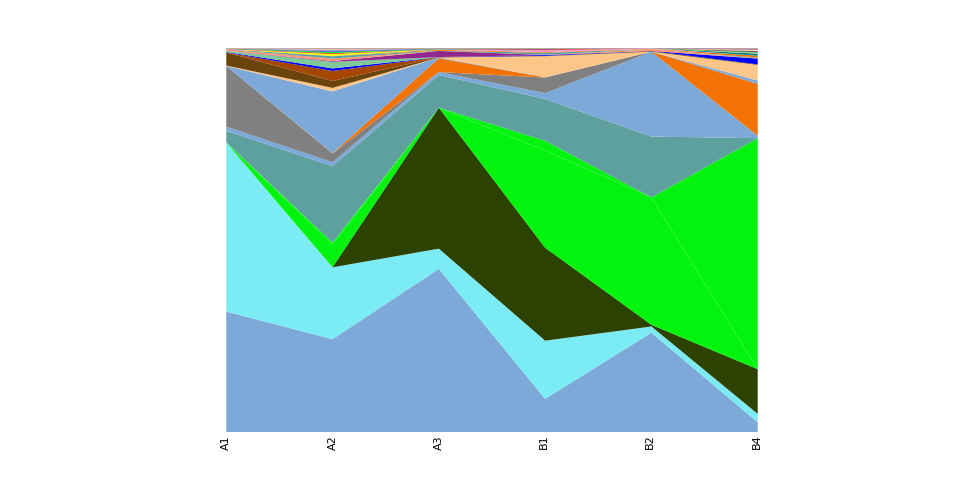

Supplement: Supplemental Information 1 [file peerj-08-9698-s001.zip › B07_taxa_summary/taxa_summary_plots/charts/dKnKqMGRUUqKIqW0Ezr9OERoHflLZf.png]

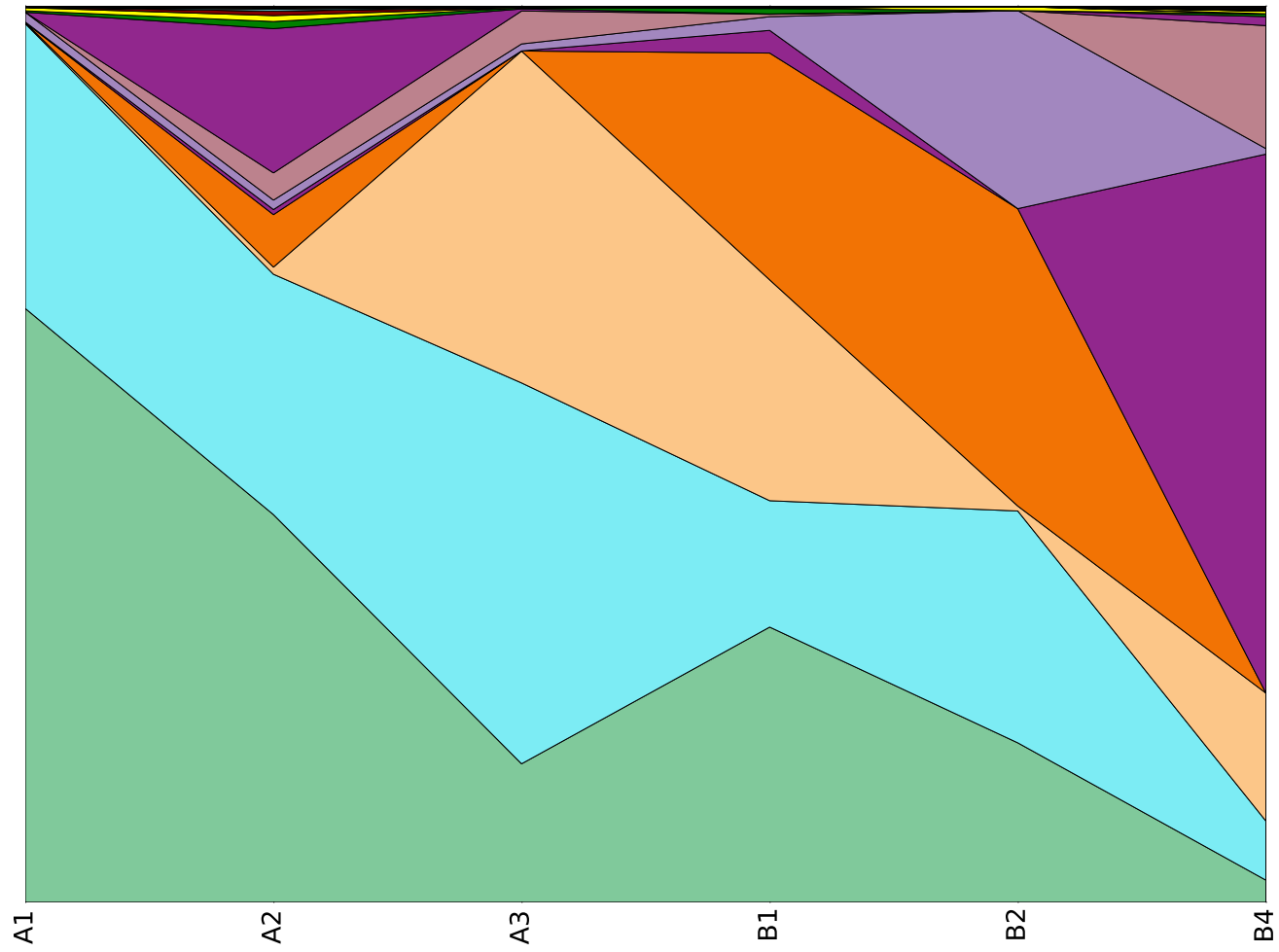

Supplement: Supplemental Information 1 [file peerj-08-9698-s001.zip › B07_taxa_summary/taxa_summary_plots/charts/Dzgl82smXIFDdwoMiu5Dq0TgLgW900.pdf]

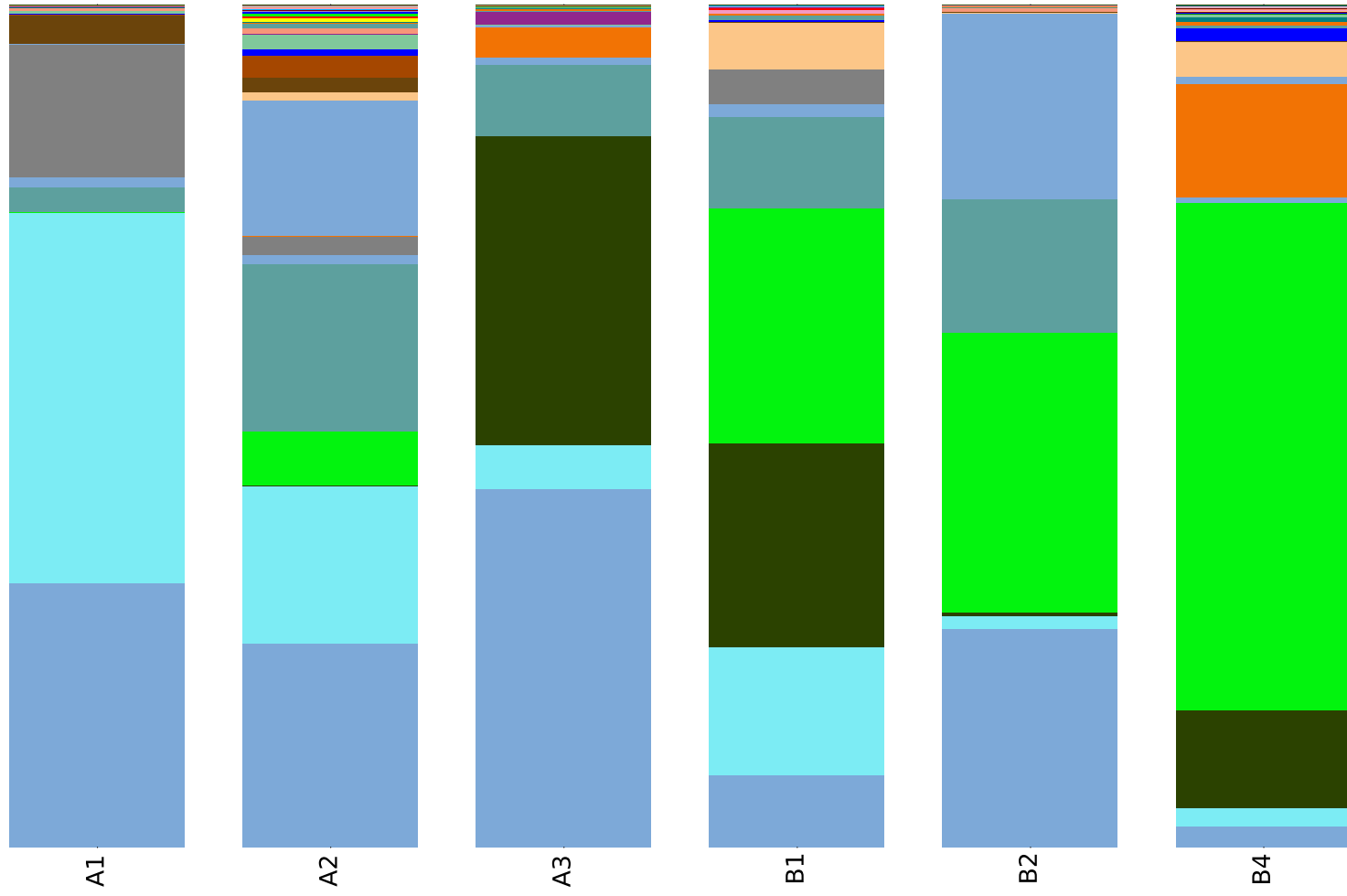

Supplement: Supplemental Information 1 [file peerj-08-9698-s001.zip › B07_taxa_summary/taxa_summary_plots/charts/e8kPRMU5XedhQwLwNJD1s5h9Qyxikc.pdf]

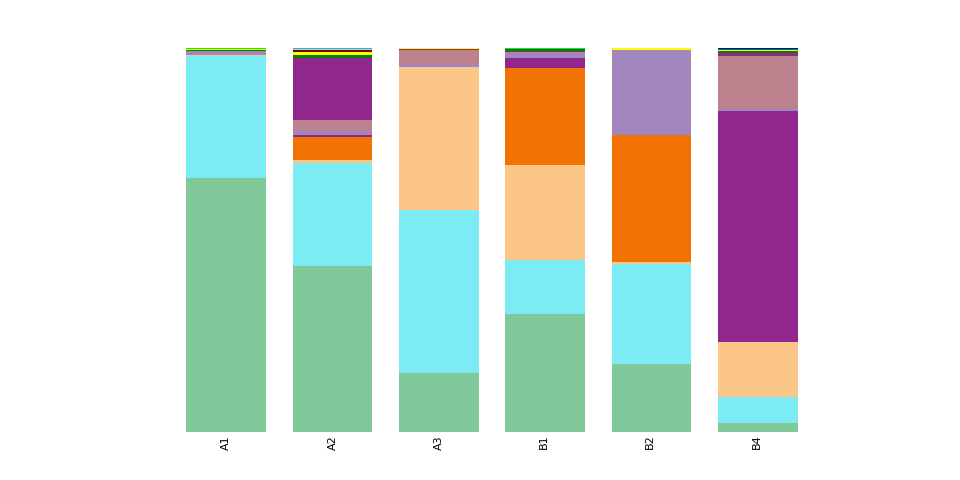

Supplement: Supplemental Information 1 [file peerj-08-9698-s001.zip › B07_taxa_summary/taxa_summary_plots/charts/g8gANXRUteoQyLyAlU0pUCfhPBIlDJ.png]

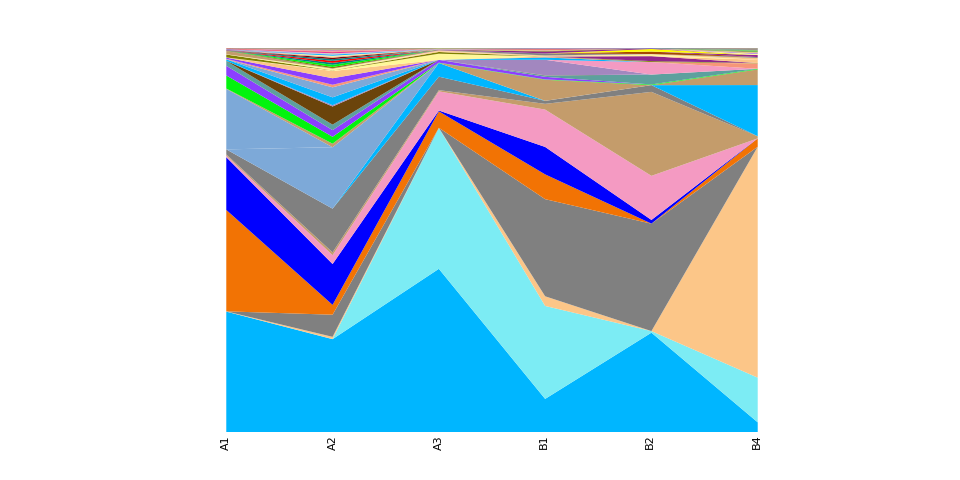

Supplement: Supplemental Information 1 [file peerj-08-9698-s001.zip › B07_taxa_summary/taxa_summary_plots/charts/gqlUZNK8yC1uJNtT6iZzJaQrgFtd4J.png]

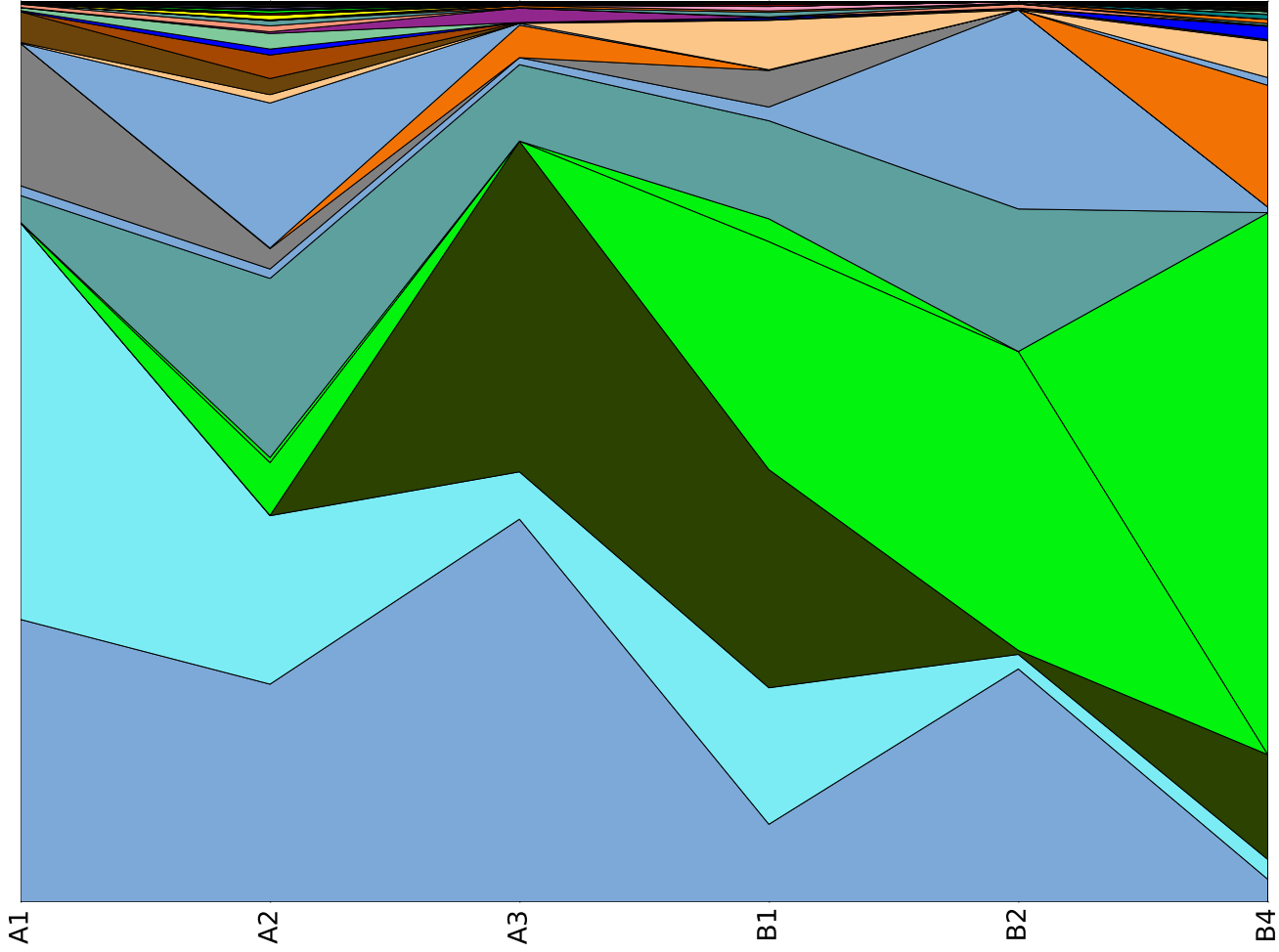

Supplement: Supplemental Information 1 [file peerj-08-9698-s001.zip › B07_taxa_summary/taxa_summary_plots/charts/J0s9MZGggylQTkzAxzSOcduxQFgj3L.pdf]

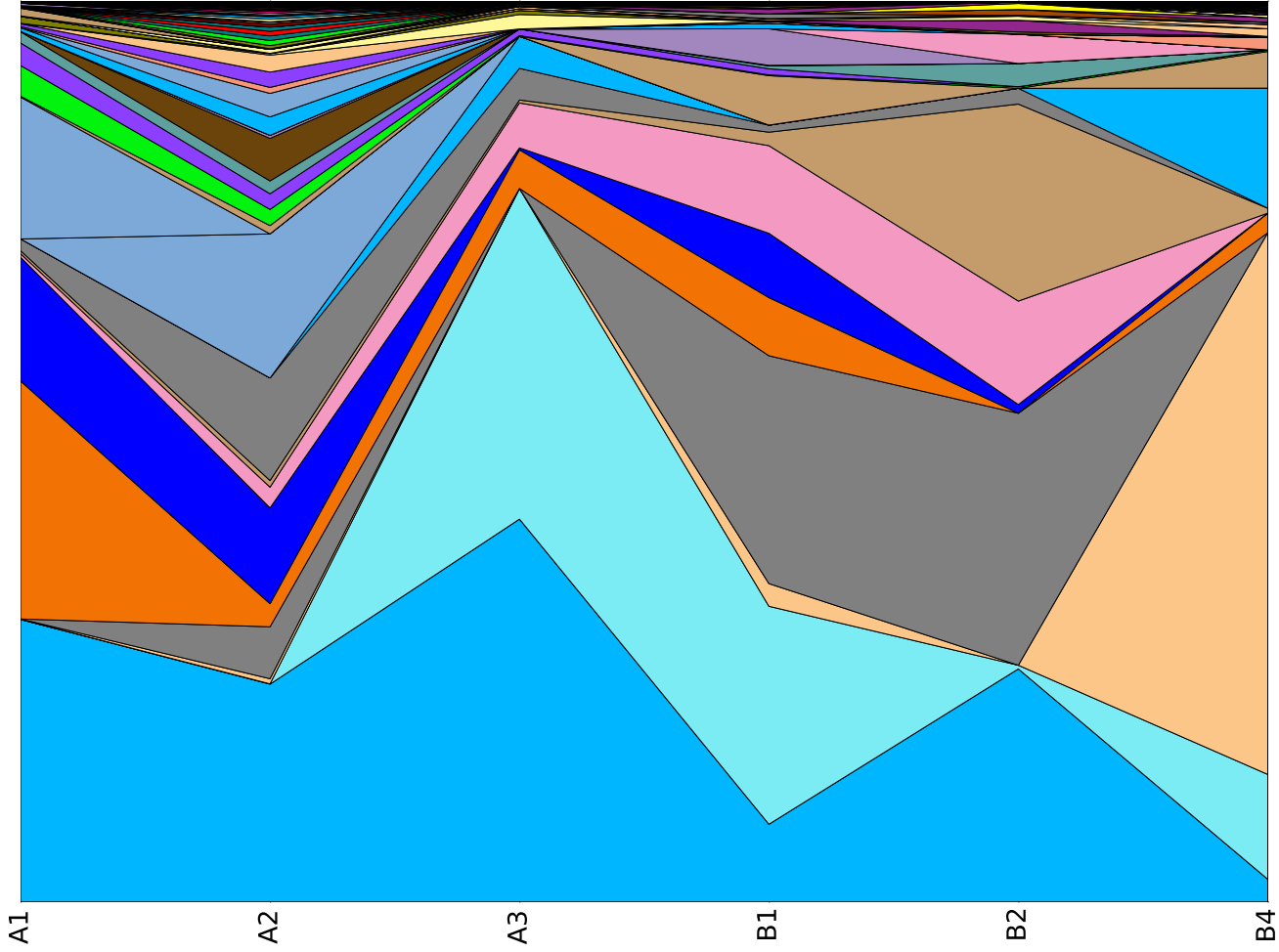

Supplement: Supplemental Information 1 [file peerj-08-9698-s001.zip › B07_taxa_summary/taxa_summary_plots/charts/o3IfZ8n21IHMz7qy0baPAt2TAWtqOu.pdf]

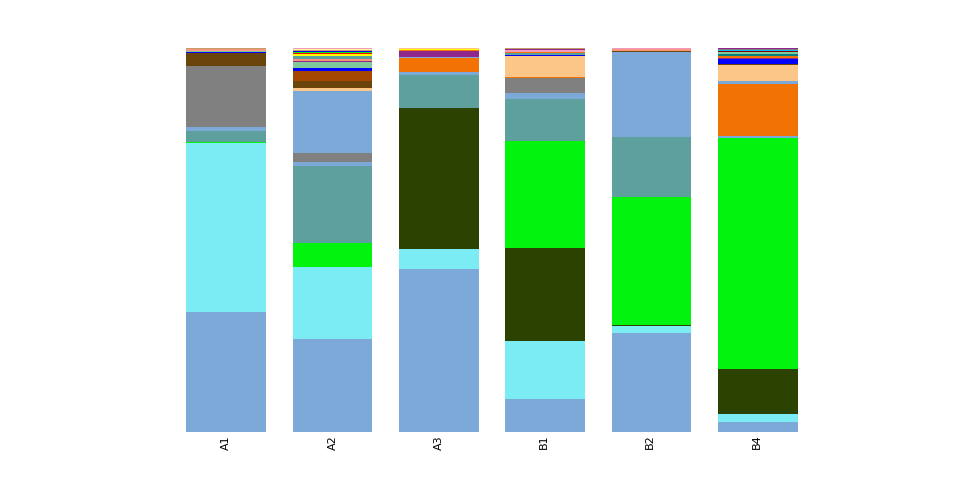

Supplement: Supplemental Information 1 [file peerj-08-9698-s001.zip › B07_taxa_summary/taxa_summary_plots/charts/odRiOOQHo4ZasiscpXYTfHy30aWhuP.png]

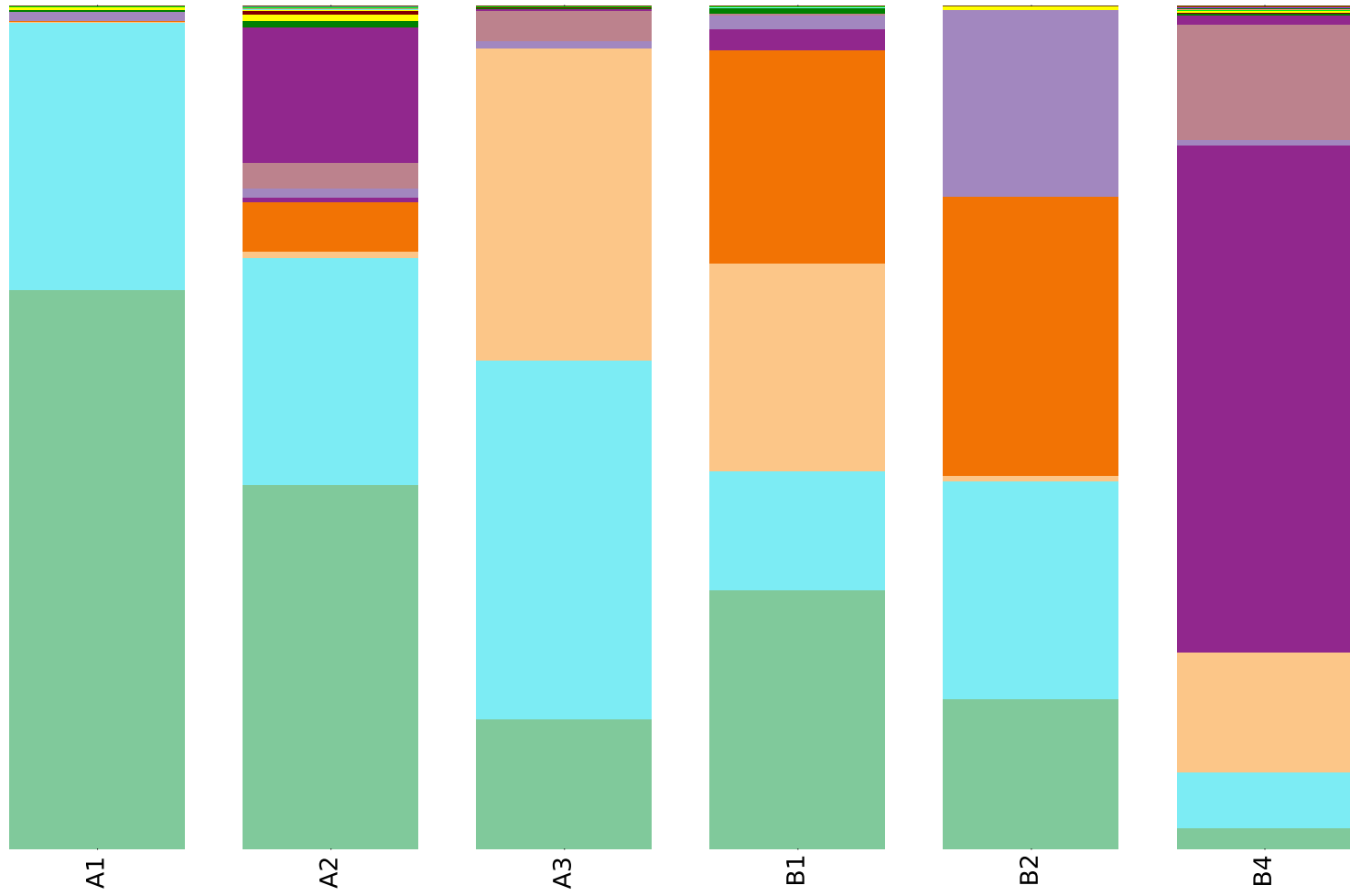

Supplement: Supplemental Information 1 [file peerj-08-9698-s001.zip › B07_taxa_summary/taxa_summary_plots/charts/P28hp76onYoAjsSiJPzkNcuPFKWOfK.pdf]

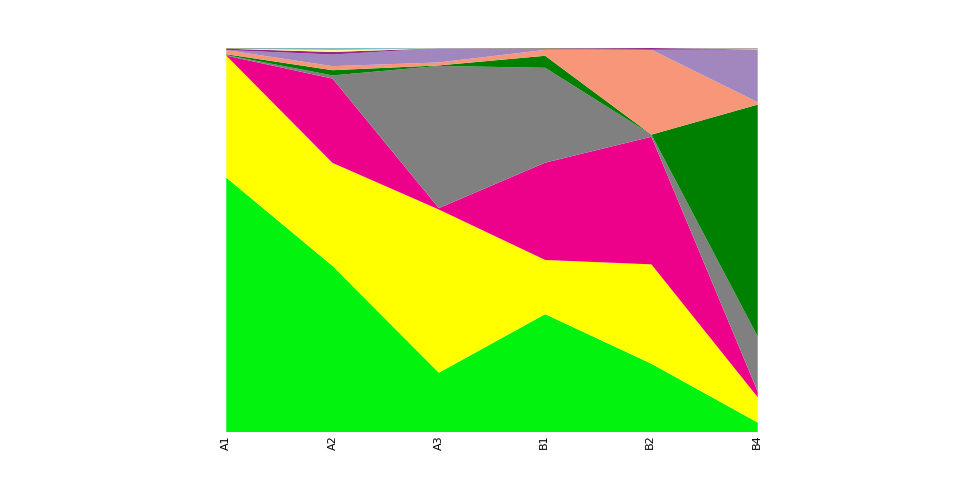

Supplement: Supplemental Information 1 [file peerj-08-9698-s001.zip › B07_taxa_summary/taxa_summary_plots/charts/pagn8H8lIPKLiJm0FYtbiqtBZu36wc.png]

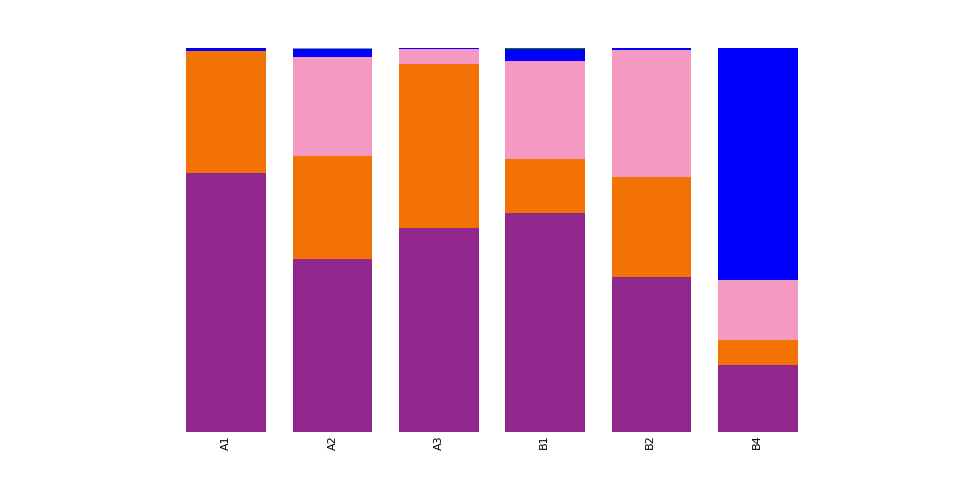

Supplement: Supplemental Information 1 [file peerj-08-9698-s001.zip › B07_taxa_summary/taxa_summary_plots/charts/PRYx0wyUpcSUQZD5erf1CtJPis4IgQ.png]

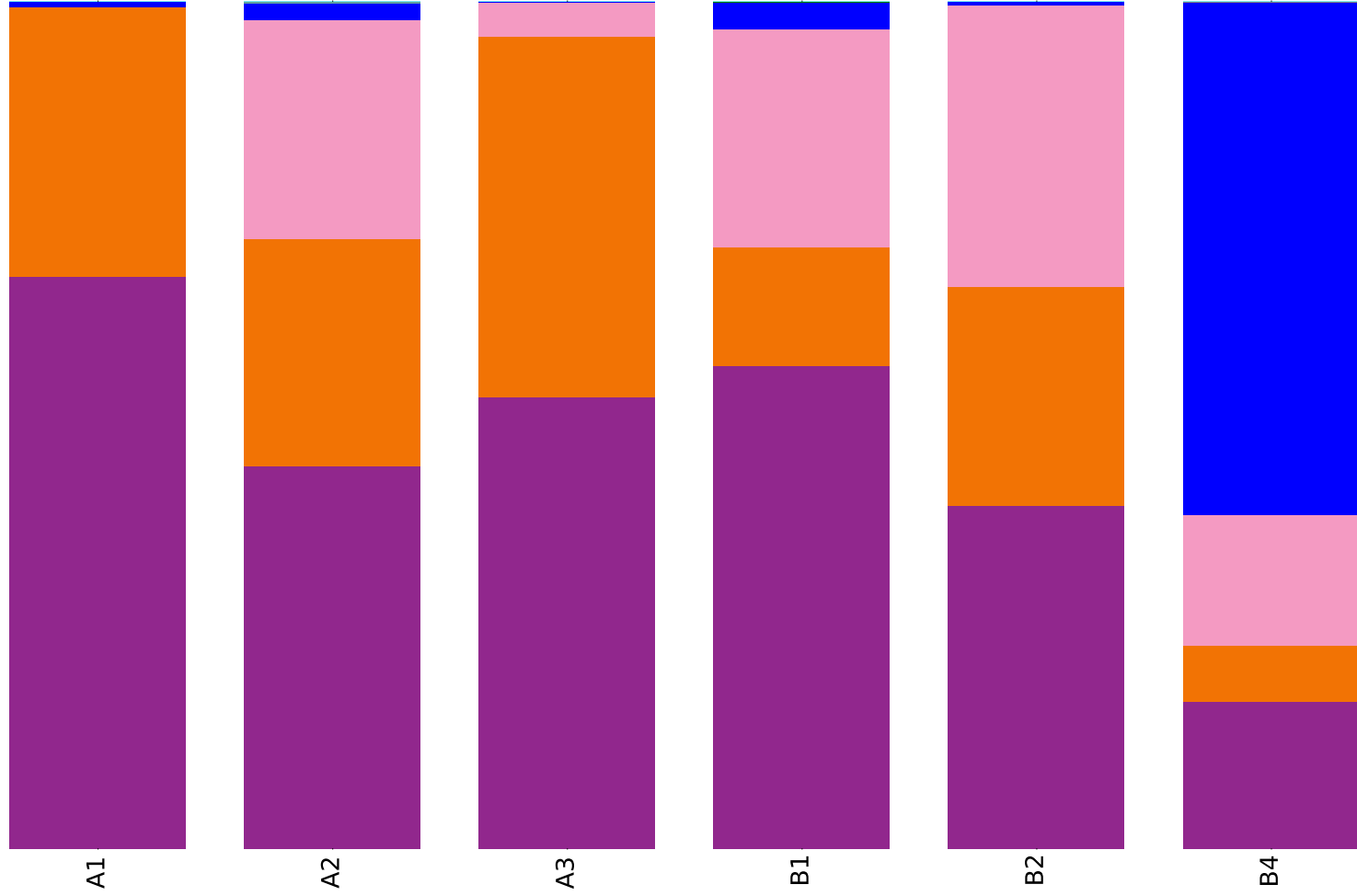

Supplement: Supplemental Information 1 [file peerj-08-9698-s001.zip › B07_taxa_summary/taxa_summary_plots/charts/r8QYGd0ZFXgZIQwM5ZgSQfZx4ZDaTJ.pdf]

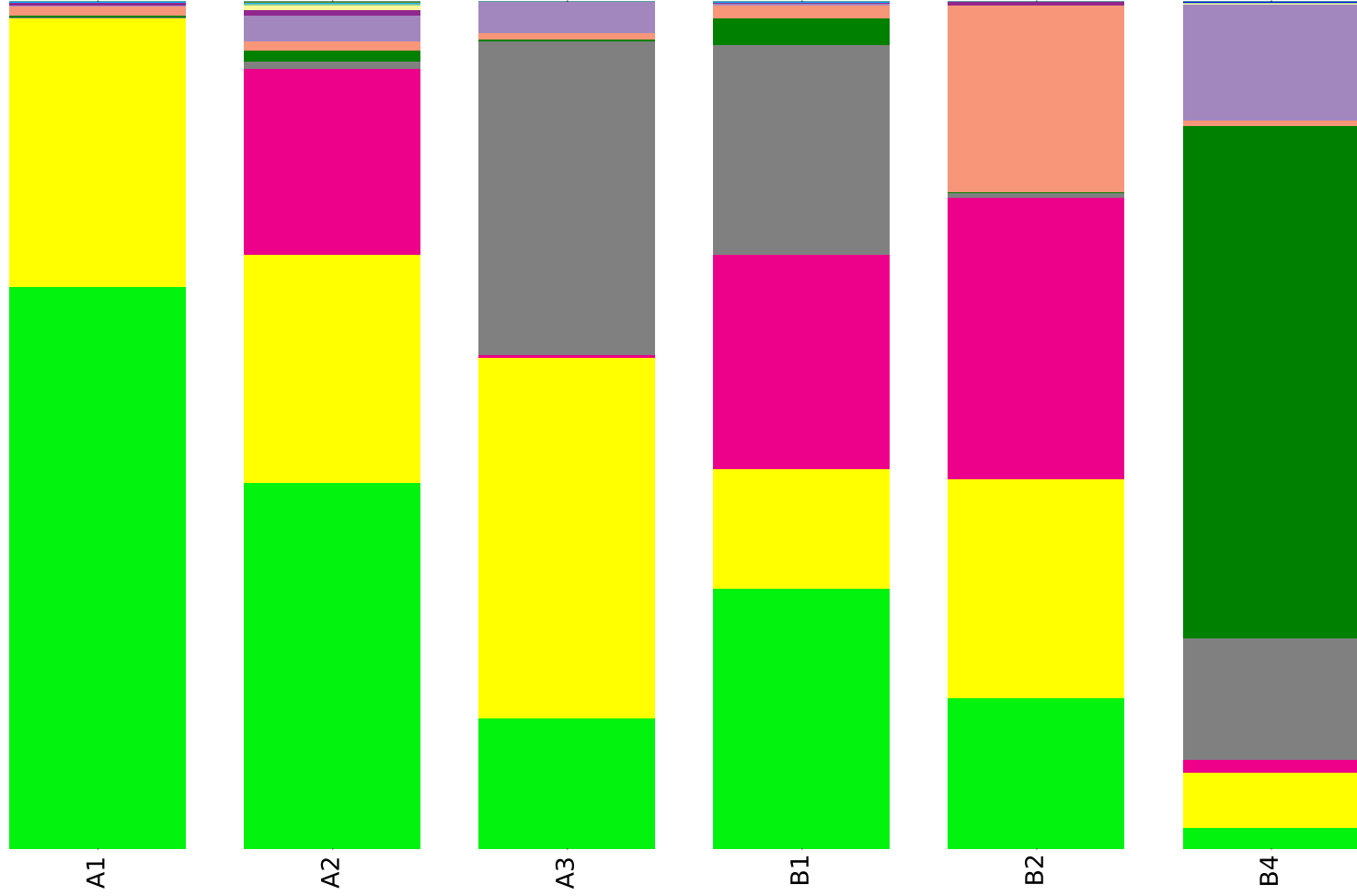

Supplement: Supplemental Information 1 [file peerj-08-9698-s001.zip › B07_taxa_summary/taxa_summary_plots/charts/zkjgq4S6yDoOfzeAZBZXR9jFymuEn3.pdf]

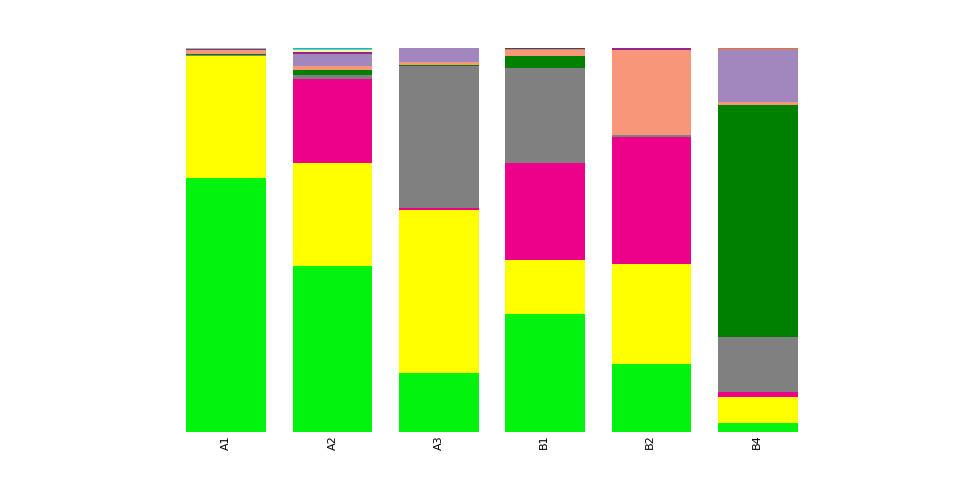

Supplement: Supplemental Information 1 [file peerj-08-9698-s001.zip › B07_taxa_summary/taxa_summary_plots/charts/ZWaYZ0srLhNNB0YAOCWbiUbmAaJa0J.png]

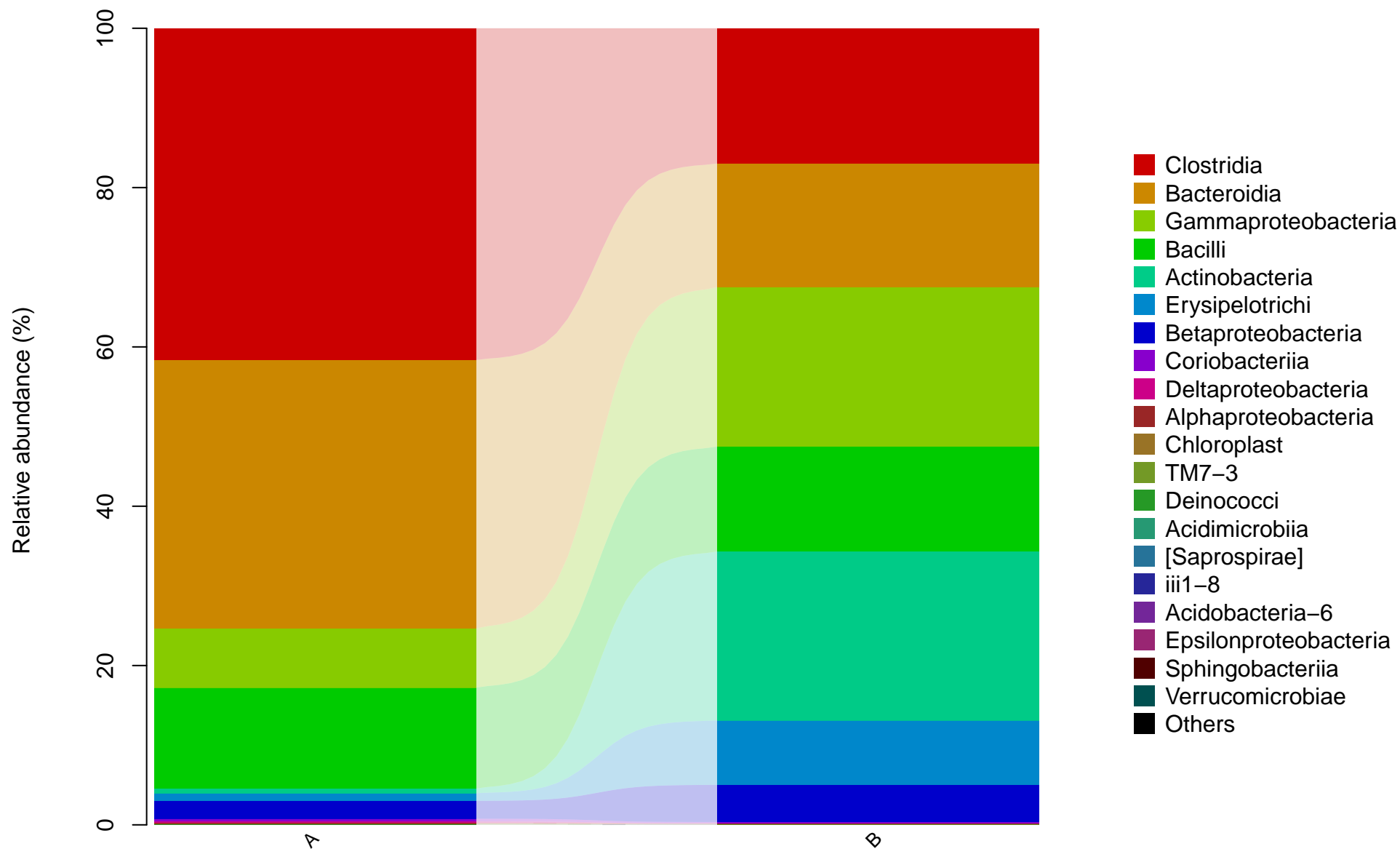

Supplement: Supplemental Information 1 [file peerj-08-9698-s001.zip › B07_taxa_summary_group/bar_class.pdf]

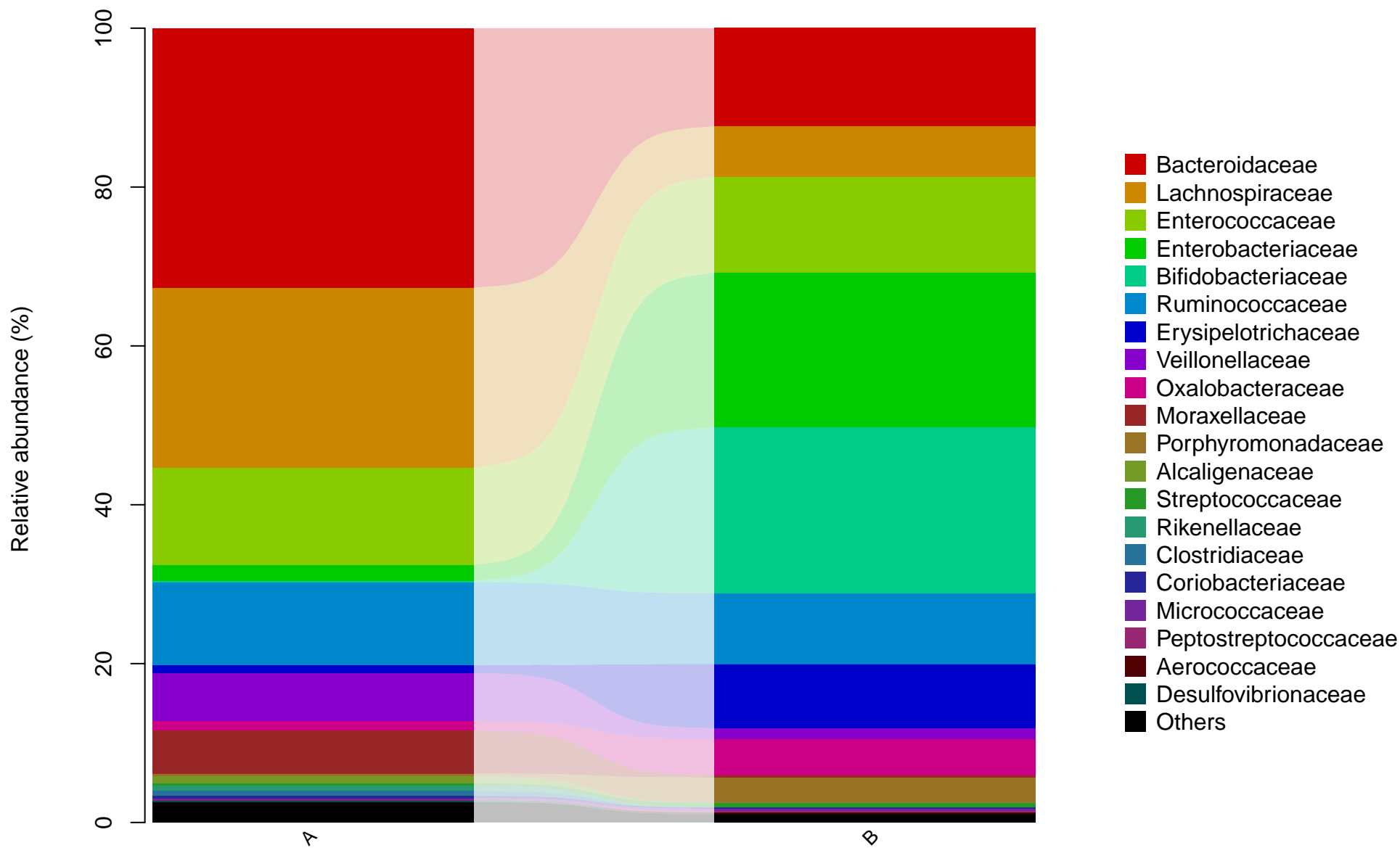

Supplement: Supplemental Information 1 [file peerj-08-9698-s001.zip › B07_taxa_summary_group/bar_family.pdf]

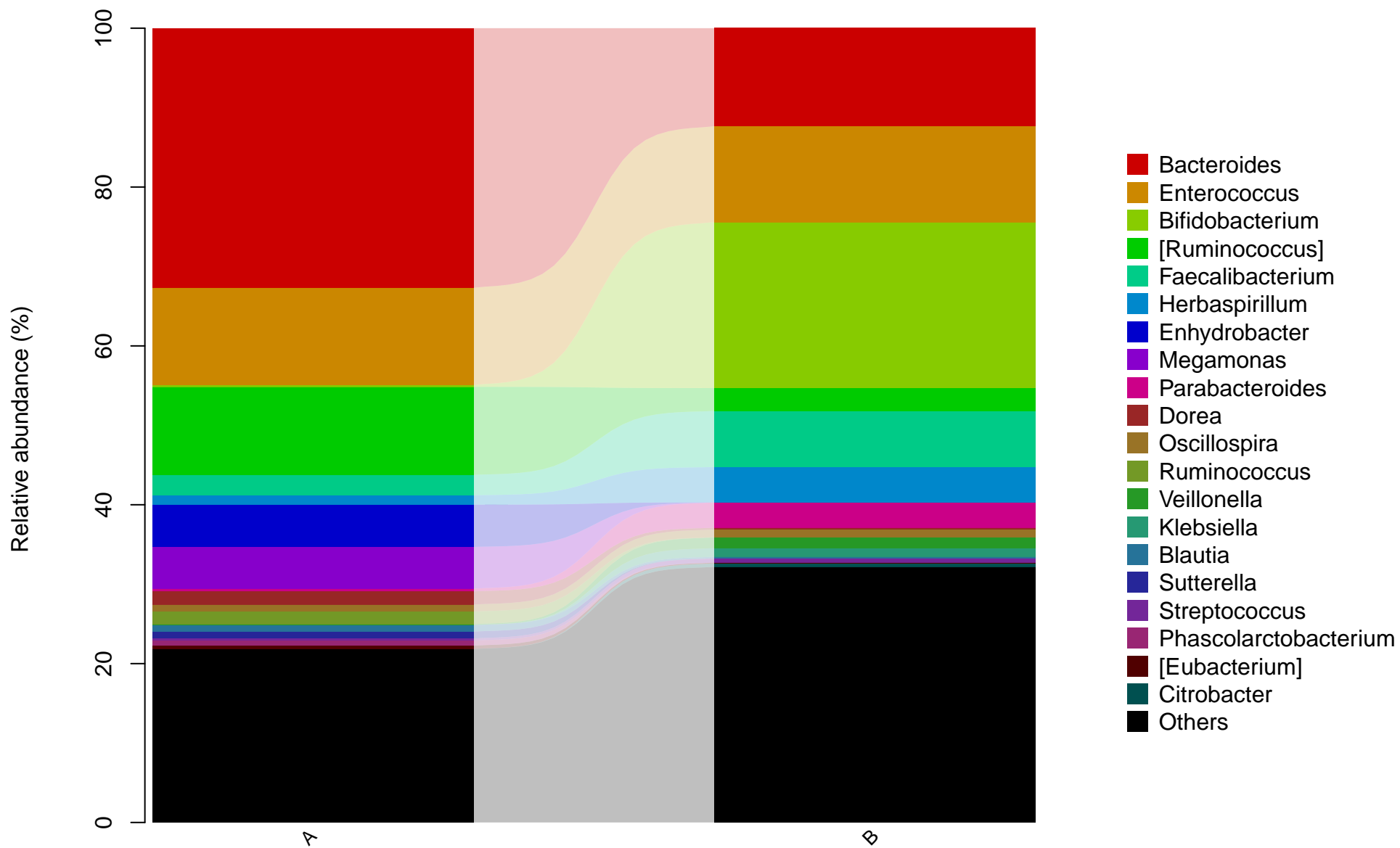

Supplement: Supplemental Information 1 [file peerj-08-9698-s001.zip › B07_taxa_summary_group/bar_genus.pdf]

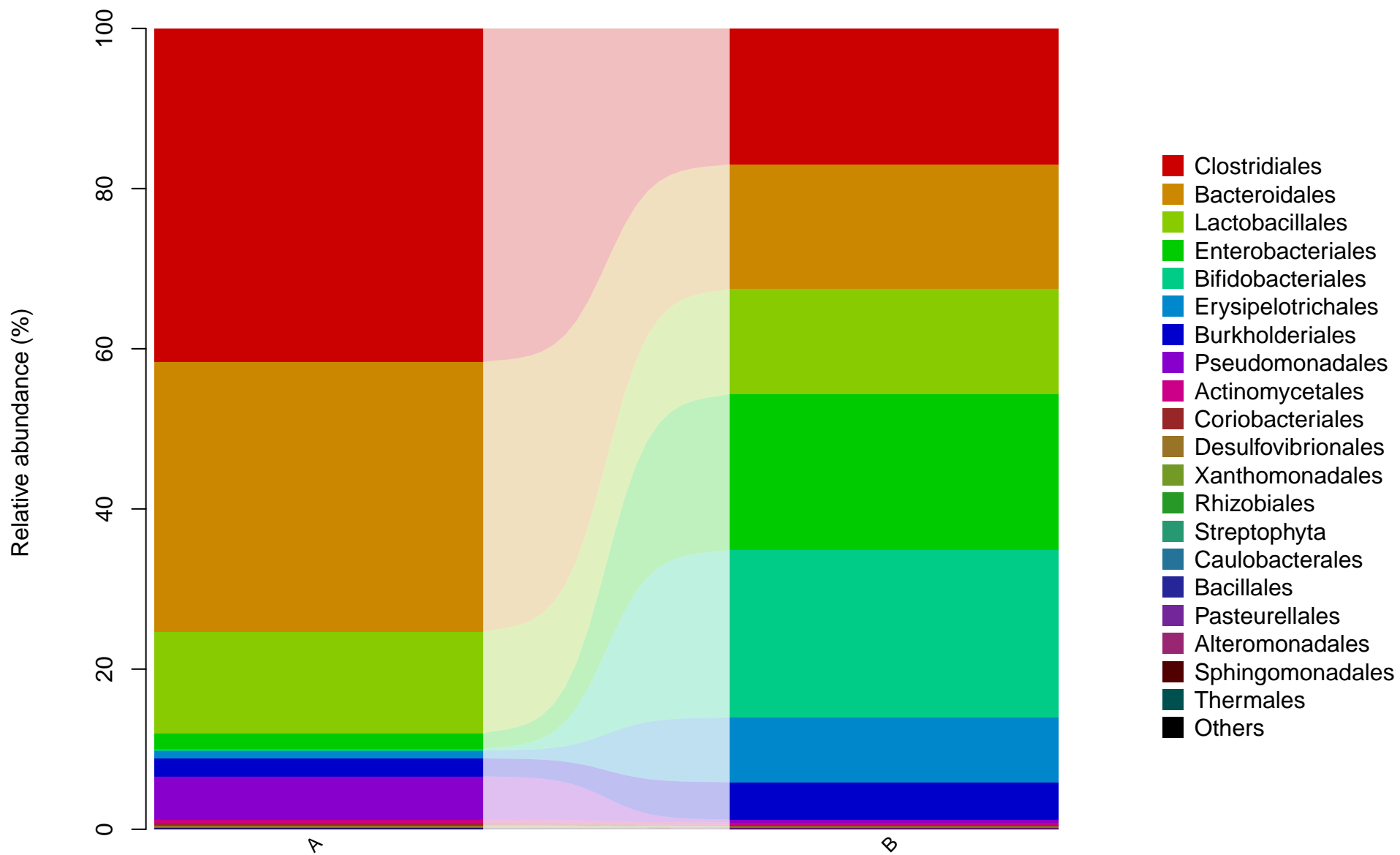

Supplement: Supplemental Information 1 [file peerj-08-9698-s001.zip › B07_taxa_summary_group/bar_order.pdf]

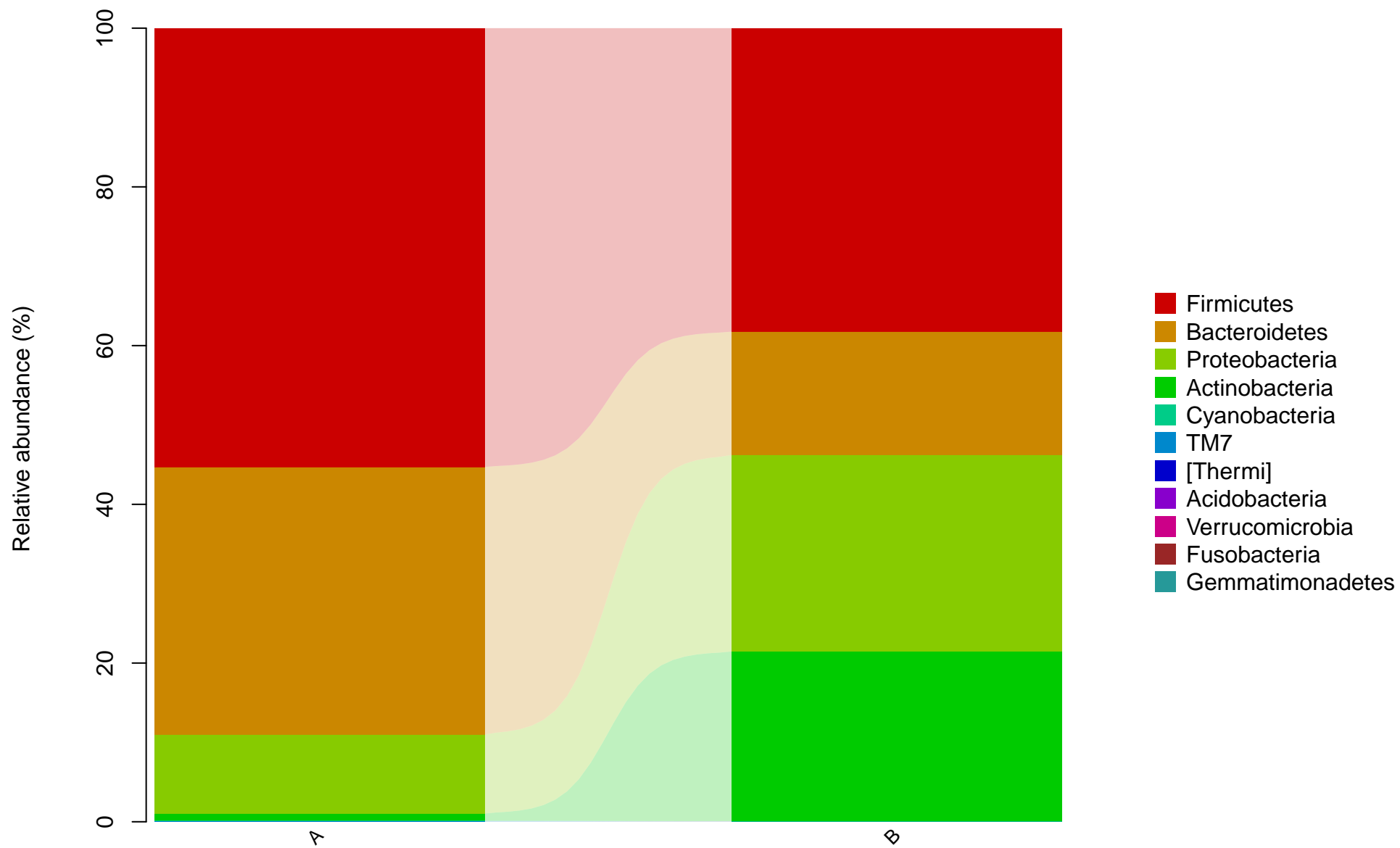

Supplement: Supplemental Information 1 [file peerj-08-9698-s001.zip › B07_taxa_summary_group/bar_phylum.pdf]

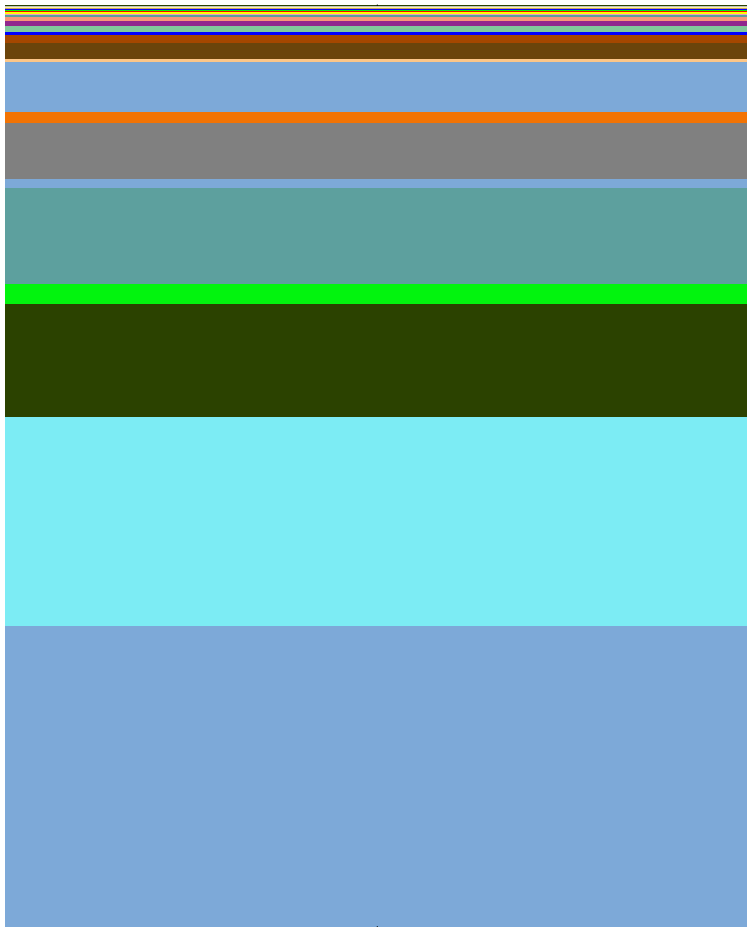

A

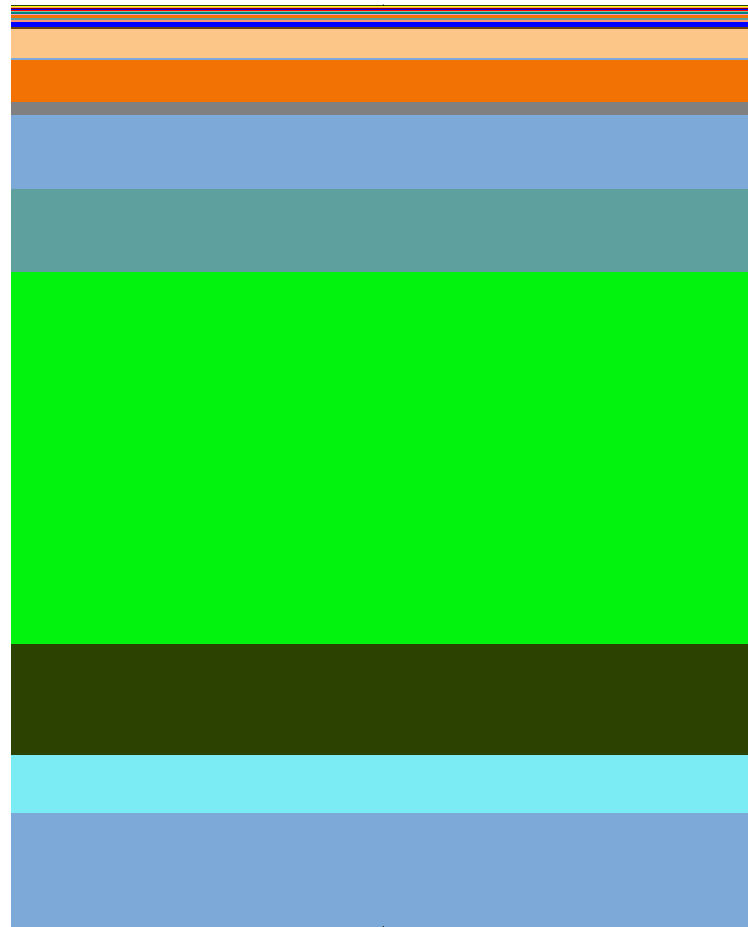

B

Supplement: Supplemental Information 1 [file peerj-08-9698-s001.zip › B07_taxa_summary_group/taxa_summary_plots/charts/35bHF0yzzpdDYoPiInDiAkLPHkJwZx.pdf]

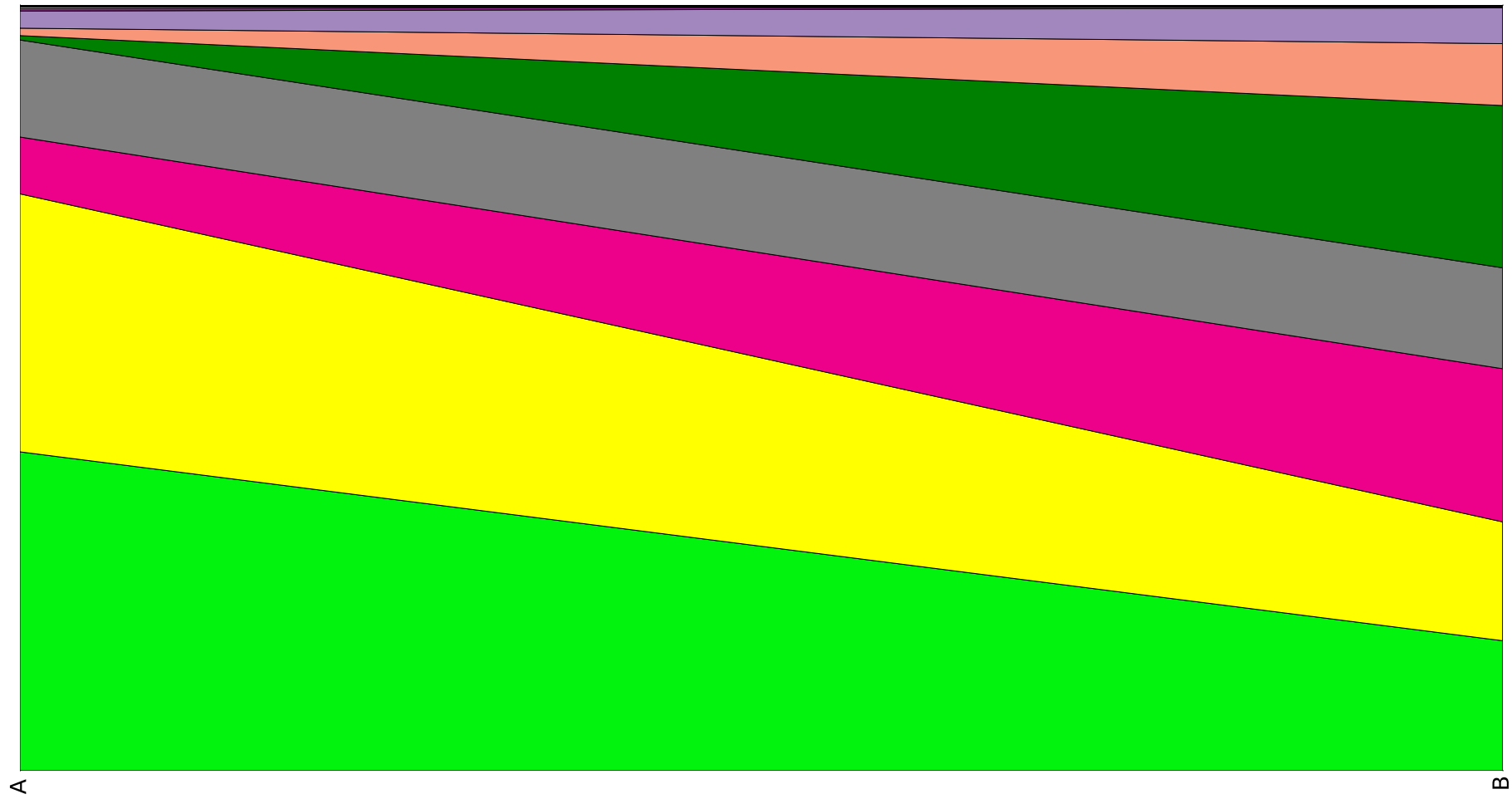

Supplement: Supplemental Information 1 [file peerj-08-9698-s001.zip › B07_taxa_summary_group/taxa_summary_plots/charts/4d7WP6MYmT0qqow01bbcHK0A8K4aj2.pdf]

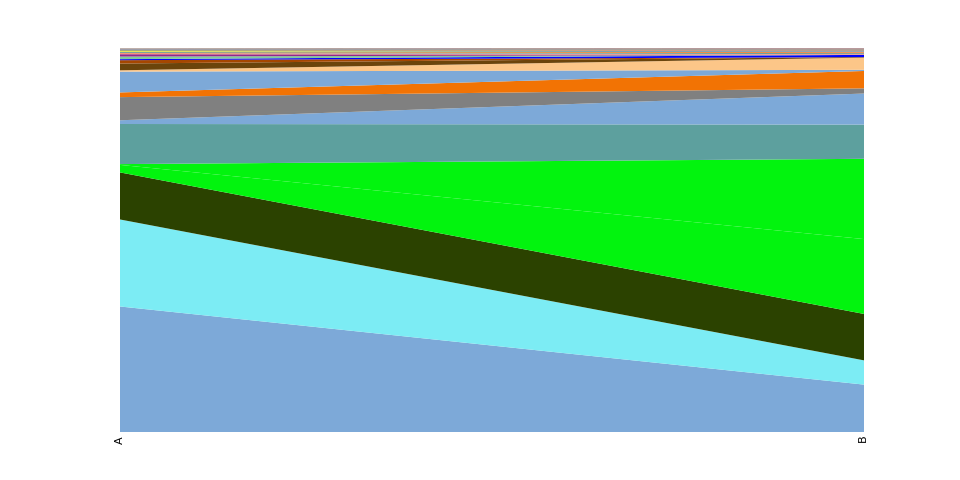

Supplement: Supplemental Information 1 [file peerj-08-9698-s001.zip › B07_taxa_summary_group/taxa_summary_plots/charts/6AkQo32SzNlarbyDJPIB59036ReBO2.png]

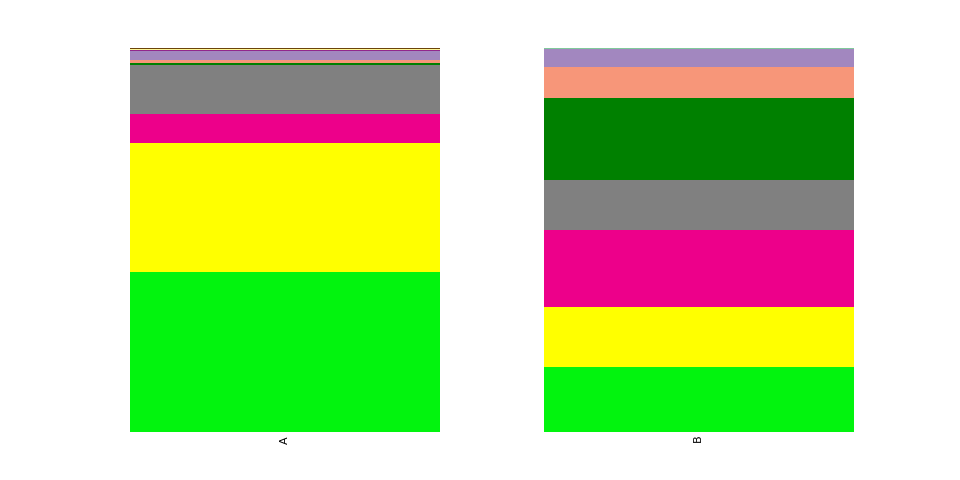

Supplement: Supplemental Information 1 [file peerj-08-9698-s001.zip › B07_taxa_summary_group/taxa_summary_plots/charts/7LKXY0N7DEuZA01fYeYcDPs1Jd1PEj.png]

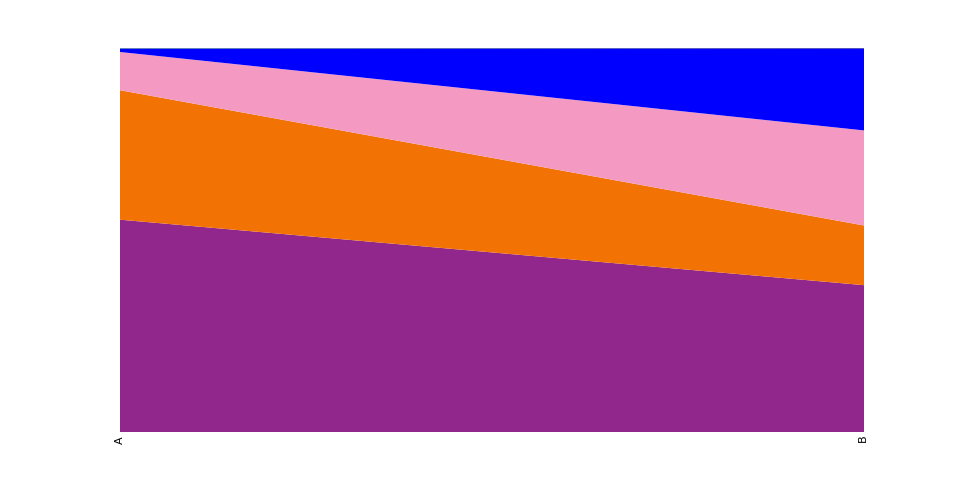

Supplement: Supplemental Information 1 [file peerj-08-9698-s001.zip › B07_taxa_summary_group/taxa_summary_plots/charts/9FmWJuktRUaGHUHMczTfPXztZ6OczP.png]

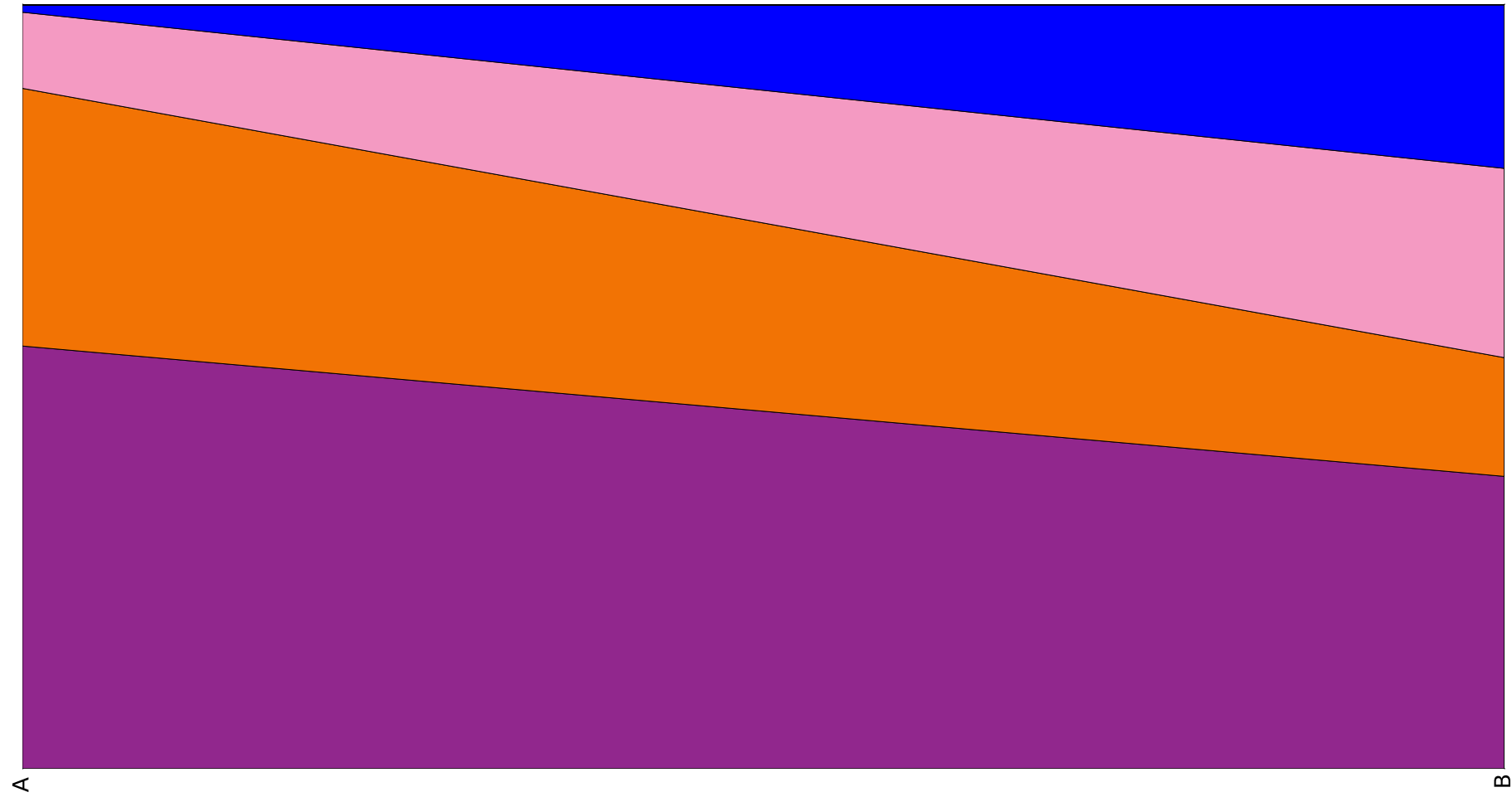

Supplement: Supplemental Information 1 [file peerj-08-9698-s001.zip › B07_taxa_summary_group/taxa_summary_plots/charts/aQa8fryzQLpmcj5mMXscq7H2qY1tqL.pdf]

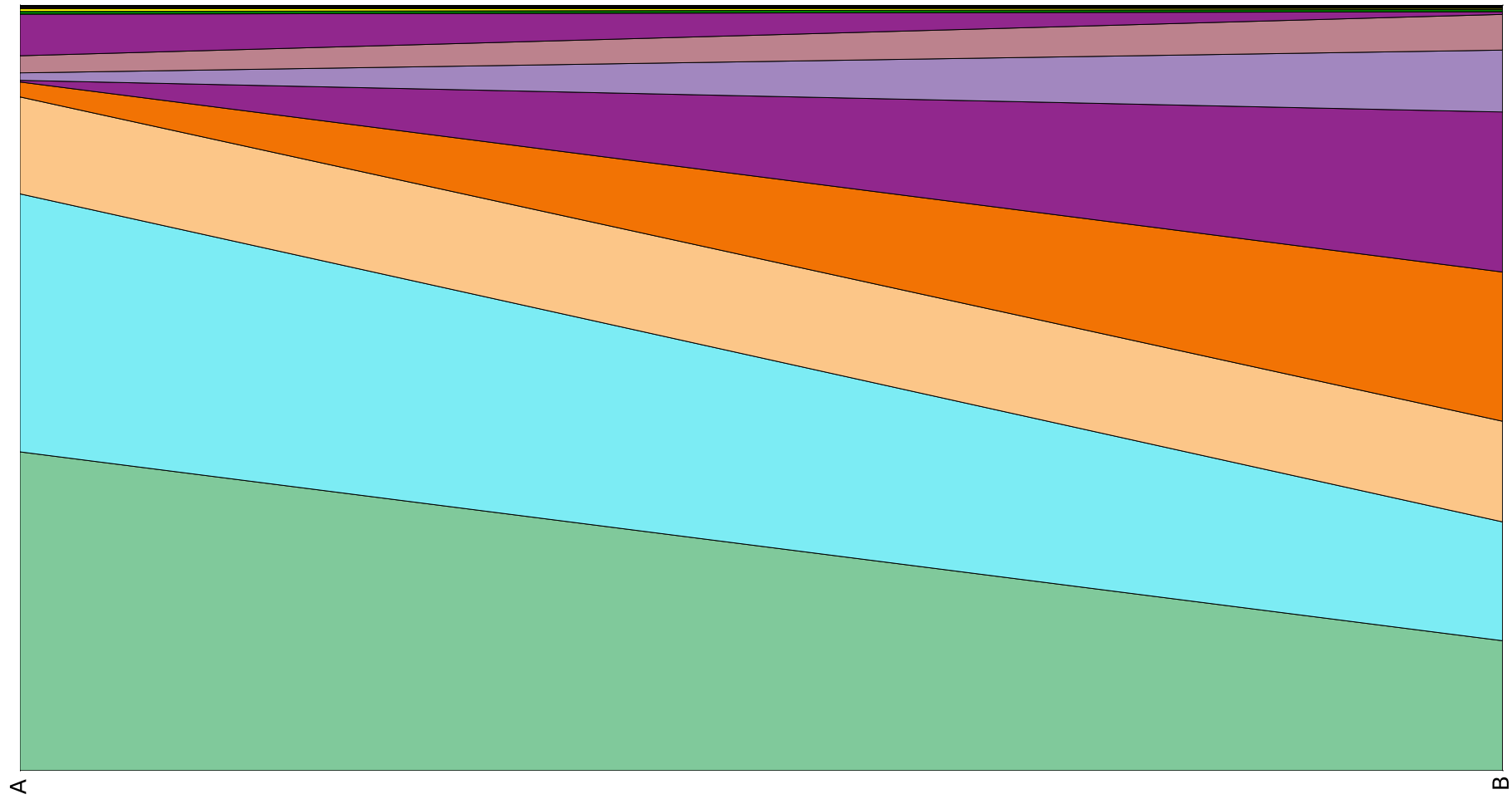

Supplement: Supplemental Information 1 [file peerj-08-9698-s001.zip › B07_taxa_summary_group/taxa_summary_plots/charts/bZGFpSZ1LxScUXKfdiDIoFtdB0Sucp.pdf]

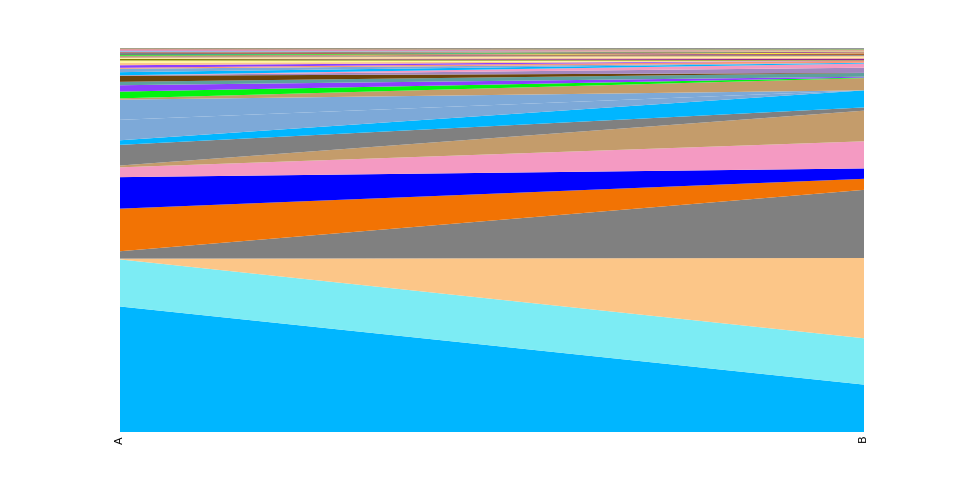

Supplement: Supplemental Information 1 [file peerj-08-9698-s001.zip › B07_taxa_summary_group/taxa_summary_plots/charts/C0ZR9XaaCT3UjTE27TxFd2mzSC3rTK.png]

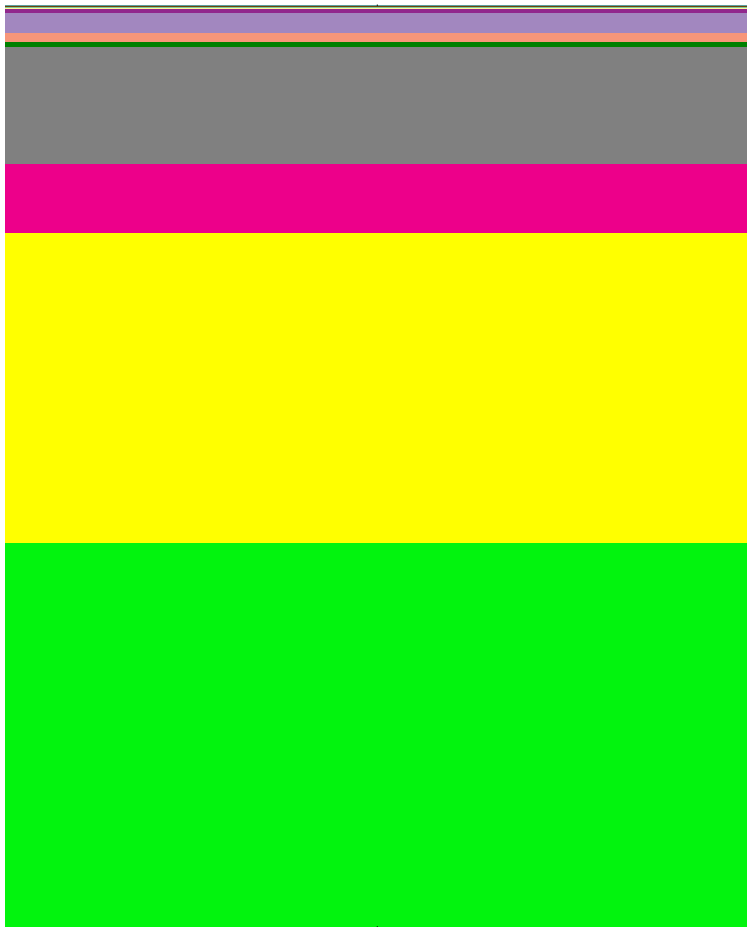

A

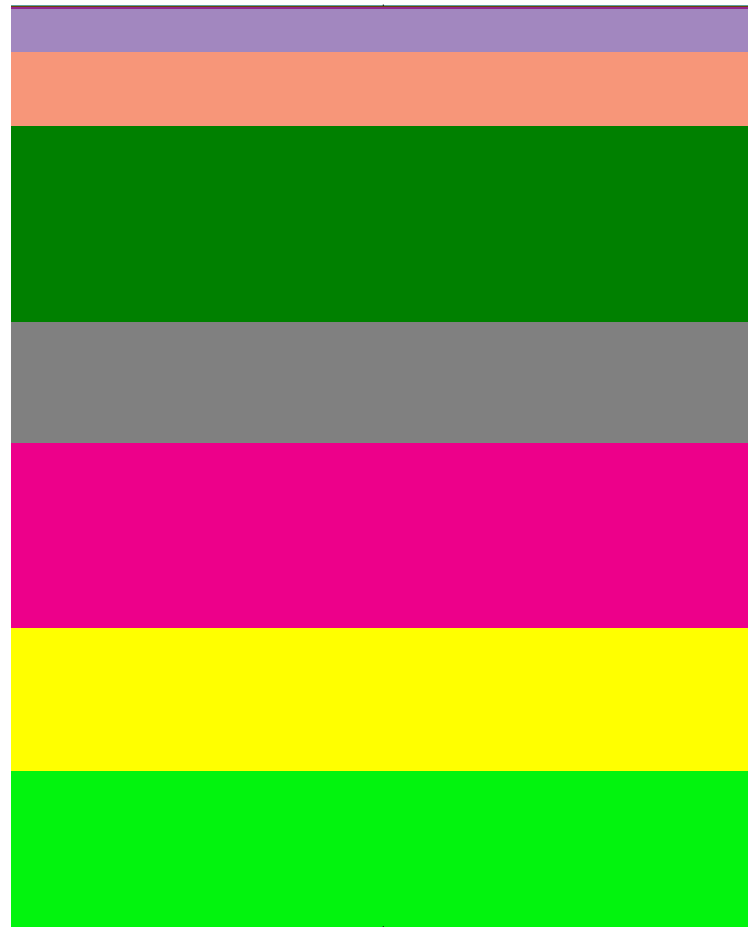

B

Supplement: Supplemental Information 1 [file peerj-08-9698-s001.zip › B07_taxa_summary_group/taxa_summary_plots/charts/FwDucrxppyBempeifzMYgbzXXXl0qX.pdf]

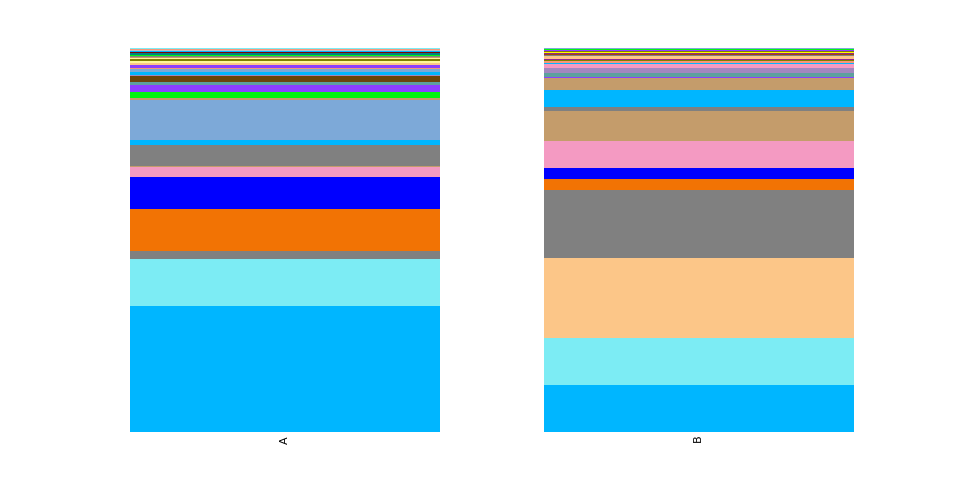

Supplement: Supplemental Information 1 [file peerj-08-9698-s001.zip › B07_taxa_summary_group/taxa_summary_plots/charts/g88it2ZmYyWyp4ZglyLkqb2BungayJ.png]

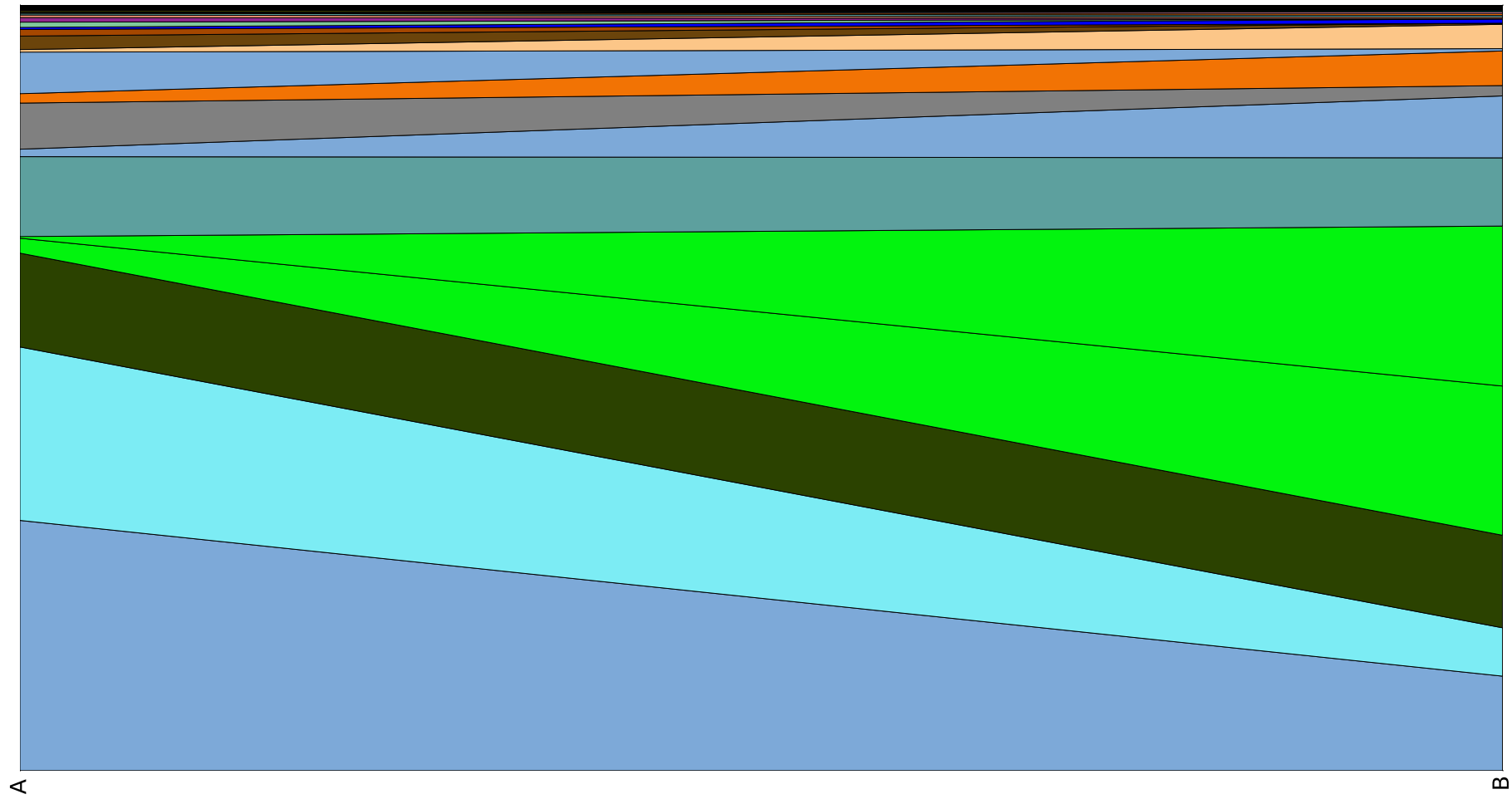

Supplement: Supplemental Information 1 [file peerj-08-9698-s001.zip › B07_taxa_summary_group/taxa_summary_plots/charts/I7mgiCrTTbLHZEbK3xc4fHXEjbaWek.pdf]

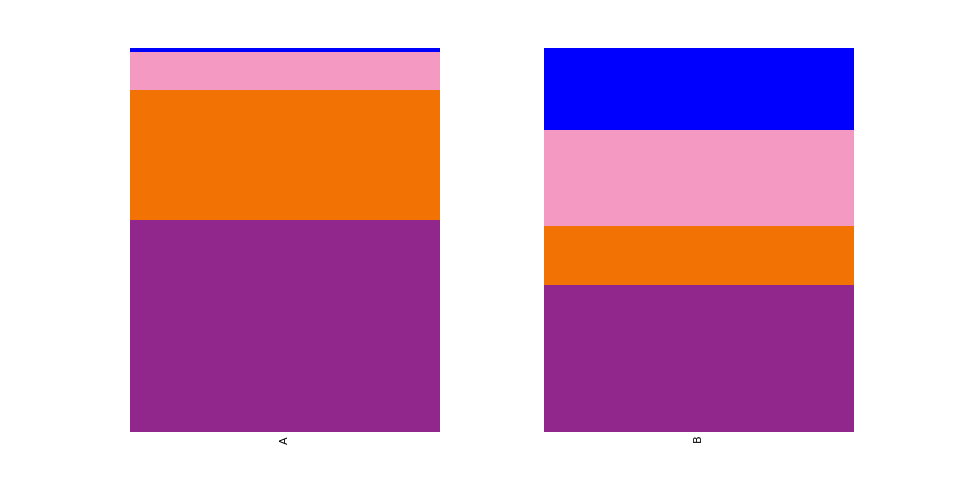

Supplement: Supplemental Information 1 [file peerj-08-9698-s001.zip › B07_taxa_summary_group/taxa_summary_plots/charts/i7SqO1gXp6rkH4OAADz75SjZya2E60.png]

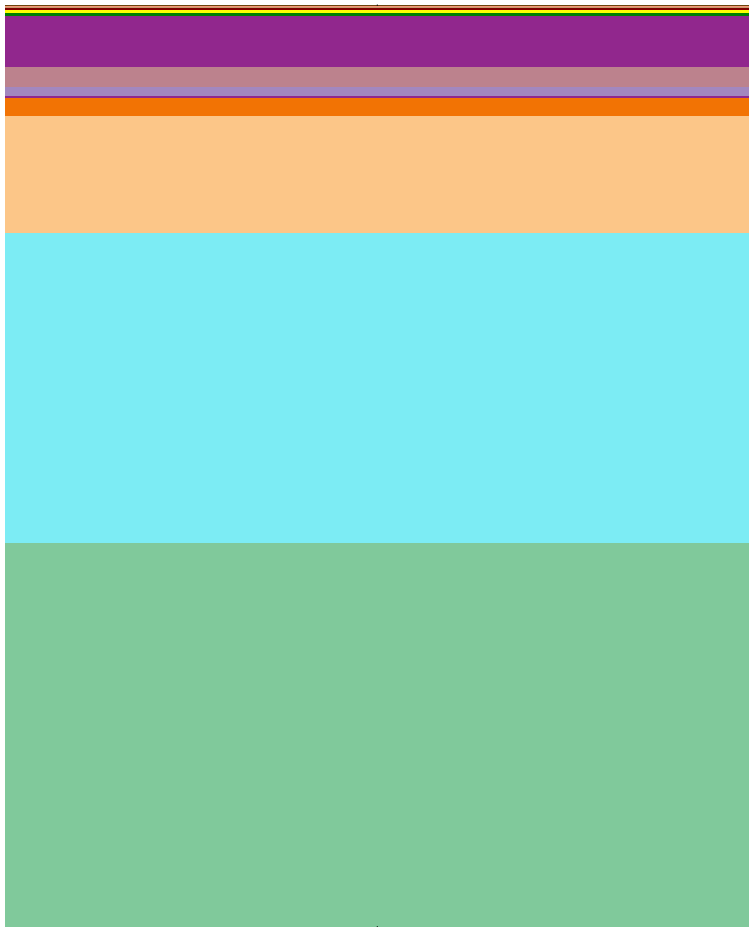

A

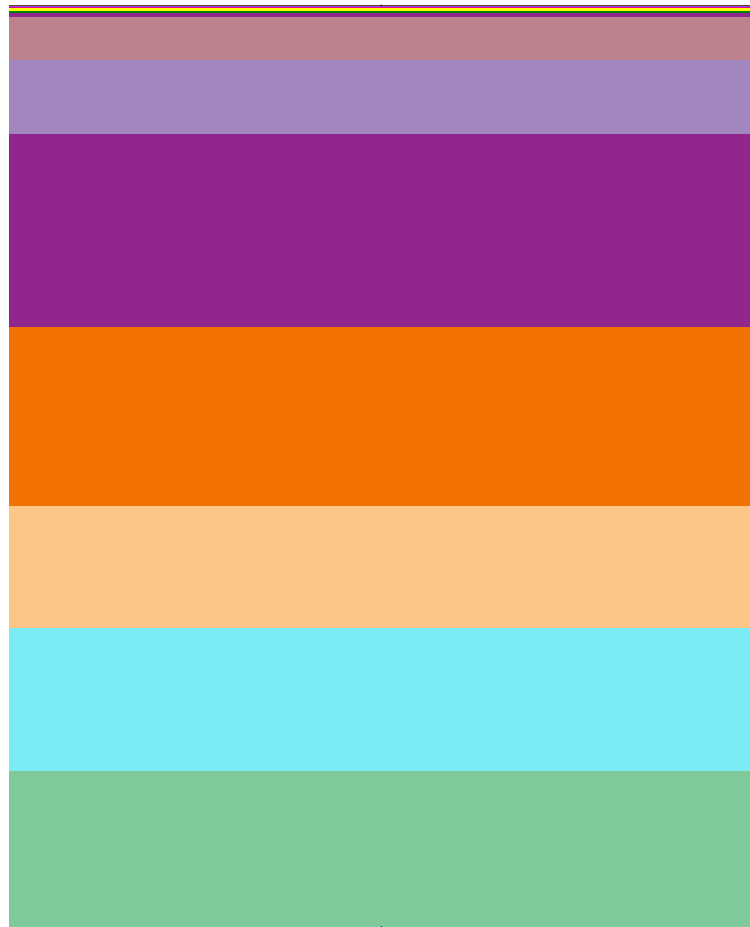

B

Supplement: Supplemental Information 1 [file peerj-08-9698-s001.zip › B07_taxa_summary_group/taxa_summary_plots/charts/MCAcJ2bfX0Pnf3sRQFPOtSwk6nTgwe.pdf]

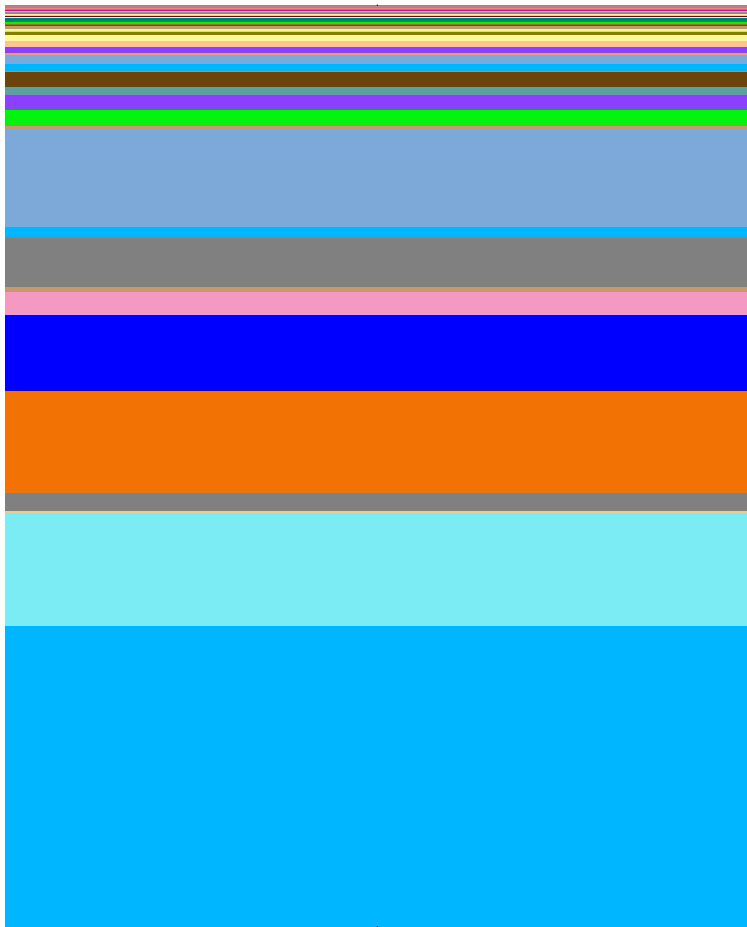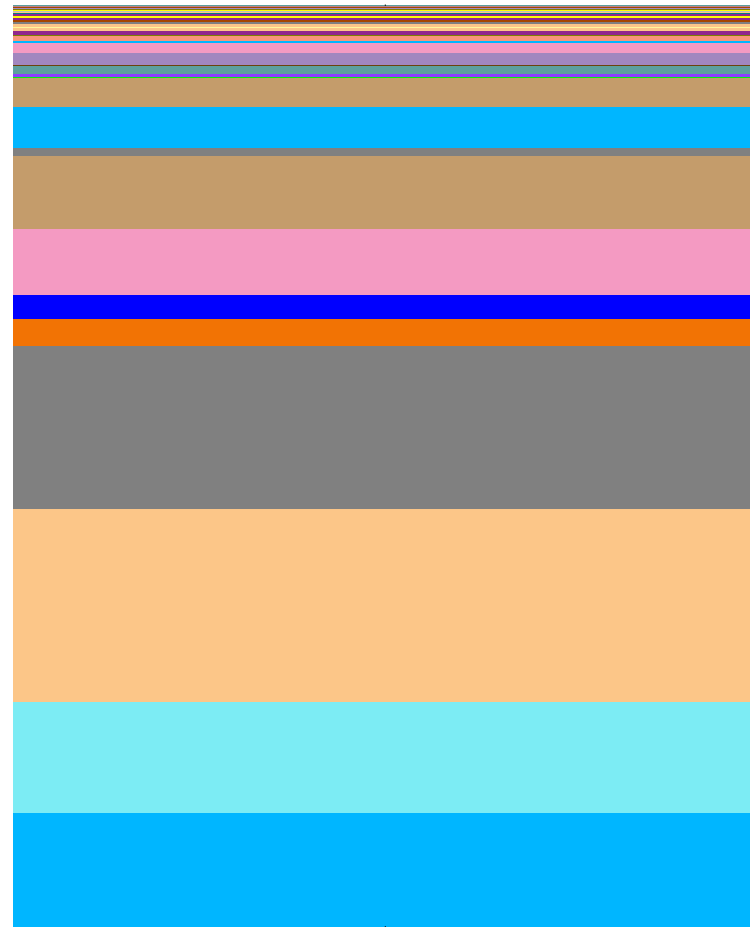

Supplement: Supplemental Information 1 [file peerj-08-9698-s001.zip › B07_taxa_summary_group/taxa_summary_plots/charts/P3iziCSKIe15Wz0NxqtwglzHzPdjXS.pdf]

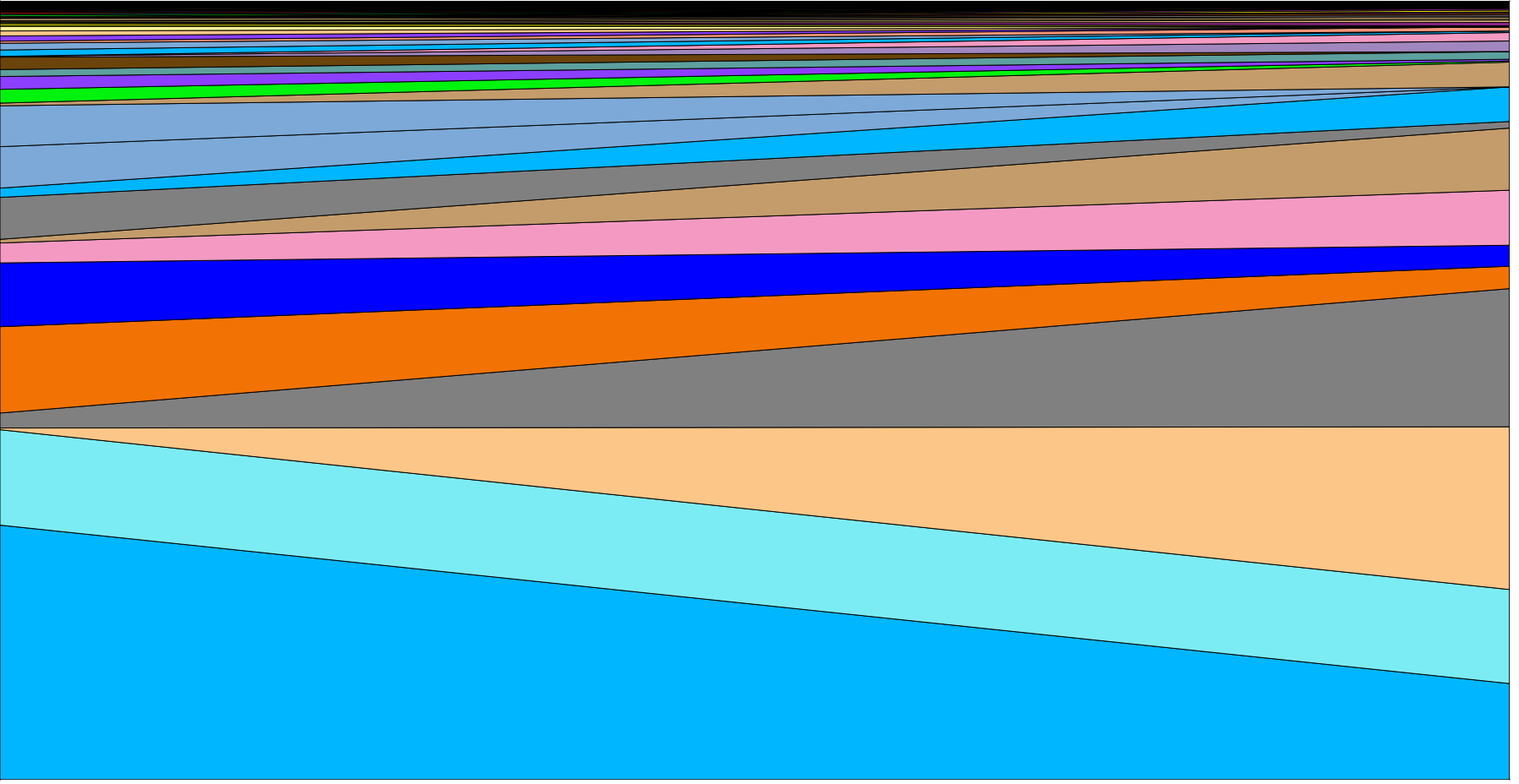

A

B

Supplement: Supplemental Information 1 [file peerj-08-9698-s001.zip › B07_taxa_summary_group/taxa_summary_plots/charts/RQ0jY0WLf7lkUZX54U3hFajQ1MkqWt.pdf]

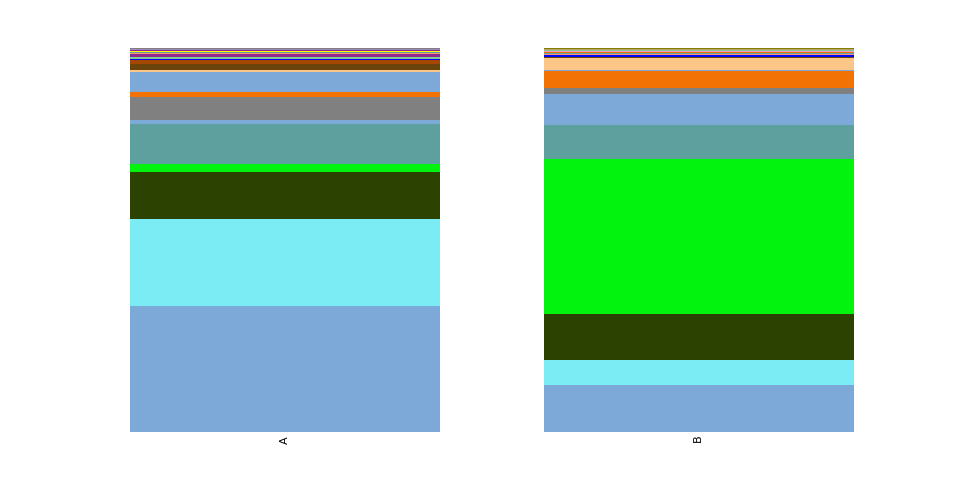

Supplement: Supplemental Information 1 [file peerj-08-9698-s001.zip › B07_taxa_summary_group/taxa_summary_plots/charts/ud7ZLFq9w9YyQ75ZS4QNMUbmZ3SbPR.png]

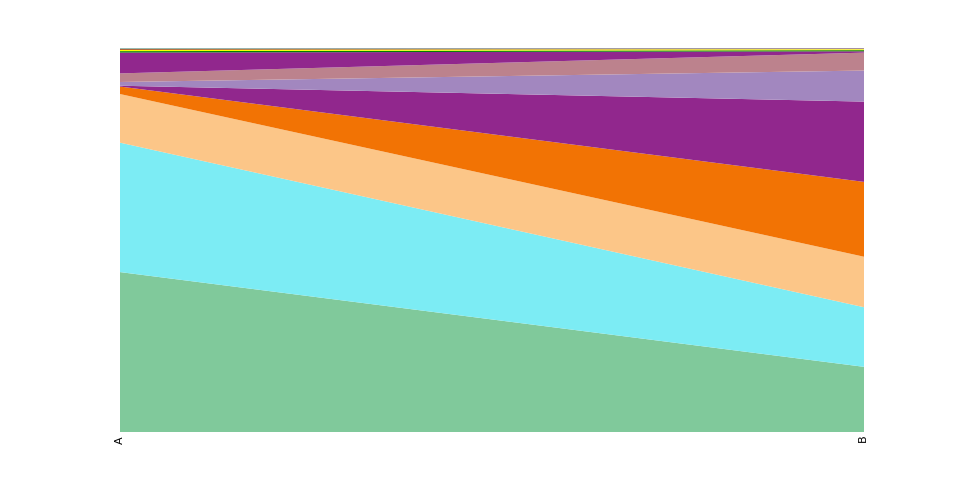

Supplement: Supplemental Information 1 [file peerj-08-9698-s001.zip › B07_taxa_summary_group/taxa_summary_plots/charts/WU8ul6LliIj33hpB0z20ELpRIA0eCE.png]

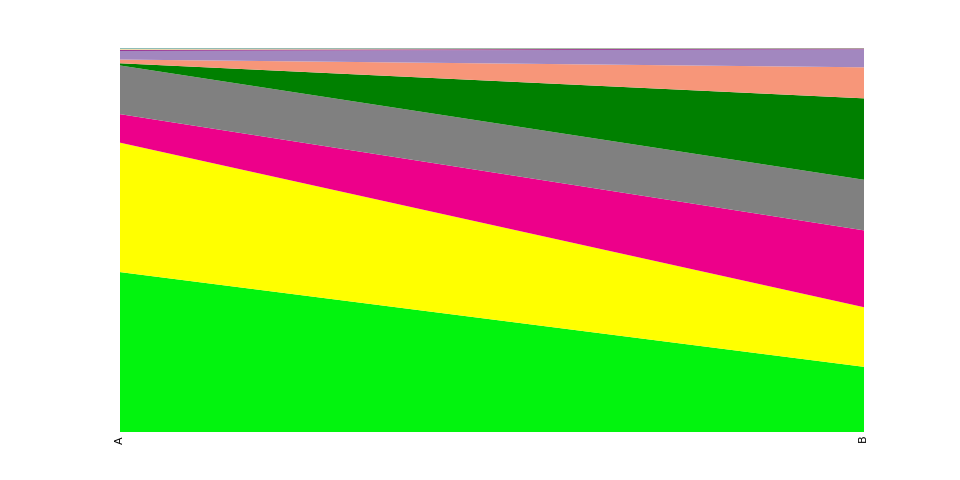

Supplement: Supplemental Information 1 [file peerj-08-9698-s001.zip › B07_taxa_summary_group/taxa_summary_plots/charts/Y33sJ42RYEpqrleAYQWlmZxTZ50bZd.png]

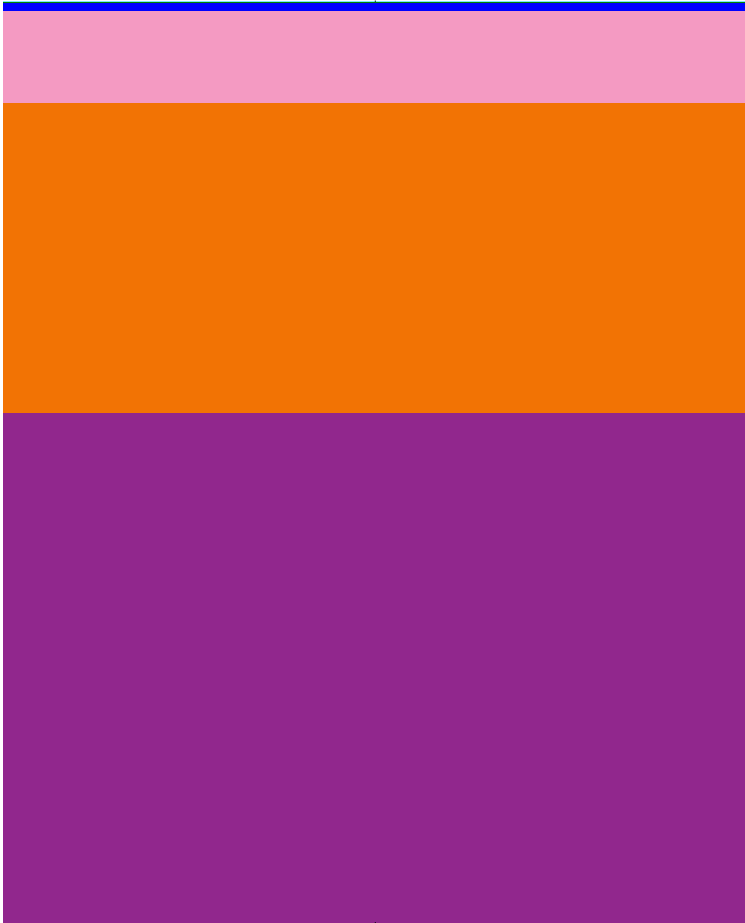

A

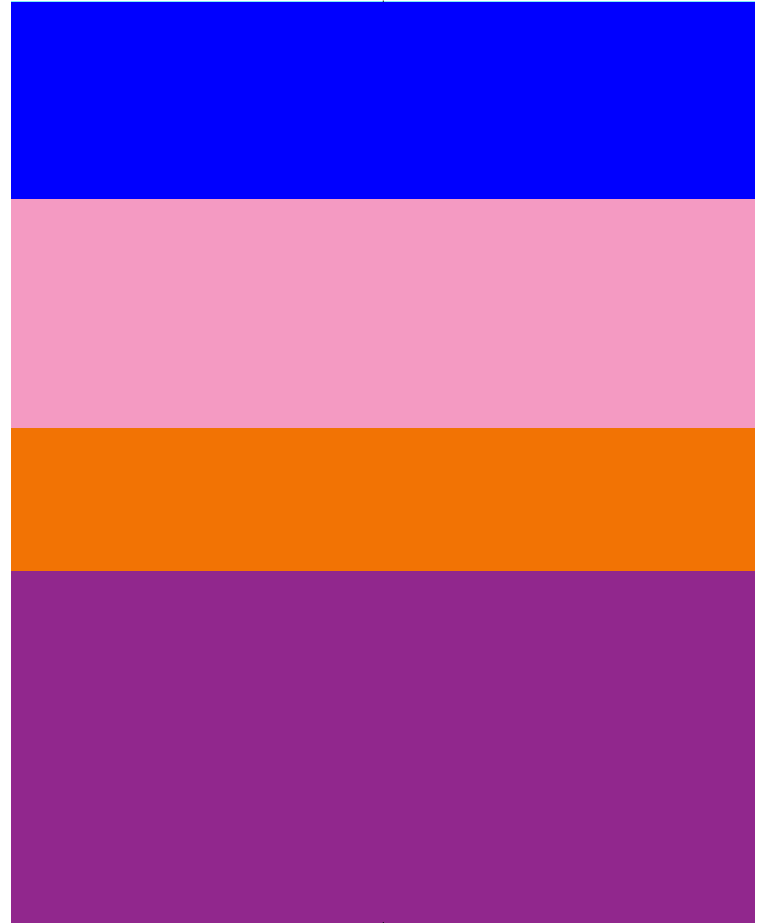

B

Supplement: Supplemental Information 1 [file peerj-08-9698-s001.zip › B07_taxa_summary_group/taxa_summary_plots/charts/y4byPEMI0uQAe8wmmW8sXLZsZ6k0Nm.pdf]

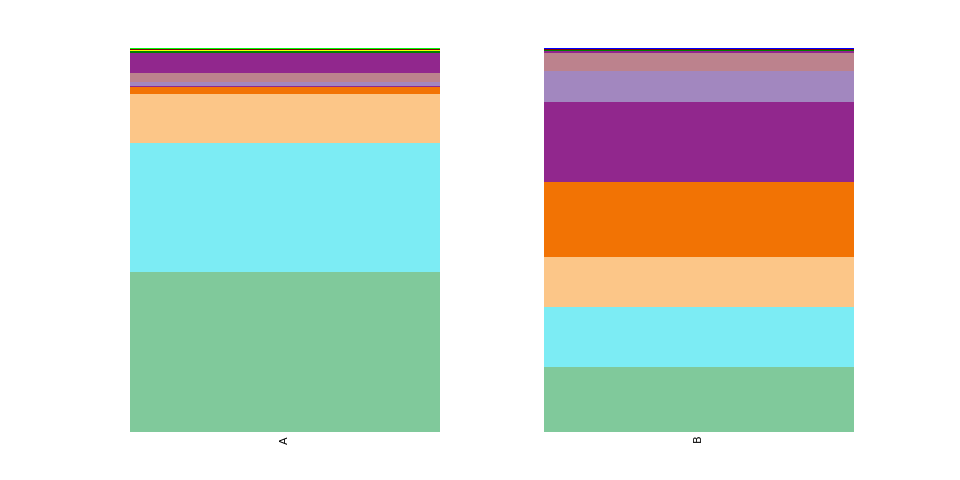

Supplement: Supplemental Information 1 [file peerj-08-9698-s001.zip › B07_taxa_summary_group/taxa_summary_plots/charts/ysXNz6UQ54ObMzMFr3AhS8CPS4OEH2.png]

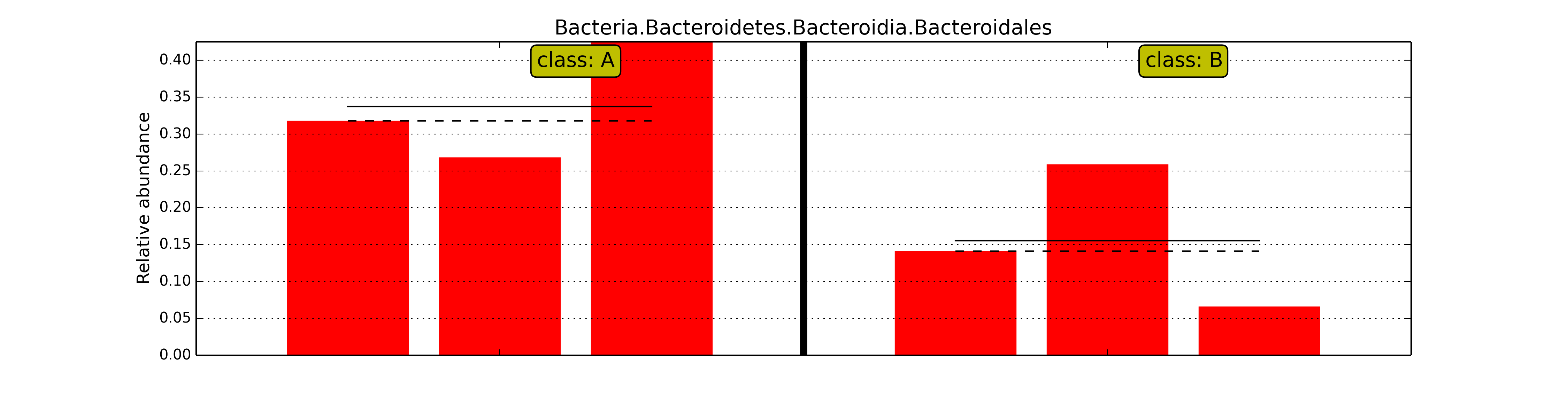

Supplement: Supplemental Information 1 [file peerj-08-9698-s001.zip › C01_diff/lefse/lefse.png]

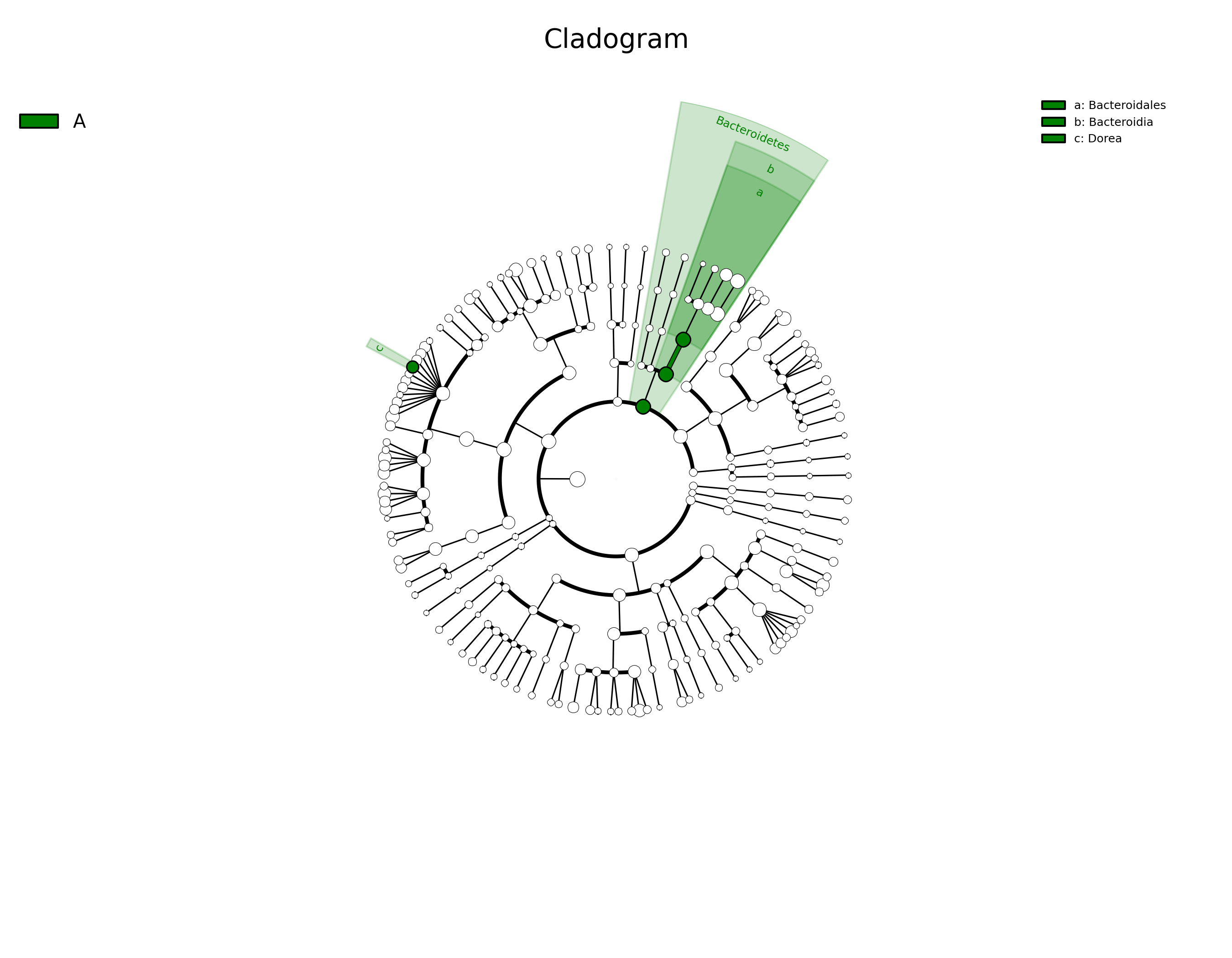

Supplement: Supplemental Information 1 [file peerj-08-9698-s001.zip › C01_diff/lefse/lefse_cladogram.png]

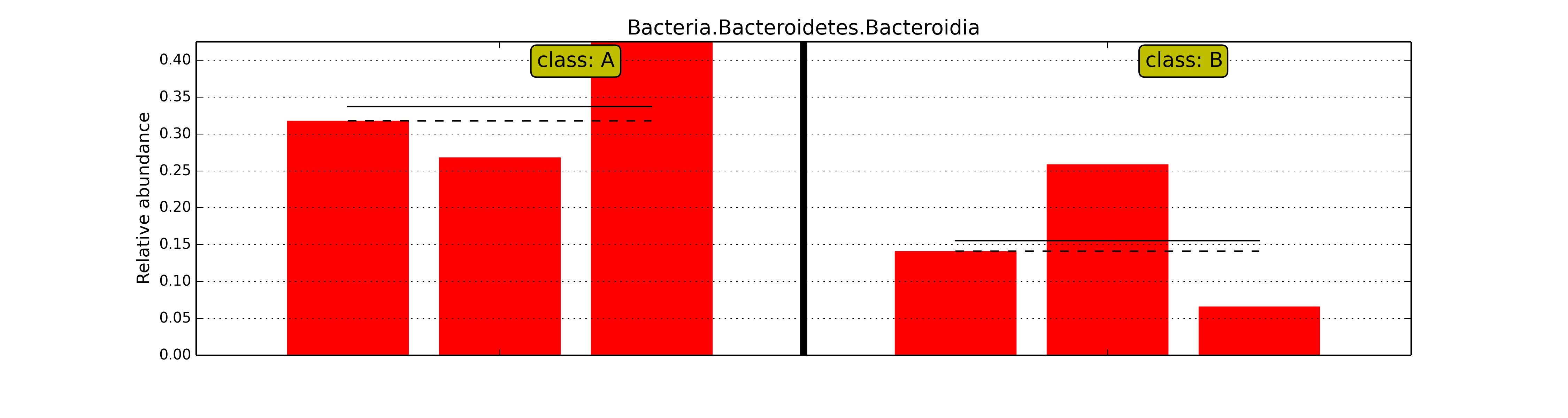

Supplement: Supplemental Information 1 [file peerj-08-9698-s001.zip › C01_diff/lefse/lefse_diff_taxa/1_Bacteria-Bacteroidetes-Bacteroidia.png]

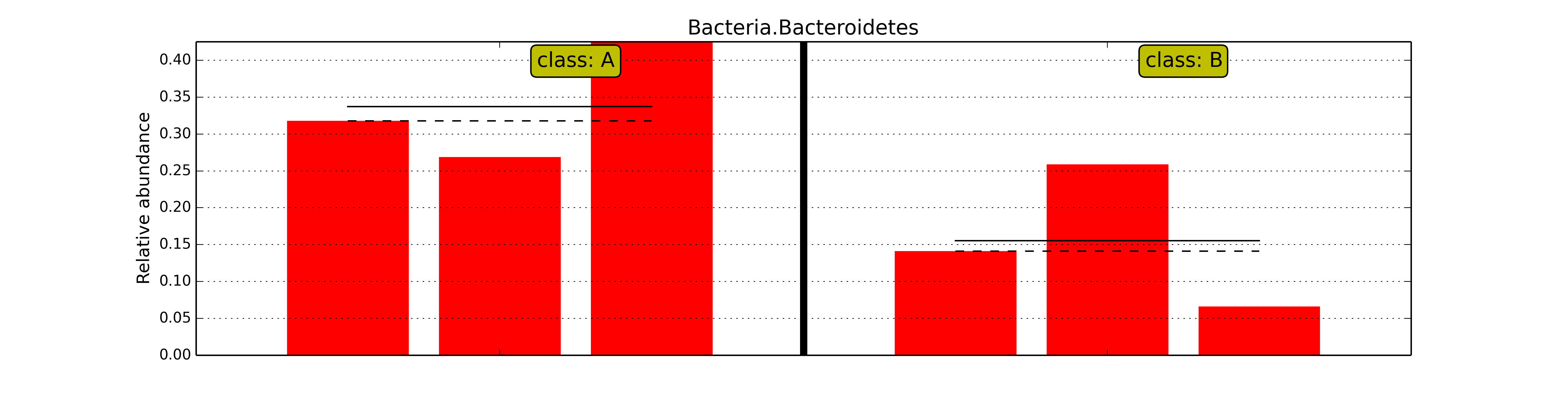

Supplement: Supplemental Information 1 [file peerj-08-9698-s001.zip › C01_diff/lefse/lefse_diff_taxa/1_Bacteria-Bacteroidetes.png]

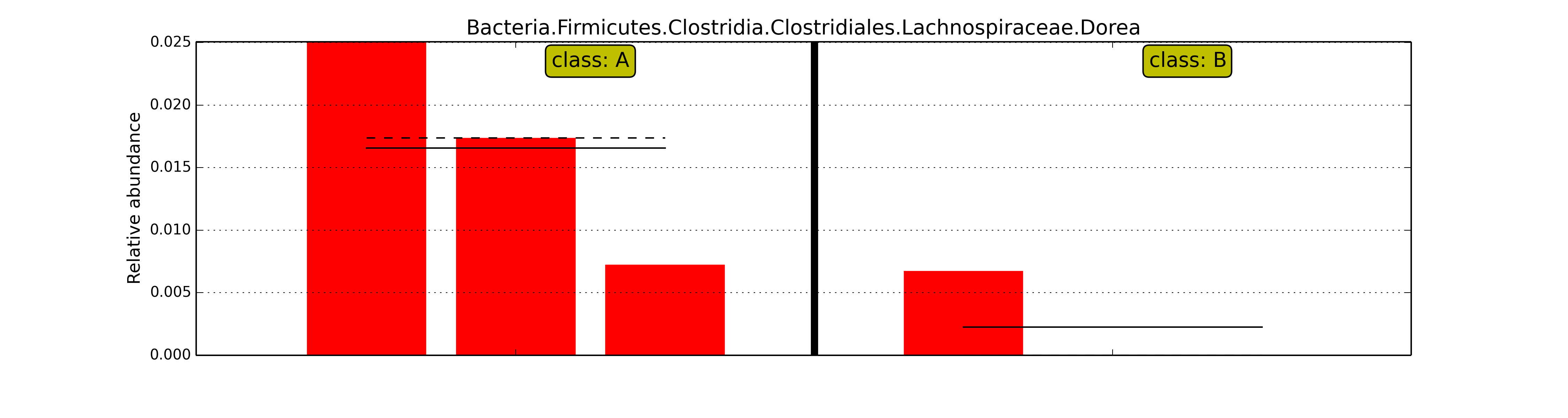

Supplement: Supplemental Information 1 [file peerj-08-9698-s001.zip › C01_diff/lefse/lefse_diff_taxa/1_Bacteria-Firmicutes-Clostridia-Clostridiales-Lachnospiraceae-Dorea.png]

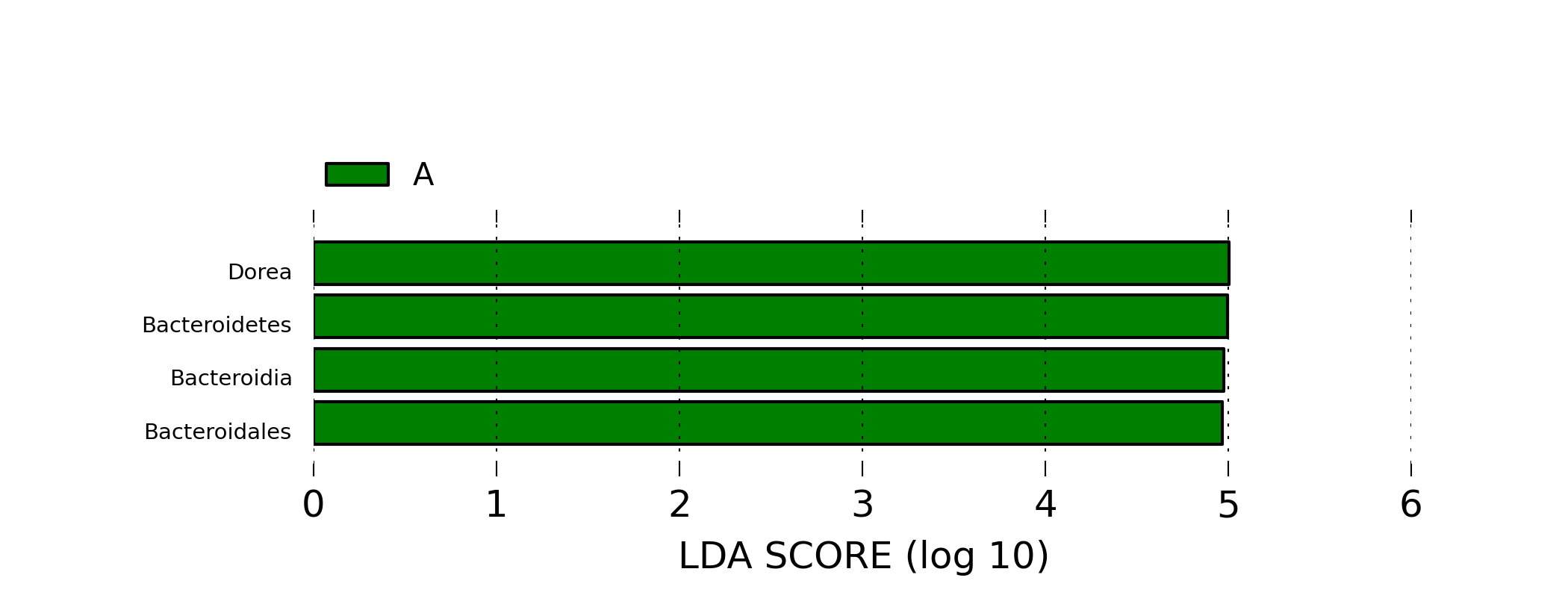

Supplement: Supplemental Information 1 [file peerj-08-9698-s001.zip › C01_diff/lefse/lefse_effect_size_rank.png]

Dorea

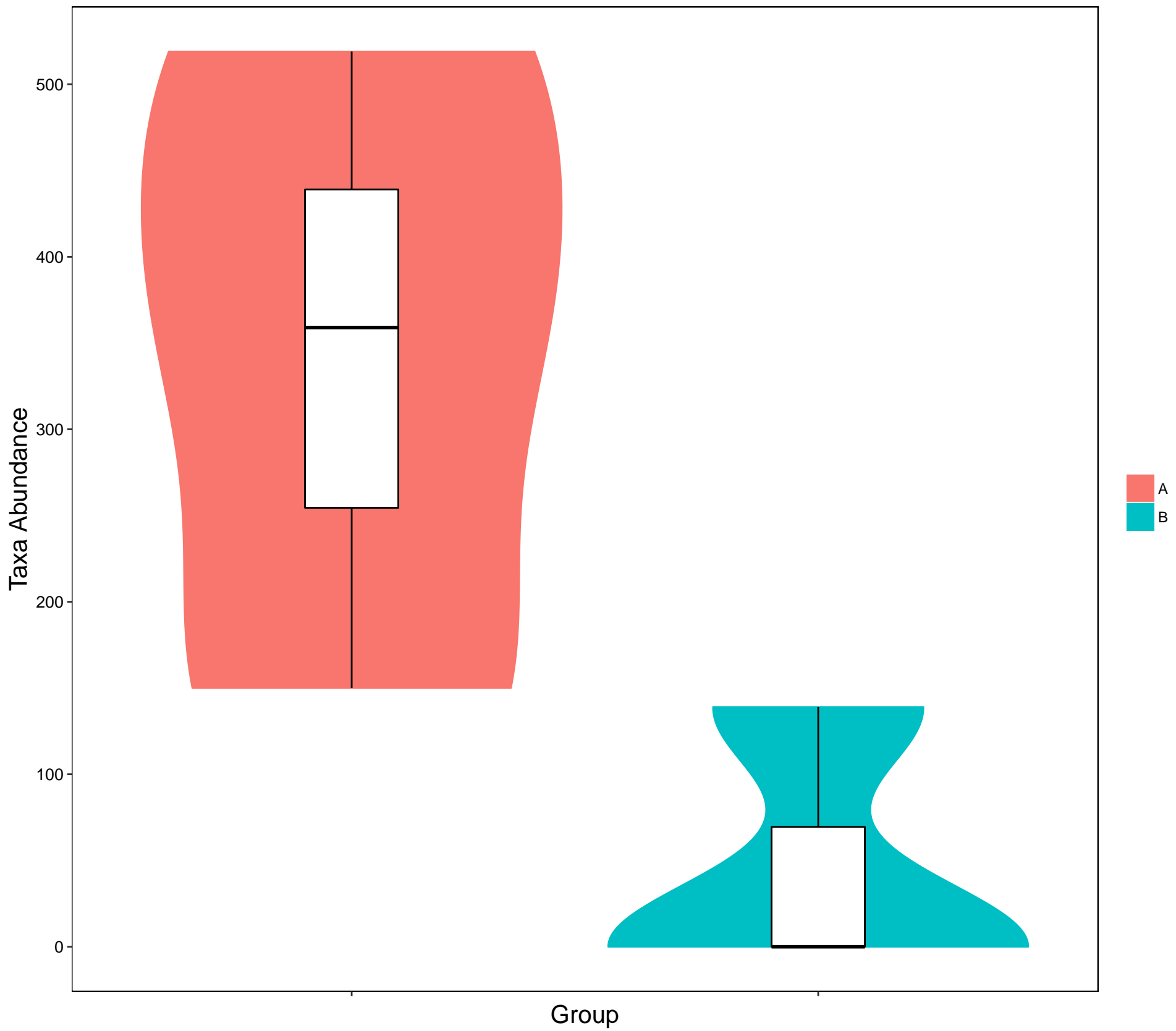

Supplement: Supplemental Information 1 [file peerj-08-9698-s001.zip › C01_diff/metastats/diff_group_genus.pdf]

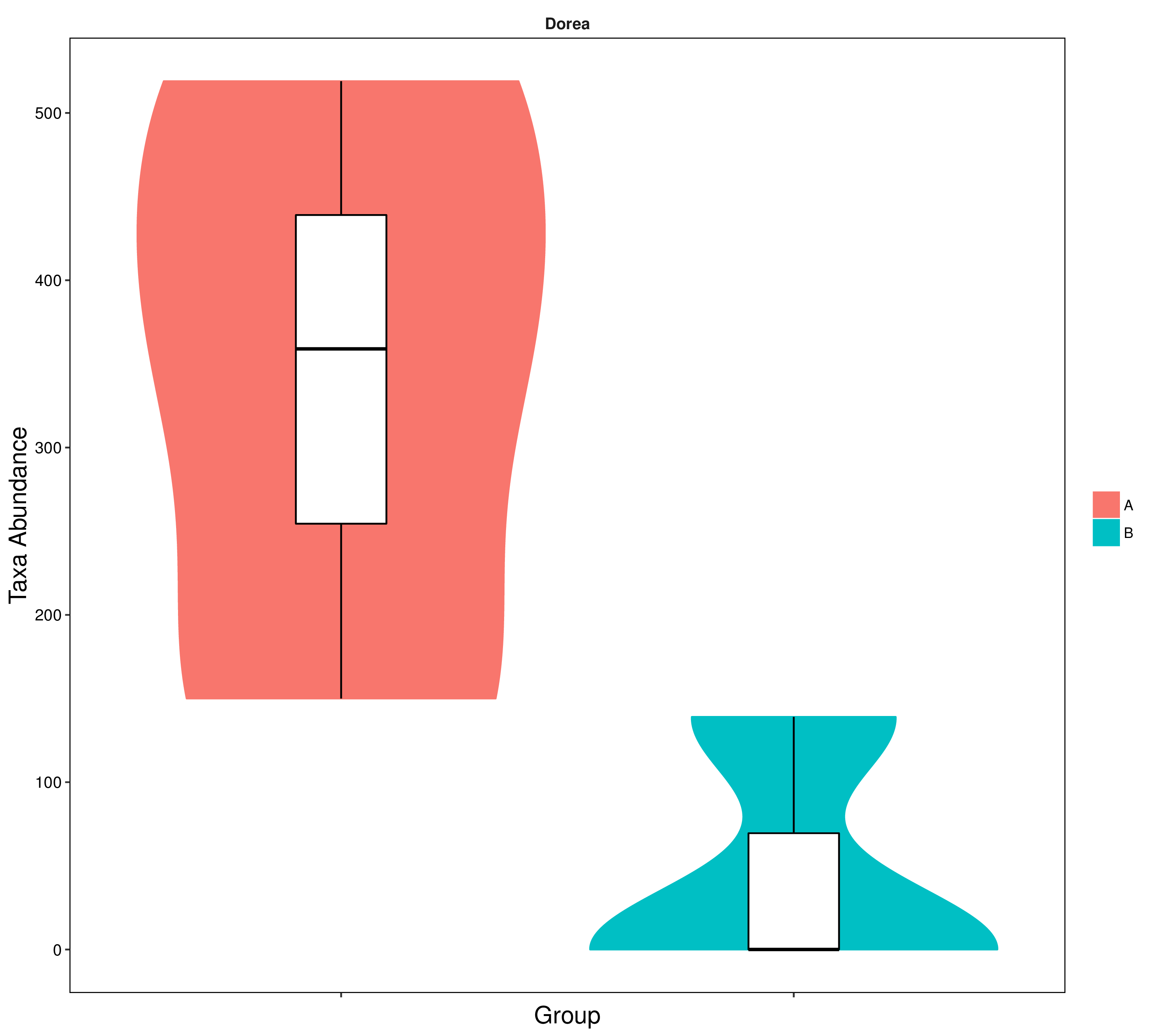

Supplement: Supplemental Information 1 [file peerj-08-9698-s001.zip › C01_diff/metastats/diff_group_genus.png]

# Bacteroidetes

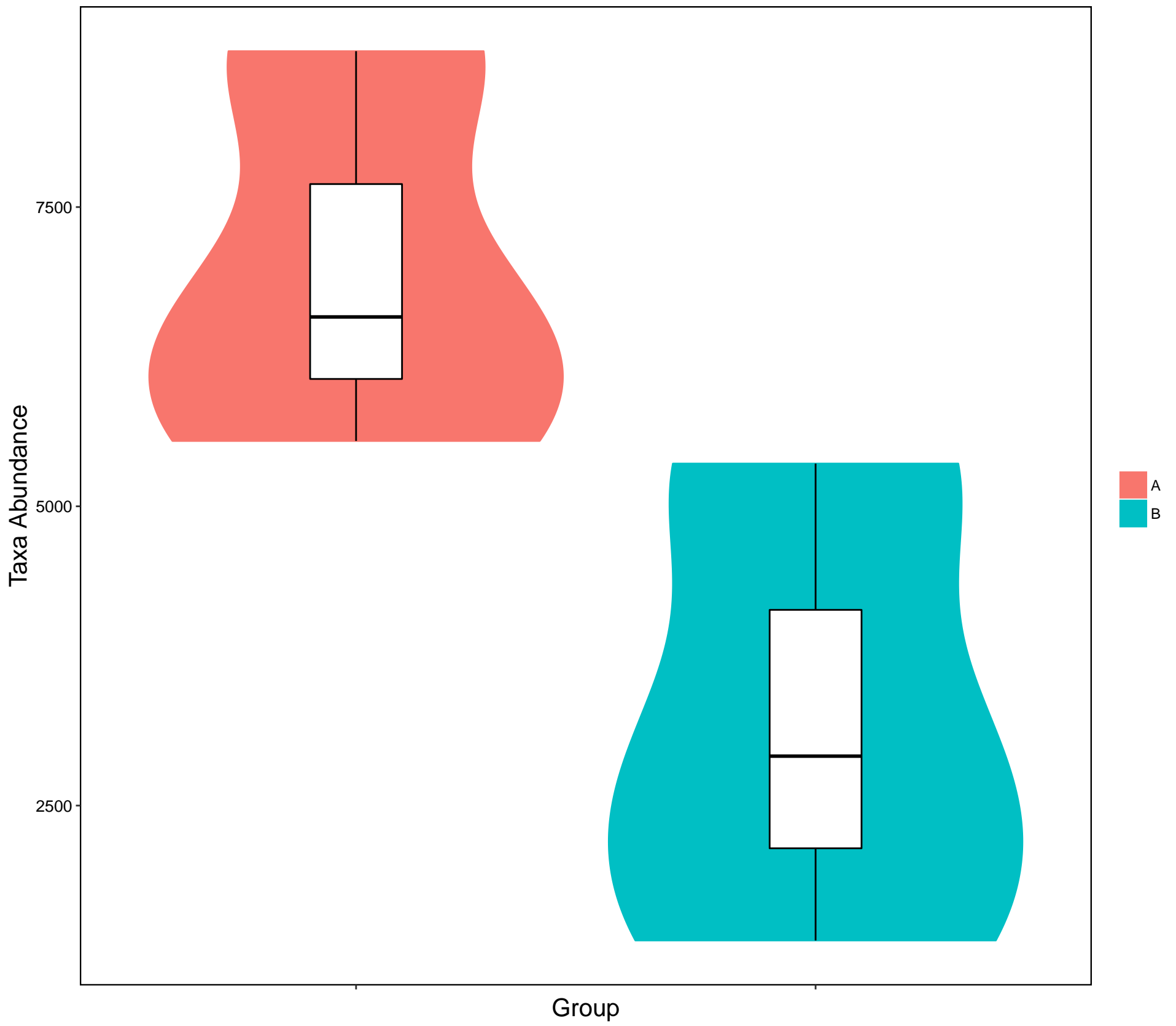

Supplement: Supplemental Information 1 [file peerj-08-9698-s001.zip › C01_diff/metastats/diff_group_phylum.pdf]

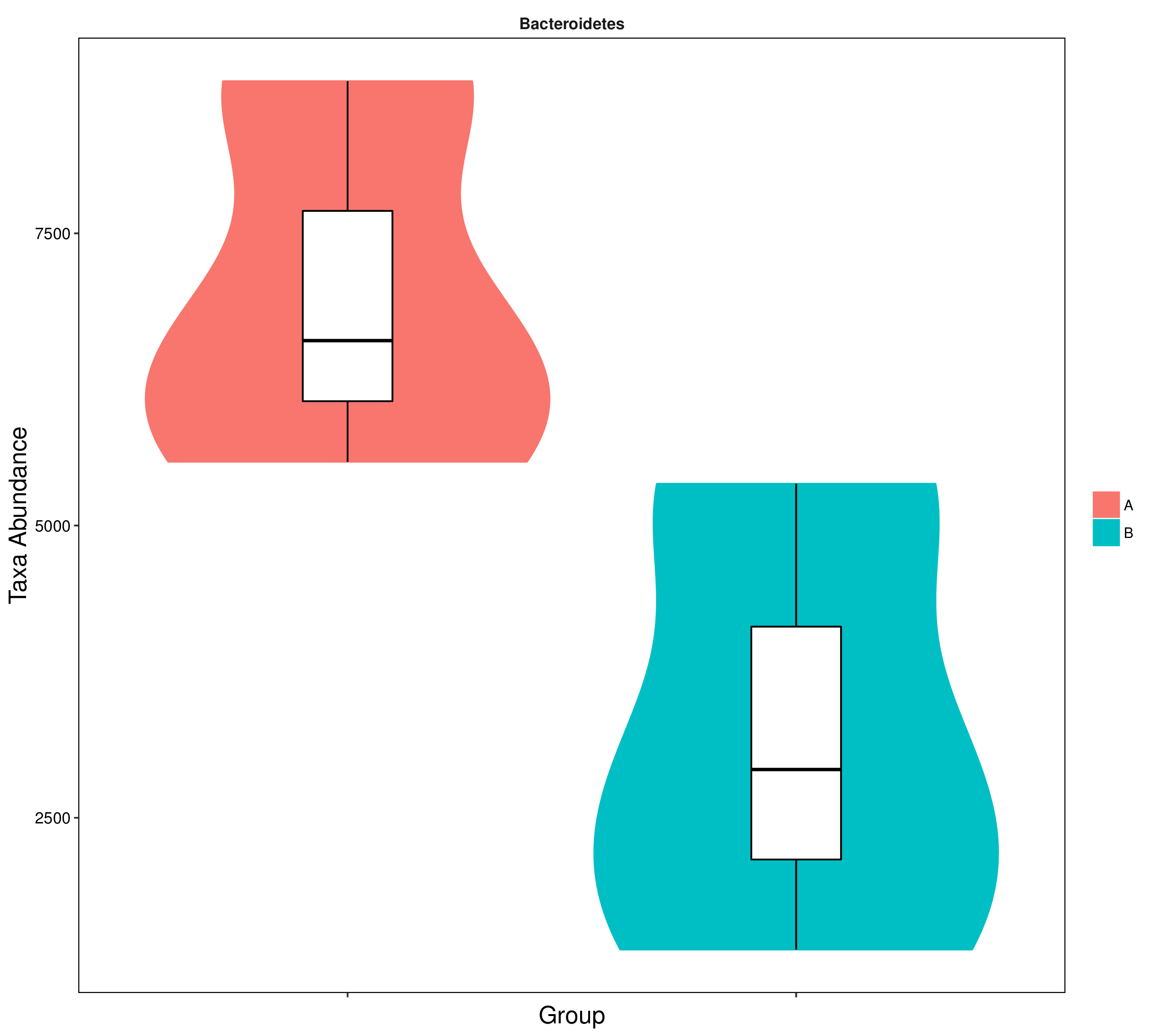

Supplement: Supplemental Information 1 [file peerj-08-9698-s001.zip › C01_diff/metastats/diff_group_phylum.png]
